# Supplementary material for: Systemic Antibiotics as an Adjunct to Subgingival Debridement: A Network Meta-Analysis
Source: Antibiotics (Basel). 2022 Nov 28;11(12):1716. doi: 10.3390/antibiotics11121716 (PMC9774554; doi:10.3390/antibiotics11121716)
Supplement: Supplementary file 1 [file antibiotics-11-01716-s001.zip › antibiotics-2022986-supplementary.pdf]

Supplementary Materials

Systemic Antibiotics as Adjunct to Subgingival Debridement: A Network Meta-Analysis

Ainol Haniza Kherul Anuwar, Mohd Syukri Mohd Taib, Rokiah Mamikutty, Roslan Saub, Syarida Hasnur Safii, Norintan Ab-Murat, and Chiu Wan Ng

Table S1. Search strategies for each electronic database

No.	Date	Database	Search strategy
1.	14.09.2020<br>Updated on<br>11.09.2021	PubMed	1. periodontitis [MeSH Terms] OR chronic periodontitis [MeSH Terms] OR aggressive periodontitis [MeSH Terms] OR pericementitides [Title/Abstract] OR pericementitis [Title/Abstract]<br>2. periodontitides [Title/Abstract] OR periodontitis [Title/Abstract] OR adult periodontitides [Title/Abstract] OR adult periodontitis [Title/Abstract] OR chronic periodontitides [Title/Abstract] OR chronic periodontitis [Title/Abstract] OR aggressive periodontitis [Title/Abstract] OR circumpubertal periodontitis [Title/Abstract] OR early onset periodontitis [Title/Abstract] OR early-onset periodontitides [Title/Abstract] OR early-onset periodontitis [Title/Abstract] OR juvenile periodontitides [Title/Abstract] OR juvenile periodontitis [Title/Abstract] OR prepubertal periodontitis [Title/Abstract] OR periodontoses [Title/Abstract] OR periodontosis [Title/Abstract]<br>3. anti-bacterial agents [MeSH Terms] OR penicillins [MeSH Terms] OR cephalosporins [MeSH Terms] OR amoxicillin [MeSH Terms] OR amoxicillin-potassium clavulanate combination [MeSH Terms] OR azithromycin [MeSH Terms] OR clarithromycin [MeSH Terms] OR metronidazole [MeSH Terms] OR moxifloxacin [MeSH Terms] OR spiramycin [MeSH Terms] OR tetracycline [MeSH Terms]<br>4. anti bacterial agents [Title/Abstract] OR anti-bacterial agents [Title/Abstract] OR anti mycobacterial agents [Title/Abstract] OR anti-mycobacterial agents [Title/Abstract] OR antimycobacterial agents [Title/Abstract] OR antibacterial agents [Title/Abstract] OR bacteriocidal agents [Title/Abstract] OR anti-bacterial compounds [Title/Abstract] OR anti bacterial compounds [Title/Abstract] OR antibiotic [Title/Abstract] OR antibiotics [Title/Abstract] OR bacteriocides [Title/Abstract] OR penicillin antibiotics [Title/Abstract] OR penicillin [Title/Abstract] OR penicillins [Title/Abstract] OR cephalosporin antibiotics [Title/Abstract] OR cephalosporanic acids [Title/Abstract] OR amoxycillin [Title/Abstract] OR amoxicilline [Title/Abstract] OR amoxicillin monopotassium salt [Title/Abstract] OR amoxicillin sodium [Title/Abstract] OR amoxicillin monosodium salt [Title/Abstract] OR amoxicillin anhydrous [Title/Abstract] OR amoxicillin trihydrate [Title/Abstract] OR hydroxyampicillin [Title/Abstract] OR actimoxiclamoxyl [Title/Abstract] OR penamox [Title/Abstract] OR polymox [Title/Abstract] OR trimox [Title/Abstract] OR wymox [Title/Abstract] OR wymox [Title/Abstract] OR amoxicillin potassium clavulanate combination [Title/Abstract] OR co-amoxiclav [Title/Abstract] OR co amoxiclav [Title/Abstract] OR coamoxiclav [Title/Abstract] OR amoxicillin-clavulanic acid [Title/Abstract] OR amoxicillin clavulanic acid [Title/Abstract] OR amoxi-clavulanate [Title/Abstract] OR amoxi clavulanate [Title/Abstract] OR potassium clavulanate-amoxicillin combination [Title/Abstract] OR potassium clavulanate amoxicillin combination [Title/Abstract] OR amoxycillin-clavulanic acid [Title/Abstract] OR amoxycillin clavulanic acid [Title/Abstract] OR amox-clav [Title/Abstract] OR amox clav [Title/Abstract] OR clavulanate potentiated amoxycillin [Title/Abstract] OR augmentin [Title/Abstract] OR synulox [Title/Abstract] OR spektramox [Title/Abstract] OR clavulin [Title/Abstract] OR Azithromycin [Title/Abstract] OR sumamed [Title/Abstract] OR toraseptol [Title/Abstract] OR vinzam [Title/Abstract] OR Zithromax [Title/Abstract] OR Azitrocin [Title/Abstract] OR

No.	Date	Database	Search strategy
			Azadose [Title/Abstract] OR Ultreon [Title/Abstract] OR Zitromax [Title/Abstract] OR azithromycin dihydrate [Title/Abstract] OR azithromycin monohydrate [Title/Abstract] OR goxal [Title/Abstract] OR zentavion [Title/Abstract] OR clarithromycin [Title/Abstract] OR biaxin [Title/Abstract] OR doxycycline monohydrate [Title/Abstract] OR doxycycline hyclate [Title/Abstract] OR doxycycline hemiethanolate [Title/Abstract] OR doxycycline calcium [Title/Abstract] OR doxycycline chinoin [Title/Abstract] OR vibramycin [Title/Abstract] OR atridox [Title/Abstract] OR doryx [Title/Abstract] OR doxycycline-chinoin [Title/Abstract] OR hydramycin [Title/Abstract] OR oracea [Title/Abstract] OR periostat [Title/Abstract] OR vibra-tabs [Title/Abstract] OR vibra tabs [Title/Abstract] OR vibramycin novum [Title/Abstract] OR vibravenos [Title/Abstract] OR trichazol [Title/Abstract] OR trichopo [Title/Abstract] OR trivazol [Title/Abstract] OR metronidazole phosphoester [Title/Abstract] OR vagilen [Title/Abstract] OR metronidazole phosphate [Title/Abstract] OR danazol [Title/Abstract] OR flagyl [Title/Abstract] OR gineflavir [Title/Abstract] OR metric [Title/Abstract] OR metrodzhil [Title/Abstract] OR metrogel [Title/Abstract] OR metrogyl [Title/Abstract] OR metronidazole hydrochloride [Title/Abstract] OR metronidazole monohydrochloride [Title/Abstract] OR clont [Title/Abstract] OR octegra [Title/Abstract] OR proflox [Title/Abstract] OR moxifloxacin hydrochloride [Title/Abstract] OR avelox [Title/Abstract] OR avalox [Title/Abstract] OR izilox [Title/Abstract] OR actira [Title/Abstract] OR spiramycine [Title/Abstract] OR rovamycine [Title/Abstract] OR selectomycin [Title/Abstract] OR rovamycin [Title/Abstract] OR spiramycin adipate [Title/Abstract] OR Tetrabid [Title/Abstract] OR Topicycline [Title/Abstract] OR achromycin v [Title/Abstract] OR hostacyclin [Title/Abstract] OR tetracycline hydrochloride [Title/Abstract] OR tetracycline monohydrochloride [Title/Abstract] OR sustamycin [Title/Abstract] OR achromycin [Title/Abstract]<br>5. 1 or 2<br>6. 3 or 4<br>7. 5 and 6; Filters: Meta-Analysis, Randomized Controlled Trial, Systematic Review, from 2000 - current
2.	13.09.2021	MEDLINE Complete via EBSCOhost	1. MH periodontitis [mesh] OR MH chronic periodontitis OR MW aggressive periodontitis<br>2. AB pericementitides OR AB pericementitis OR AB periodontitides OR AB periodontitis OR AB adult periodontitis OR AB adult periodontitides OR AB chronic periodontitides OR AB chronic periodontitis OR AB aggressive periodontitis OR AB circumpubertal periodontitis OR AB early onset periodontitis OR AB early-onset periodontitides<br>3. AB early-onset periodontitis OR AB juvenile periodontitides OR AB juvenile periodontitis OR AB prepubertal periodontitis OR AB periodontoses OR AB periodontosis<br>4. S1 OR S2 OR S3<br>5. MH anti-bacterial agents OR MH penicillins OR MH cephalosporins OR MH amoxicillin OR MH amoxicillin-potassium clavulanate combination OR MH azithromycin OR MH clarithromycin OR MH doxycycline OR MH metronidazole OR MH moxifloxacin OR MH spiromycin OR MH tetracycline<br>6. AB anti-bacterial agents OR AB anti bacterial agents OR AB anti mycobacterial agents OR AB anti-mycobacterial agents OR AB antimycobacterial agents OR AB antibacterial agents OR AB bacteriocidal agents OR AB anti-bacterial compounds OR AB anti bacterial compounds OR AB antibiotics OR AB antibiotic OR AB bacteriocides<br>7. AB penicillin antibiotics OR AB penicillin OR AB penicillins OR AB cephalosporin antibiotics OR AB cephalosporanic acids OR AB amoxycillin OR AB amoxicilline OR AB amoxicillin monopotassium salt OR AB amoxicillin sodium OR AB amoxicillin monosodium salt OR AB amoxicillin anhydrous OR AB amoxicillin trihydrate<br>8. AB hydroxyampicillin OR AB actimoxiclamoxyl OR AB penamox OR AB polymox OR AB trimox OR AB wymox OR AB amoxil OR AB amoxicillin potassium clavulanate combination OR AB co-amoxiclav OR AB co amoxiclav OR AB coamoxiclav OR AB amoxicillin-clavulanic acid

No.	Date	Database	Search strategy
			9. AB amoxicillin clavulanic acid OR AB amoxi-clavulanate OR AB amoxi clavulanate OR AB potassium clavulanate-amoxicillin combination OR AB potassium clavulanate amoxicillin combination OR AB amoxycillin-clavulanic acid OR AB amoxycillin clavulanic acid OR AB amox-clav OR AB amox clav OR AB clavulanate potentiated amoxycillin OR AB augmentin OR AB synulox<br>10. AB spektramox OR AB clavulin OR AB Azithromycin OR AB sumamed OR AB toraseptol OR AB vinzam OR AB Zithromax OR AB Azitrocin OR AB Azadose OR AB Ultreon OR AB Zitromax OR AB azithromycin dihydrate<br>11. AB azithromycin monohydrate OR AB goxal OR AB zentavion OR AB clarithromycin OR AB biaxin OR AB doxycycline monohydrate OR AB doxycycline hyclate OR AB doxycycline hemiethanolate OR AB doxycycline calcium OR AB doxycycline chinoin OR AB vibramycin OR AB atridox<br>12. AB doryx OR AB doxycycline-chinoin OR AB hydramycin OR AB oracea OR AB periostat OR AB vibra-tabs OR AB vibra tabs OR AB vibramycin novum OR AB vibravenos OR AB trichazol OR AB trichopol OR AB trivazol<br>13. AB metronidazole phosphoester OR AB vagilen OR AB metronidazole phosphate OR AB danazol OR AB flagyl OR AB gineflavir OR AB metric OR AB metrodzhil OR AB metrogel OR AB metrogyl OR AB metronidazole hydrochloride OR AB metronidazole monohydrochloride<br>14. AB clont OR AB octegra OR AB proflox OR AB moxifloxacin hydrochloride OR AB avelox OR AB avalox OR AB izilox OR AB actira OR AB spiramycine OR AB rovamycine OR AB selectomycin OR AB rovamycin<br>15. AB spiramycin adipate OR AB Tetrabid OR AB Topicycline OR AB achromycin v OR AB hostacyclin OR AB tetracycline hydrochloride OR AB tetracycline monohydrochloride OR AB sustamycin OR AB achromycin<br>16. S5 OR S6 OR S7 OR S8 OR S9 OR S10 OR S11 OR S12 OR S13 OR S14 OR S15<br>17. S4 AND S16<br>18. S4 AND S16; Date of Publication: 20000101-20211231; English Language; Human 1,384<br>19. S4 AND S16; Limiters - Date of Publication: 20000101-20211231; English Language; Publication Type: Randomized Controlled Trial
3.	13.09.2021	CINAHL Complete via EBSCOhost	1. MH periodontitis [mesh] OR MH chronic periodontitis OR MW aggressive periodontitis<br>2. AB pericementitides OR AB pericementitis OR AB periodontitides OR AB periodontitis OR AB adult periodontitis OR AB adult periodontitides OR AB chronic periodontitides OR AB chronic periodontitis OR AB aggressive periodontitis OR AB circumpubertal periodontitis OR AB early onset periodontitis OR AB early-onset periodontitides<br>3. AB early-onset periodontitis OR AB juvenile periodontitides OR AB juvenile periodontitis OR AB prepubertal periodontitis OR AB periodontoses OR AB periodontosis<br>4. S1 OR S2 OR S3<br>5. MH anti-bacterial agents OR MH penicillins OR MH cephalosporins OR MH amoxicillin OR MH amoxicillin-potassium clavulanate combination OR MH azithromycin OR MH clarithromycin OR MH doxycycline OR MH metronidazole OR MH moxifloxacin OR MH spiromycin OR MH tetracycline<br>6. AB anti-bacterial agents OR AB anti bacterial agents OR AB anti mycobacterial agents OR AB anti-mycobacterial agents OR AB antimycobacterial agents OR AB antibacterial agents OR AB bacteriocidal agents OR AB anti-bacterial compounds OR AB anti bacterial compounds OR AB antibiotics OR AB antibiotic OR AB bacteriocides<br>7. AB penicillin antibiotics OR AB penicillin OR AB penicillins OR AB cephalosporin antibiotics OR AB cephalosporanic acids OR AB amoxycillin OR AB amoxicilline OR AB amoxicillin monopotassium salt OR AB amoxicillin sodium OR AB amoxicillin monosodium salt OR AB amoxicillin anhydrous OR AB amoxicillin trihydrate<br>8. AB hydroxyampicillin OR AB actimoxiclamoxyl OR AB penamox OR AB polymox OR AB trimox OR AB wymox OR AB amoxil OR AB amoxicillin potassium clavulanate combination OR AB co-amoxiclav OR AB co amoxiclav OR AB coamoxiclav OR AB amoxicillin-clavulanic acid

No.	Date	Database	Search strategy
			9. AB amoxicillin clavulanic acid OR AB amoxi-clavulanate OR AB amoxi clavulanate OR AB potassium clavulanate-amoxicillin combination OR AB potassium clavulanate amoxicillin combination OR AB amoxycillin-clavulanic acid OR AB amoxycillin clavulanic acid OR AB amox-clav OR AB amox clav OR AB clavulanate potentiated amoxycillin OR AB augmentin OR AB synulox<br>10. AB spektramox OR AB clavulin OR AB Azithromycin OR AB sumamed OR AB toraseptol OR AB vinzam OR AB Zithromax OR AB Azitrocin OR AB Azadose OR AB Ultreon OR AB Zitromax OR AB azithromycin dihydrate<br>11. AB azithromycin monohydrate OR AB goxal OR AB zentavion OR AB clarithromycin OR AB biaxin OR AB doxycycline monohydrate OR AB doxycycline hyclate OR AB doxycycline hemiethanolate OR AB doxycycline calcium OR AB doxycycline chinoin OR AB vibramycin OR AB atridox<br>12. AB doryx OR AB doxycycline-chinoin OR AB hydramycin OR AB oracea OR AB periostat OR AB vibra-tabs OR AB vibra tabs OR AB vibramycin novum OR AB vibravenos OR AB trichazol OR AB trichopol OR AB trivazol<br>13. AB metronidazole phosphoester OR AB vagilen OR AB metronidazole phosphate OR AB danazol OR AB flagyl OR AB gineflavir OR AB metric OR AB metrodzhil OR AB metrogel OR AB metrogyl OR AB metronidazole hydrochloride OR AB metronidazole monohydrochloride<br>14. AB clont OR AB octegra OR AB proflox OR AB moxifloxacin hydrochloride OR AB avelox OR AB avalox OR AB izilox OR AB actira OR AB spiramycine OR AB rovamycine OR AB selectomycin OR AB rovamycin<br>15. AB spiramycin adipate OR AB Tetrabid OR AB Topicycline OR AB achromycin v OR AB hostacyclin OR AB tetracycline hydrochloride OR AB tetracycline monohydrochloride OR AB sustamycin OR AB achromycin<br>16. S5 OR S6 OR S7 OR S8 OR S9 OR S10 OR S11 OR S12 OR S13 OR S14 OR S15<br>17. S4 AND S16<br>18. S4 AND S16; Limiters: Date of Publication: 20000101-20211231; English Language; Human
4.	13.09.2021	Dentistry & Oral Sciences via EBSCOhost	1. MH periodontitis [mesh] OR MH chronic periodontitis OR MW aggressive periodontitis<br>2. AB pericementitides OR AB pericementitis OR AB periodontitides OR AB periodontitis OR AB adult periodontitis OR AB adult periodontitides OR AB chronic periodontitides OR AB chronic periodontitis OR AB aggressive periodontitis OR AB circumpubertal periodontitis OR AB early onset periodontitis OR AB early-onset periodontitides<br>3. AB early-onset periodontitis OR AB juvenile periodontitides OR AB juvenile periodontitis OR AB prepubertal periodontitis OR AB periodontoses OR AB periodontosis<br>4. S1 OR S2 OR S3<br>5. MH anti-bacterial agents OR MH penicillins OR MH cephalosporins OR MH amoxicillin OR MH amoxicillin-potassium clavulanate combination OR MH azithromycin OR MH clarithromycin OR MH doxycycline OR MH metronidazole OR MH moxifloxacin OR MH spiromycin OR MH tetracycline<br>6. AB anti-bacterial agents OR AB anti bacterial agents OR AB anti mycobacterial agents OR AB anti-mycobacterial agents OR AB antimycobacterial agents OR AB antibacterial agents OR AB bacteriocidal agents OR AB anti-bacterial compounds OR AB anti bacterial compounds OR AB antibiotics OR AB antibiotic OR AB bacteriocides<br>7. AB penicillin antibiotics OR AB penicillin OR AB penicillins OR AB cephalosporin antibiotics OR AB cephalosporanic acids OR AB amoxycillin OR AB amoxicilline OR AB amoxicillin monopotassium salt OR AB amoxicillin sodium OR AB amoxicillin monosodium salt OR AB amoxicillin anhydrous OR AB amoxicillin trihydrate<br>8. AB hydroxyampicillin OR AB actimoxiclamoxyl OR AB penamox OR AB polymox OR AB trimox OR AB wymox OR AB amoxil OR AB amoxicillin potassium clavulanate combination OR AB co-amoxiclav OR AB co amoxiclav OR AB coamoxiclav OR AB amoxicillin-clavulanic acid

No.	Date	Database	Search strategy
			9. AB amoxicillin clavulanic acid OR AB amoxi-clavulanate OR AB amoxi clavulanate OR AB potassium clavulanate-amoxicillin combination OR AB potassium clavulanate amoxicillin combination OR AB amoxycillin-clavulanic acid OR AB amoxycillin clavulanic acid OR AB amox-clav OR AB amox clav OR AB clavulanate potentiated amoxycillin OR AB augmentin OR AB synulox<br>10. AB spektramox OR AB clavulin OR AB Azithromycin OR AB sumamed OR AB toraseptol OR AB vinzam OR AB Zithromax OR AB Azitrocin OR AB Azadose OR AB Ultreon OR AB Zitromax OR AB azithromycin dihydrate<br>11. AB azithromycin monohydrate OR AB goxal OR AB zentavion OR AB clarithromycin OR AB biaxin OR AB doxycycline monohydrate OR AB doxycycline hyclate OR AB doxycycline hemiethanolate OR AB doxycycline calcium OR AB doxycycline chinoin OR AB vibramycin OR AB atridox<br>12. AB doryx OR AB doxycycline-chinoin OR AB hydramycin OR AB oracea OR AB periostat OR AB vibra-tabs OR AB vibra tabs OR AB vibramycin novum OR AB vibravenos OR AB trichazol OR AB trichopol OR AB trivazol<br>13. AB metronidazole phosphoester OR AB vagilen OR AB metronidazole phosphate OR AB danazol OR AB flagyl OR AB gineflavir OR AB metric OR AB metrodzhil OR AB metrogel OR AB metrogyl OR AB metronidazole hydrochloride OR AB metronidazole monohydrochloride<br>14. AB clont OR AB octegra OR AB proflox OR AB moxifloxacin hydrochloride OR AB avelox OR AB avalox OR AB izilox OR AB actira OR AB spiramycine OR AB rovamycine OR AB selectomycin OR AB rovamycin<br>15. AB spiramycin adipate OR AB Tetrabid OR AB Topicycline OR AB achromycin v OR AB hostacyclin OR AB tetracycline hydrochloride OR AB tetracycline monohydrochloride OR AB sustamycin OR AB achromycin<br>16. S5 OR S6 OR S7 OR S8 OR S9 OR S10 OR S11 OR S12 OR S13 OR S14 OR S15<br>17. S4 AND S16<br>18. S4 AND S16; Limiters - Publication Date: 20000101-20211231
5.	13.09.2021	WOS	1. TS=pericementitides OR TS=pericementitis OR TS=periodontitides OR TS=periodontitis OR TS=adult periodontitides OR TS=adult periodontitis OR TS=chronic periodontitides OR TS=chronic periodontitis OR TS=aggressive periodontitis OR TS=circumpubertal periodontitis OR TS=early onset periodontitis OR TS=early-onset periodontitides OR TS=early-onset periodontitis OR TS=juvenile periodontitides OR TS=juvenile periodontitis OR TS=prepubertal periodontitis OR TS=periodontoses OR TS=periodontosis<br>2. TS=anti-bacterial agents OR TS=anti bacterial agents OR TS=anti mycobacterial agents OR TS=anti-mycobacterial agents OR TS=antimycobacterial agents OR TS=antibacterial agents OR TS=bacteriocidal agents OR TS=anti-bacterial compounds OR TS=anti bacterial compounds OR TS=antibiotic OR TS=antibiotics OR TS=bacteriocides OR TS=penicillin antibiotics OR TS=penicillin OR TS=penicillins OR TS=cephalosporin antibiotics OR TS=cephalosporanic acids OR TS=amoxycillin OR TS=amoxicilline OR TS=amoxicillin monopotassium salt OR TS=amoxicillin sodium OR TS=amoxicillin monosodium salt OR TS=amoxicillin anhydrous OR TS=amoxicillin trihydrate OR TS=hydroxyampicillin OR TS=actimoxiclamoxyl OR TS=penamox OR TS=polymox OR TS=trimox OR TS=wymox OR TS=amoxil OR TS=amoxicillin potassium clavulanate combination OR TS=co-amoxiclav OR TS=co amoxiclav OR TS=coamoxiclav OR TS=amoxicillin-clavulanic acid OR TS=amoxicillin clavulanic acid OR TS=amoxi-clavulanate OR TS=amoxi clavulanate OR TS=potassium clavulanate-amoxicillin combination OR TS=potassium clavulanate amoxicillin combination OR TS=amoxycillin-clavulanic acid OR TS=amoxycillin clavulanic acid OR TS=amox-clav OR TS=amox clav OR TS=clavulanate potentiated amoxycillin OR TS=augmentin OR TS=synulox OR TS=spektramox OR TS=clavulin OR TS=Azithromycin OR TS=sumamed OR TS=toraseptol OR TS=vinzam OR TS=Zithromax OR TS=Azitrocin OR TS=Azadose OR TS=Ultreon OR TS=zitromax OR TS=azithromycin dihydrate OR TS=azithromycin monohydrate OR TS=goal OR TS=zentavion OR TS=clarithromycin OR TS=biaxin OR TS=doxycycline monohydrate OR TS=doxycycline hyclate OR TS=doxycycline hemiethanolate OR TS=doxycycline calcium OR TS=doxycycline chinoin OR TS=vibramycin OR TS=atridox OR TS=doryx OR TS=doxycycline-chinoin OR TS=hydramycin OR TS=oracea OR TS=periostat OR TS=vibra-tabs OR TS=vibra tabs OR TS=vibramycin novum OR TS=vibravenos OR TS=trichazol OR TS=trichopol OR TS=trivazol OR TS=metronidazole phosphoester OR TS=danazol OR

No.	Date	Database	Search strategy
			TS=flagyl OR TS=gineflavir OR TS=metric OR TS=metrodzhil OR TS=metrogel OR TS=metrogyl OR TS=metronidazole hydrochloride OR TS=metronidazole monohydrochloride OR TS=clont OR TS=octegra OR TS=proflox OR TS=moxifloxacin hydrochloride OR TS=avelox OR TS=avalox OR TS=izilox OR TS=actira OR TS=spiramycine OR TS=rovamycine OR TS=selectomycin OR TS=rovamycin OR TS=spiramycin adipate OR TS=Tetrabid OR TS=Topicycline OR TS=achromycin v OR TS=hostacyclin OR TS=tetracycline hydrochloride OR TS=tetracycline monohydrochloride OR TS=sustamycin OR TS=achromycin<br>3. #1 AND #2<br>4. #1 AND #2 and 2000 or 2001 or 2002 or 2003 or 2004 or 2005 or 2006 or 2007 or 2008 or 2009 or 2010 or 2011 or 2012 or 2013 or 2014 or 2015 or 2016 or 2017 or 2018 or 2019 or 2020 or 2021 Publication Years and English Languages and Articles Document Types and Dentistry Oral Surgery Medicine Web of Science Categories
6.	13.09.2021	Scopus	1. TITLE-ABS pericementitides OR pericementitis OR periodontitides OR periodontitis OR "adult periodontitides" OR "adult periodontitis" OR "chronic periodontitides" OR "chronic periodontitis" OR "aggressive periodontitis" OR "circumpubertal periodontitis" OR "early onset periodontitis" OR "early-onset periodontitides" OR "early-onset periodontitis" OR "juvenile periodontitides" OR "juvenile periodontitis" OR "prepubertal periodontitis" OR periodontoses OR periodontosis<br>2. TITLE-ABS "anti bacterial agents" OR "anti-bacterial agents" OR "anti mycobacterial agents" OR "anti-mycobacterial agents" OR "antimycobacterial agents" OR "antibacterial agents" OR "bacteriocidal agents" OR "anti-bacterial compounds" OR "anti bacterial compounds" OR antibiotic OR antibiotics OR bacteriocides OR "penicillin antibiotics" OR penicillin OR penicillins OR "cephalosporin antibiotics" OR "cephalosporanic acids" OR amoxycillin OR amoxicilline OR "amoxicillin monopotassium salt" OR "amoxicillin sodium" OR "amoxicillin monosodium salt" OR "amoxicillin anhydrous" OR "amoxicillin trihydrate" OR hydroxyampicillin OR actimoxiclamoxyl OR penamox OR polymox OR trimox OR wymox OR amoxil OR "amoxicillin potassium clavulanate combination " OR co-amoxiclav OR "co amoxiclav" OR coamoxiclav OR "amoxicillin-clavulanic acid" OR "amoxicillin clavulanic acid" OR amoxi-clavulanate OR "amoxi clavulanate" OR "potassium clavulanate-amoxicillin combination" OR "potassium clavulanate amoxicillin combination" OR "amoxycillin-clavulanic acid" OR "amoxycillin clavulanic acid" OR amox-clav OR "amox clav" OR "clavulanate potentiated amoxycillin" OR augmentin OR synulox OR spektramox OR clavulin OR azithromycin OR sumamed OR toraseptol OR vinzam OR zithromax OR azitrocin OR azadose OR ultreon OR zitromax OR "azithromycin dihydrate" OR "azithromycin monohydrate" OR goxal OR zentavion OR clarithromycin OR biaxin OR "doxycycline monohydrate" OR "doxycycline hyclate" OR "doxycycline hemiethanolate" OR "doxycycline calcium" OR "doxycycline chinoin" OR vibramycin OR atridox OR doryx OR doxycycline-chinoin OR hydramycin OR oracea OR periostat OR vibra-tabs OR "vibra tabs" OR "vibramycin novum" OR vibravenos OR trichazol OR trichopol OR trivazol OR "metronidazole phosphoester" OR vagilen OR "metronidazole phosphate" OR danazol OR flagyl OR gineflavir OR metric OR metrodzhil OR metrogel OR metrogyl OR "metronidazole hydrochloride" OR "metronidazole monohydrochloride" OR clont OR octegra OR proflox OR "moxifloxacin hydrochloride" OR avelox OR avalox OR izilox OR actira OR spiramycine OR rovamycine OR selectomycin OR rovamycin OR "spiramycin adipate" OR tetrabid OR topicycline OR "achromycin v" OR hostacyclin OR "tetracycline hydrochloride" OR "tetracycline monohydrochloride" OR sustamycin OR achromycin<br>3. 1 AND 2 AND LIMIT-TO PUBYEAR , 2021 OR LIMIT-TO PUBYEAR , 2020 OR LIMIT-TO PUBYEAR , 2019 OR LIMIT-TO PUBYEAR , 2018 OR LIMIT-TO PUBYEAR , 2017 OR LIMIT-TO PUBYEAR , 2016 OR LIMIT-TO PUBYEAR , 2015 OR LIMIT-TO PUBYEAR , 2014 OR LIMIT-TO PUBYEAR , 2013 OR LIMIT-TO PUBYEAR , 2012 OR LIMIT-TO PUBYEAR , 2011 OR LIMIT-TO PUBYEAR , 2010 OR LIMIT-TO PUBYEAR , 2009 OR LIMIT-TO PUBYEAR , 2008 OR LIMIT-TO PUBYEAR , 2007 OR LIMIT-TO PUBYEAR , 2006 OR LIMIT-TO PUBYEAR , 2005 OR LIMIT-TO PUBYEAR , 2004 OR LIMIT-TO PUBYEAR , 2003 OR LIMIT-TO PUBYEAR , 2002 OR LIMIT-TO PUBYEAR , 2001 OR LIMIT-TO PUBYEAR , 2000 AND LIMIT-TO LANGUAGE , "English" AND LIMIT-TO SUBJAREA , "DENT"

No.	Date	Database	Search strategy
7.	13.09.2021	Cochrane Library	1. "anti bacterial agents" OR "anti-bacterial agents" OR "anti mycobacterial agents" OR "anti-mycobacterial agents" OR "antimycobacterial agents" OR "antibacterial agents" OR "bacteriocidal agents" OR "anti-bacterial compounds" OR "anti bacterial compounds" OR antibiotic OR antibiotics OR bacteriocides OR "penicillin antibiotics" OR penicillin OR penicillins OR "cephalosporin antibiotics" OR "cephalosporanic acids" OR amoxycillin OR amoxicilline OR "amoxicillin monopotassium salt" OR "amoxicillin sodium" OR "amoxicillin monosodium salt" OR "amoxicillin anhydrous" OR "amoxicillin trihydrate" OR hydroxyampicillin OR actimoxiclamoxyl OR penamox OR polymox OR trimox OR wymox OR amoxil OR "amoxicillin potassium clavulanate combination " OR co-amoxiclav OR "co amoxiclav" OR coamoxiclav OR "amoxicillin-clavulanic acid" OR "amoxicillin clavulanic acid" OR amoxi-clavulanate OR "amoxi clavulanate" OR "potassium clavulanate-amoxicillin combination" OR "potassium clavulanate amoxicillin combination" OR "amoxycillin-clavulanic acid" OR "amoxycillin clavulanic acid" OR amox-clav OR "amox clav" OR "clavulanate potentiated amoxycillin" OR augmentin OR synulox OR spektramox OR clavulin OR azithromycin OR sumamed OR toraseptol OR vinzam OR zithromax OR azitrocin OR azadose OR ultreon OR zitromax OR "azithromycin dihydrate" OR "azithromycin monohydrate" OR goxal OR zentavion OR clarithromycin OR biaxin OR "doxycycline monohydrate" OR "doxycycline hyclate" OR "doxycycline hemiethanolate" OR "doxycycline calcium" OR "doxycycline chinoin" OR vibramycin OR atridox OR doryx OR doxycycline-chinoin OR hydramycin OR oracea OR periostat OR vibra-tabs OR "vibra tabs" OR "vibramycin novum" OR vibravenos OR trichazol OR trichopol OR trivazol OR "metronidazole phosphoester" OR vagilen OR "metronidazole phosphate" OR danazol OR flagyl OR gineflavir OR metric OR metrodzhil OR metrogel OR metrogyl OR "metronidazole hydrochloride" OR "metronidazole monohydrochloride" OR clont OR octegra OR proflox OR "moxifloxacin hydrochloride" OR avelox OR avalox OR izilox OR actira OR spiramycine OR rovamycine OR selectomycin OR rovamycin OR "spiramycin adipate" OR tetrabid OR topicycline OR "achromycin v" OR hostacyclin OR "tetracycline hydrochloride" OR "tetracycline monohydrochloride" OR sustamycin OR achromycin:ti,ab,kw<br>2. pericementitides OR pericementitis OR periodontitides OR periodontitis OR "adult periodontitides" OR "adult periodontitis" OR "chronic periodontitides" OR "chronic periodontitis" OR "aggressive periodontitis" OR "circumpubertal periodontitis" OR "early onset periodontitis" OR "early-onset periodontitides" OR "early-onset periodontitis" OR "juvenile periodontitides" OR "juvenile periodontitis" OR "prepubertal periodontitis" OR periodontoses OR periodontosis:ti,ab,kw"<br>3. 1 AND 2; Word variations have been searched; Custom year range: 2000 – 2021
8.	13.09.2021	IMSEAR	1. tw:tw:pericementitides OR pericementitis OR periodontitides OR periodontitis OR "adult periodontitides" OR "adult periodontitis" OR "chronic periodontitides" OR "chronic periodontitis" OR "aggressive periodontitis" OR "circumpubertal periodontitis" OR "early onset periodontitis" OR "early-onset periodontitides" OR "early-onset periodontitis" OR "juvenile periodontitides" OR "juvenile periodontitis" OR "prepubertal periodontitis" OR periodontoses OR periodontosis<br>2. tw:"anti bacterial agents" OR "anti-bacterial agents" OR "anti mycobacterial agents" OR "anti-mycobacterial agents" OR "antimycobacterial agents" OR "antibacterial agents" OR "bacteriocidal agents" OR "anti-bacterial compounds" OR "anti bacterial compounds" OR antibiotic OR antibiotics OR bacteriocides OR "penicillin antibiotics" OR penicillin OR penicillins OR "cephalosporin antibiotics" OR "cephalosporanic acids" OR amoxycillin OR amoxicilline OR "amoxicillin monopotassium salt" OR "amoxicillin sodium" OR "amoxicillin monosodium salt" OR "amoxicillin anhydrous" OR "amoxicillin trihydrate" OR hydroxyampicillin OR actimoxiclamoxyl OR penamox OR polymox OR trimox OR wymox OR amoxil OR "amoxicillin potassium clavulanate combination " OR co-amoxiclav OR "co amoxiclav" OR coamoxiclav OR "amoxicillin-clavulanic acid" OR "amoxicillin clavulanic acid" OR amoxi-clavulanate OR "amoxi clavulanate" OR "potassium clavulanate-amoxicillin combination" OR "potassium clavulanate amoxicillin combination" OR "amoxycillin-clavulanic acid" OR "amoxycillin clavulanic acid" OR amox-clav OR "amox clav" OR "clavulanate potentiated amoxycillin" OR augmentin OR synulox OR spektramox OR clavulin OR azithromycin OR sumamed OR toraseptol OR vinzam OR zithromax OR azitrocin OR azadose OR ultreon OR zitromax OR "azithromycin dihydrate" OR "azithromycin monohydrate" OR goxal OR zentavion OR clarithromycin OR biaxin OR "doxycycline monohydrate" OR "doxycycline hyclate" OR "doxycycline hemiethanolate" OR "doxycycline calcium" OR "doxycycline

No.	Date	Database	Search strategy
			chinoin" OR vibramycin OR atridox OR doryx OR doxycycline-chinoin OR hydramycin OR oracea OR periostat OR vibra-tabs OR "vibra tabs" OR "vibramycin novum" OR vibravenos OR trichazol OR trichopol OR trivazol OR "metronidazole phosphoester" OR vagilen OR "metronidazole phosphate" OR danazol OR flagyl OR gineflavir OR metric OR metrodzhil OR metrogel OR metrogyl OR "metronidazole hydrochloride" OR "metronidazole monohydrochloride" OR clont OR octegra OR proflox OR "moxifloxacin hydrochloride" OR avelox OR avalox OR izilox OR actira OR spiramycine OR rovamycine OR selectomycin OR rovamycin OR "spiramycin adipate" OR tetrabid OR topicycline OR "achromycin v" OR hostacyclin OR "tetracycline hydrochloride" OR "tetracycline monohydrochloride" OR sustamycin OR achromycin<br>3. 1 AND 2 AND la:"en" AND year_cluster: [2000 TO 2021]

Table S2. Excluded studies and reasons for exclusion

No.	Authors (Year)	Journal	Title	Reason for exclusion
1.	Aimetti et al. (2012)	*J Clin Periodontol*	Full-mouth disinfection and systemic antimicrobial therapy in generalized aggressive periodontitis: a randomized, placebo-controlled trial.	Used of subgingival 1% CHX gel during NSPT and repeated after 8 days, and 0.2% CHX rinse twice a day for 1 min and to spray the tonsils twice daily with a 0.2% CHX for 2 months post-treatment
2.	Alyousef et al. (2017)	*Microb Pathog*	Chemically modified tetracyclines an emerging host modulator in chronic periodontitis patients: A randomized, double-blind, placebo-controlled, clinical trial.	Not commercially available (Chemically modified tetracycline; Incyclinide)
3.	Aral et al. (2019)	*Oral Dis*	Six-month clinical outcomes of non-surgical periodontal treatment with antibiotics on apoptosis markers in aggressive periodontitis.	Prospective interventional study
4.	Baltacioglu et al. (2011)	*J Can Dent Assoc*	Analysis of clinical results of systemic antimicrobials combined with nonsurgical periodontal treatment for generalized aggressive periodontitis: a pilot study.	Non-randomised clinical trial
5.	Bamedi et al. (2018)	*Implant Dent*	Evaluation the Effect of Adjuvant Therapy with Ciprofloxacin in Non-surgical Treatment of Periodontal Diseases.	Poster presentation abstract without full text
6.	Boia et al. (2018)	*Rom J Morphol Embryol*	Evaluation of antioxidant capacity and clinical assessment of patients with chronic periodontitis treated with non-surgical periodontal therapy and adjunctive systemic antibiotherapy	Non-randomised clinical trial
7.	Carvalho et al. (2004)	*J Clin Periodontol*	Scaling and root planing, systemic metronidazole and professional plaque removal in the treatment of chronic periodontitis in a Brazilian population. I. clinical results.	Insufficient outcome data

No.	Authors (Year)	Journal	Title	Reason for exclusion
8.	Casarin et al. (2012)	*J Periodontol*	The combination of amoxicillin and metronidazole improves clinical and microbiologic results of one-stage, full-mouth, ultrasonic debridement in aggressive periodontitis treatment.	Insufficient outcome data
9.	Caton et al. (2000)	*J Clin Periodontol*	Treatment with subantimicrobial dose doxycycline improves the efficacy of scaling and root planing in patients with adult periodontitis.	Discontinued adjunct (SDD 20mg)
10.	Caton et al. (2001)	*J Periodontol*	Treatment with subantimicrobial dose doxycycline improves the efficacy of scaling and root planing in patients with adult periodontitis.	Discontinued adjunct (SDD 20mg)
11.	Choi et al. (2004)	*J Periodontal Res*	Effects of sub-antimicrobial dose doxycycline therapy on crevicular fluid MMP-8, and gingival tissue MMP-9, TIMP-1 and IL-6 levels in chronic periodontitis.	Discontinued adjunct (SDD 20mg; Periostat)
12.	Cionca et al. (2009)	*J Periodontol*	Amoxicillin and metronidazole as an adjunct to full-mouth scaling and root planing of chronic periodontitis.	Used of 0.2% CHX bd for 10 days starting after first session of debridement
13.	Cionca et al. (2010)	*J Periodontol*	Microbiologic testing and outcomes of full-mouth scaling and root planing with or without amoxicillin/metronidazole in chronic periodontitis.	Used of 0.2% CHX bd for 10 days starting after first session of debridement
14.	Cortelli et al. (2008)	*J Oral Sci*	A double-blind randomized clinical trial of subgingival minocycline for chronic periodontitis.	The same trial and outcomes as Cortelli et al. (2006), focus on microbiologic findings.
15.	Cosgarea et al. (2017)	*PloS One*	One year results of a randomized controlled clinical study evaluating the effects of non-surgical periodontal therapy of chronic periodontitis in conjunction with three or seven days systemic administration of amoxicillin/metronidazole.	Used of 0.2% CHX digluconate solution and to brush their teeth with 0.2% CHX digluconate toothpaste for 14 days twice daily
16.	Cosgarea et al. (2016)	*J Clin Periodontol*	Non-surgical periodontal treatment in conjunction with 3 or 7 days systemic administration of amoxicillin and metronidazole in severe chronic periodontitis patients. A placebo-controlled randomized clinical study.	Used of 0.2% CHX digluconate solution and to brush their teeth with 0.2% CHX digluconate toothpaste for 14 days twice daily
17.	Emingil et al. (2004)	*J Periodontol*	The effect of adjunctive low-dose doxycycline therapy on clinical parameters and gingival crevicular fluid matrix metalloproteinase-8 levels in chronic periodontitis.	Discontinued adjunct (SDD 20mg; Periostat)
18.	Emingil et al. (2004b)	*J Periodontol*	Effectiveness of adjunctive low-dose doxycycline therapy on clinical parameters and gingival crevicular fluid laminin-5 gamma2 chain levels in chronic periodontitis.	The same RCT as Emingil et al. (2004a)
19.	Emingil et al. (2008)	*J Periodontol*	The effect of adjunctive subantimicrobial dose doxycycline therapy on GCF EMMPRIN levels in chronic periodontitis.	Discontinued adjunct (SDD 20mg; Periostat)

No.	Authors (Year)	Journal	Title	Reason for exclusion
20.	Emingil et al. (2006)	*Inflamm Res*	Adjunctive low-dose doxycycline therapy effect on clinical parameters and gingival crevicular fluid tissue plasminogen activator levels in chronic periodontitis.	Discontinued adjunct (SDD 20mg; Periostat)
21.	Emingil et al. (2011)	*J Periodontol*	Subantimicrobial-dose doxycycline and cytokine-chemokine levels in gingival crevicular fluid.	The same trial and outcomes as Emingil et al. (2006)
22.	Feres et al. (2001)	*J Clin Periodontol*	Change in subgingival microbial profiles in adult periodontitis subjects receiving either systemically-administered amoxicillin or metronidazole.	Non-randomised clinical trial
23.	Feres et al. (2012)	*J Clin Periodontol*	Metronidazole alone or with amoxicillin as adjuncts to non-surgical treatment of chronic periodontitis: a 1-year double-blinded, placebo-controlled, randomized clinical trial.	Used of CHX mouth rinse in combination with the intervention for some subjects
24.	Fujii et al. (2004)	*Perio*	Effect of Systemically Administered Azithromycin in Early Onset Aggressive Periodontitis.	Non-randomised clinical trial
25.	Golub et al. (2001)	*J Clin Periodontol*	Adjunctive treatment with subantimicrobial doses of doxycycline: effects on gingival fluid collagenase activity and attachment loss in adult periodontitis.	Discontinued adjunct (SDD 20mg)
26.	Goodson et al. (2012)	*J Clin Periodontol*	Control of periodontal infections: a randomized controlled trial I. The primary outcome attachment gain and pocket depth reduction at treated sites.	Used of 0.12% CHX twice daily mouth rinse during study period
27.	Guentsch et al. (2008)	*J Periodontol*	Moxifloxacin as an adjunctive antibiotic in the treatment of severe chronic periodontitis.	Treatment started with rinsing with 0.12% CHX
28.	Guerrero et al. (2005)	*J Clin Periodontol*	Adjunctive benefits of systemic amoxicillin and metronidazole in non-surgical treatment of generalized aggressive periodontitis: a randomized placebo-controlled clinical trial.	Used of 0.2% CHX rinse (supplied to improve compliance) bd for 2 weeks post-treatment
29.	Gürkan et al. (2005)	*J Clin Periodontol*	Adjunctive subantimicrobial dose doxycycline: effect on clinical parameters and gingival crevicular fluid transforming growth factor-beta levels in severe, generalized chronic periodontitis.	Discontinued adjunct (SDD 20mg; Periostat)
30.	Gürkan et al. (2008)	*Int J Dent Hyg*	Post-treatment effects of subantimicrobial dose doxycycline on clinical parameters and gingival crevicular fluid transforming growth factor-beta1 in severe, generalized chronic periodontitis.	Discontinued adjunct (SDD 20mg; Periostat)
31.	Haerian et al. (2012)	*J Periodontol Implant Dent*	Effectiveness of Adjunctive Subantimicrobial Dose Doxycycline on Phase I of Periodontal Therapy.	Discontinued adjunct (SDD 20mg; Periostat)
32.	Haffaje et al. (2007)	*J Clin Periodontol*	Clinical changes following four different periodontal therapies for the treatment of chronic periodontitis: 1-year results.	Insufficient outcome data

No.	Authors (Year)	Journal	Title	Reason for exclusion
33.	Heller et al. (2011)	*J Clin Periodontol*	Impact of systemic antimicrobials combined with anti-infective mechanical debridement on the microbiota of generalized aggressive periodontitis: a 6-month RCT.	Long-term used of CHX gel and solution
34.	Lee et al. (2004)	*J Periodontol*	Effect of subantimicrobial dose doxycycline as an effective adjunct to scaling and root planing.	Discontinued adjunct (SDD 20mg)
35.	Matthews et al. (2006)	*Evid Based Dent*	Adjunctive antibiotics in the treatment of generalized aggressive periodontitis.	Commentary of RCT by Guerrero et al. (2005)
36.	Mdala et al. (2012)	*J Oral Microbiol*	Multilevel analysis of clinical parameters in chronic periodontitis after root planing/scaling, surgery, and systemic and local antibiotics: 2-year results.	Insufficient outcome data
37.	Mestnik et al. (2010)	*J Clin Periodontol*	Short-term benefits of the adjunctive use of metronidazole plus amoxicillin in the microbial profile and in the clinical parameters of subjects with generalized aggressive periodontitis.	Used of 15 ml of 0.12% CHX bd for 60 days for 1 min. in the morning (30 min. after breakfast and tooth brushing) and at night (before going to sleep)
38.	Mestnik et al (2012)	*J Clin Periodontol*	The effects of adjunctive metronidazole plus amoxicillin in the treatment of generalized aggressive periodontitis: a 1-year double-blinded, placebo-controlled, randomized clinical trial.	Used of 15 ml of 0.12% CHX bd for 60 days for 1 min. in the morning (30 min. after breakfast and tooth brushing) and at night (before going to sleep)
39.	Morales et al. (2018)	*J Appl Oral Sci*	Microbiological and clinical effects of probiotics and antibiotics on nonsurgical treatment of chronic periodontitis: a randomized placebo-controlled trial with 9-month follow-up.	The same trial and outcomes as Morales et al. (2021)
40.	Novak et al. (2002)	*J Periodontol*	Adjunctive benefits of subantimicrobial dose doxycycline in the management of severe, generalized, chronic periodontitis.	Discontinued adjunct (SDD 20mg)
41.	Novak et al. (2008)	*J Periodontol*	Combining host modulation and topical antimicrobial therapy in the management of moderate to severe periodontitis: a randomized multicenter trial.	Discontinued adjunct (SDD 20mg)
42.	Pârvu et al. (2013)	*Acta Pharmacol Sin*	Efficacy of subantimicrobial-dose doxycycline against nitrosative stress in chronic periodontitis.	Discontinued adjunct (SDD 20mg)
43.	Pradeep et al. (2011)	*Arch Oral Biol*	Clarithromycin, as an adjunct to non-surgical periodontal therapy for chronic periodontitis: a double blinded, placebo controlled, randomized clinical trial.	Insufficient outcome data
44.	Pradeep et al. (2012)	*J Periodontol*	Systemic ornidazole as an adjunct to non-surgical periodontal therapy in the treatment of chronic periodontitis: a randomized, double-masked, placebo-controlled clinical trial.	Used of 0.2% CHX mouth rinse twice daily
45.	Pradeep et al. (2015)	*J Investig Clin Dent*	Clinical and microbiological effects of levofloxacin in the treatment of chronic periodontitis: a randomized, placebo-controlled clinical trial.	Used of 0.2% CHX mouth rinse twice daily during study period

No.	Authors (Year)	Journal	Title	Reason for exclusion
46.	Preshaw et al. (2004)	*J Periodontol*	Subantimicrobial dose doxycycline enhances the efficacy of scaling and root planing in chronic periodontitis: a multicenter trial.	Discontinued adjunct (SDD 20mg; Periostat)
47.	Preshaw et al. (2008)	*J Periodontol*	Modified-release subantimicrobial dose doxycycline enhances scaling and root planing in subjects with periodontal disease.	Insufficient outcome data
48.	Preus et al. (2017)	*J Clin Periodontol*	A double-masked Randomized Clinical Trial (RCT) comparing four periodontitis treatment strategies: 5-year clinical results.	Exclusion of subjects harbouring bacteria with known insensitivity or low sensitivity to metronidazole
49.	Preus et al. (2013)	*J Periodontol*	A randomized, double-masked clinical trial comparing four periodontitis treatment strategies: 1-year clinical results.	Exclusion of subjects harbouring bacteria with known insensitivity or low sensitivity to metronidazole
50.	Pururucker et al. (2001)	*J Periodontol,*	Local versus systemic adjunctive antibiotic therapy in 28 patients with generalized aggressive periodontitis.	Not a placebo controlled RCT
51.	Rooney et al. 2002)	*J Clin Periodontol*	Adjunctive effects to non-surgical periodontal therapy of systemic metronidazole and amoxycillin alone and combined. A placebo controlled study.	Insufficient outcome data
52.	Silva-Senem et al. (2013)	*J Clin Periodontol*	Clinical and microbiological effects of systemic antimicrobials combined to an anti-infective mechanical debridement for the management of aggressive periodontitis: a 12-month randomized controlled trial.	Long-term used of CHX gel and solution
53.	Smith et al. (2002)	*J Clin Periodontol*	A double-blind placebo-controlled trial of azithromycin as an adjunct to non-surgical treatment of periodontitis in adults: clinical results.	Insufficient outcome data
54.	Tüter et al. (2010)	*J Periodontol*	Effects of scaling and root planing and subantimicrobial dose doxycycline on gingival crevicular fluid levels of matrix metalloproteinase-8, -13 and serum levels of HsCRP in patients with chronic periodontitis.	Discontinued adjunct (SDD 20mg; Periostat)
55.	Varela et al. (2011)	*J Periodontol*	Systemic antimicrobials adjunctive to a repeated mechanical and antiseptic therapy for aggressive periodontitis: a 6-month randomized controlled trial.	Long-term used of CHX gel and solution
56.	Vergani et al. (2004)	*Braz Oral Res*	Systemic use of metronidazole in the treatment of chronic periodontitis: a pilot study using clinical, microbiological, and enzymatic evaluation.	Insufficient outcome data
57.	Yek et al. (2010)	*J Periodontol*	Efficacy of amoxicillin and metronidazole combination for the management of generalized aggressive periodontitis.	Insufficient outcome data

Table S3. General characteristics of included studies according to provided adjuncts

Author (Year)	Country	Funding	Centre	Trial design	Population	Criteria	Age mean ± SD (range)		Gender (M/F)		Smokers (%)	
							Test	Control	Test	Control	Test	Control
**Amoxicillin + Metronidazole**												
Ardila et al. (2020)	Colombia	University	Single	Parallel	Generalised AgP	≥ 6 permanent teeth with at least 1 site each with PD and CAL ≥5 mm<br>≥ 6 teeth (exclude 1st molars and incisors) with at least 1 site each with PD and CAL ≥5 mm	26.1 ± 3.9	27.1± 2.9	5/7	4/8	NS	NS
Liaw et al. (2019)	Australia	University	Single	Parallel	Generalised moderate to severe ChP	> 30% of teeth with PPD ≥5 mm, CAL ≥3 mm and BOP	45 (40 – 59)	55 (49.5 – 58.5)	4/9	7/6	NS	NS
Borges et al. (2017) *	Brazil	Government	Single	Parallel	Generalised ChP	≥ 30% of the sites with PD and CAL ≥4 mm and BOP<br>≥ 6 teeth with at least one site each with PD and CAL ≥ 5 mm	46.6 ± 8.9<br>45.9 ± 7.8<br>47.0 ± 8.6<br>48.5 ± 7.4	45.6 ± 8.0	8/14<br>10/12<br>11/11<br>10/12	9/13	NS	NS
Dukić et al. (2016)	Serbia	Private	Single	Parallel	Progressed form of periodontitis	PD ≥ 5 mm + BOP	49.3		55/35		NR	NR
Saleh et al. (2016)	Australia	Private	Single	Parallel	Moderate to advanced periodontitis	≥ 8 sites with PPD ≥5mm, CAL ≥5mm BOP	52.1 ± 12.2	56.1 ± 12.8	4/9	5/7	NS	NS
Taiete et al. (2016)	Brazil	Government	Single	Parallel	Generalised AgP	≥ 8 teeth with PPD ≥ 5 mm and BoP (having at least two with PPD ≥ 7 mm)	27.5 ± 5.5	28.5 ± 5.1	6/12	6/15	NS	NS
Harks et al. (2015)	Germany	Government	Multi	Parallel	Moderate to Severe ChP and AgP	CAL >3 mm and PPDs of ≥ 6 mm at a minimum of 4 teeth	52.6 ± 10.4	50.5 ± 10.5	149/102	154/101	29.6	25.5
Silva et al. (2011)	Brazil	Government	Single	Parallel	Generalised ChP	≥ 6 teeth with at least one site each with PD and CAL ≥5 mm<br>≥ 30% of the sites with PD and CAL≥4 mm and BOP	41.2 ± 6.1	45.5 ± 9.6	8/9	7/10	NS	NS
Buchmann et al. (2010)	Germany	Private	Single	Parallel	AgP	PD ≥5 mm at 8 sites or within each quadrant	42.4 ± 7.5		NR	NR	NS	NS

Author (Year)	Country	Funding	Centre	Trial design	Population	Criteria	Age mean ± SD (range)		Gender (M/F)		Smokers (%)	
							Test	Control	Test	Control	Test	Control
Ribeiro et al. (2009)	Brazil	Government	Single	Parallel	Severe ChP	≥ 8 teeth with PD ≥5 mm and BOP ≥ 2 of the 8 teeth had PD ≥7 mm, and in 2 additional teeth the pockets had PD ≥ 6 mm	46 (34 – 55)	46.2 (30 – 66)	4/9	3/9	NS	NS
Moeintaghavi et al. (2007)	Iran	NR	Single	Parallel	Severe ChP	≥1 site in at least 3 of the 4 quadrants with a PPD ≥5 mm and CAL of >3mm, showing BOP	34.0 ± 7.8	35.0 ± 9.0	12/16	9/13	NS	NS
Xajigeorgiou et al. (2006)	Greece	NR	Single	Parallel	Generalised AgP	>30% of sites with CAL≥ 1mm	38.8 ± 8.7	37 ± 5.6	5/5	6/5	30	36.4
Winkel et al. (2001)	Netherlands	NR	Multi	Parallel	Periodontitis	≥1 site in at least 3 of the 4 quadrants with a PPD of ≥6 mm and CAL ≥3 mm, showing BOP	45 (32 – 63)	40 (28 – 55)	11/12	10/16	60.9	69.2
**Azithromycin**												
Morales et al. (2021)	Chile	Government	Single	Parallel	ChP	≥ 5 teeth with periodontal sites with PPD≥5 mm and CAL ≥3 mm, BOP≥20%	49.0 ± 7.9	52.8 ± 7.5	10/5	8/7	18.7	40
Povšič et al. (2021)	Slovenia	Government	Single	Parallel	Stage III or Stage IV periodontitis	Interdental CAL measuring ≥ 5 mm	44.0 ± 8.5	45.5 ± 10.5	12/7	14/5	21.1	26.3
Čuk et al. (2020)	Slovenia	Government	Single	Parallel	Moderate to advanced periodontitis	PD ≥ 5 mm at a minimum of 4 teeth in 4 different quadrants	44.0 ± 8.5	45.4 ± 10.5	12/7	14/5	21.1	26.3
Liaw et al. (2019)	Australia	University	Single	Parallel	Generalised moderate to severe ChP	> 30% of teeth with PPD ≥5 mm, CAL ≥3 mm and BOP	58.5 (52 – 63.3)	55 (49.5 – 58.5)	4/8	7/6	NS	NS
Saleh et al. (2016)	Australia	Private	Single	Parallel	Moderate to advanced periodontitis	≥ 8 sites with PPD ≥5mm, CAL ≥5mm BOP	56.3 ± 9.9	56.1 ± 12.8	4/8	5/7	NS	NS
Fonseca et al. (2015)	Brazil	Government	Single	Parallel	Mild to moderate ChP	≤ 5 mm CAL	44.6 ± 8.8 (35 – 60)		33/52		15.3	
Emingil et al. (2012)	Turkey	University	Single	Parallel	Generalised AgP	CAL of ≥ 5mm and PPD of ≥ 6mm on ≥ 8 teeth (at least 3 of which were other than central incisors or first molars)	28.8 ± 4.4	29.6 ± 5.9	9/7	8/8	43.8	38.5
Han et al. (2012)	Turkey	University	Single	Parallel	Generalised ChP	>30 sites with ≥5 mm CAL ≥2 sites with a PD ≥6 mm + BOP in each quadrant	46.8 ± 5.1	44.8 ± 5.0	10/4	8/6	42.8	50.0

Author (Year)	Country	Funding	Centre	Trial design	Population	Criteria	Age mean ± SD (range)		Gender (M/F)		Smokers (%)	
							Test	Control	Test	Control	Test	Control
Sampaio et al. (2011)	Brazil	Government	Single	Parallel	Generalised ChP	≥ 30% of the sites with PD and CAL ≥5 mm and BOP<br>≥ 3 non-contiguous inter-proximal sites with PD and CAL ≥7 mm and 2 other non-contiguous inter-proximal sites with PD and CAL ≥6 mm1f	44.4 ± 7.4	43.5 ± 5.9	13/7	11/9	25	25
Haas et al. (2008)	Brazil	Government	Single	Parallel	AgP	PPD and CAL of ≥4 mm + BOP in at least one incisor and one first molar	22.5 ± 3.6	20.1 ± 3.6	5/7	8/4	25.0	17.0
Gomi et al. (2007)	Japan	NR	Single	Parallel	Severe ChP	average PD of >4 mm with BOP	45.4 ± 14.3	51.0 ± 8.8	8/9	8/9	NS	NS
**Cefixime**												
Dukić et al. (2016)	Serbia	Private	Single	Parallel	Progressed form of periodontitis	PD ≥ 5 mm + BOP	49.3		55/35		NR	NR
**Clarithromycin**												
Bechara Andere et al. (2018)	Brazil	Government	Single	Parallel	Generalised AgP	PD and CAL ≥ 5 mm and with concomitant BoP	33.11 ± 4.26	31.26 ± 4.73	1/17	1/17	NS	NS
Suryaprasanna et al. (2018)	India	Nil	Single	Parallel	ChP	PD >5 mm, CAL > 3mm	(30 – 50)		NR	NR	NS	NS
Andere et al. (2017)	Brazil	Government	Single	Parallel	Generalised AgP	≥ 6 sites with PPD and CAL ≥5 mm + BOP and at least 2 sites with PD ≥7 mm	31.4 ± 3.7	31.3 ± 4.6	1/19	1/19	NS	NS
**Clindamycin**												
Sigusch et al. (2001)	Germany	NR	Single	Parallel	Generalised Periodontitis	an average of 16 sites with PD >8 mm	32.4 (NR)		20/28		NS	NS
**Doxycycline**												
Xajigeorgiou et al. (2006)	Greece	NR	Single	Parallel	Generalised AgP	>30% of sites with CAL≥ 1mm	38.5 ± 4.7	37 ± 5.6	4/6	6/5	30	36.4
Sigusch et al. (2001)	Germany	NR	Single	Parallel	Generalised Peridontitis	an average of 16 sites with PD >8 mm	32.4 (NR)		20/28		NS	NS
**Metronidazole**												
Silva et al. (2011)	Brazil	Government	Single	Parallel	Generalised ChP	≥ 6 teeth with at least one site each with PD and CAL ≥5 mm<br>≥ 30% of the sites with PD and CAL≥4 mm and BOP	48.9 ± 6.1		41.2 ± 9.6		8/9	7/10

Author (Year)	Country	Funding	Centre	Trial design	Population	Criteria	Age mean ± SD (range)		Gender (M/F)		Smokers (%)	
							Test	Control	Test	Control	Test	Control
Xajigeorgiou et al. (2006)	Greece	NR	Single	Parallel	Generalised AgP	>30% of sites with CAL≥ 1mm	40.9 ± 4.6	37 ± 5.6	7/5	6/5	41.7	36.4
Sigusch et al. (2001)	Germany	NR	Single	Parallel	Generalised Periodontitis	an average of 16 sites with PD >8 mm and intrabony lesions on at least 1 to 5 teeth over 2/3 of the root length	32.4 (NR)		20/28		NS	NS
**Moxifloxacin**												
Ardila et al. (2020)	Colombia	University	Single	Parallel	Generalised AgP	≥ 6 permanent teeth with at least 1 site each with PD and CAL ≥5 mm<br>≥ 6 teeth (exclude 1st molars and incisors) with at least 1 site each with PD and CAL ≥5 mm	25.7 ± 4.3	27.1± 2.9	4/8	4/8	NS	NS
Ardila et al. (2015)	Colombia	University	Single	Parallel	Generalised AgP	≥ 1 site each with PD and CAL ≥5 mm<br>≥ 6 teeth (exclude 1st molars and incisors) with at least 1 site each with PD and CAL ≥5 mm	28.4 ± 0.9	26.4 ± 1.1	9/11	8/12	NS	NS
**Minocycline HCl**												
Basegmez et al. (2011)	Turkey	NR	Single	Parallel	ChP	≥ 16 interproximal sites with PD ≥4 mm	39.8 ± 5.84	42.1 ± 9	20/20		NS	NS
**Satranidazole**												
Pradeep et al. (2013)	India	Commercial	Single	Parallel	ChP	PD ≥ 5mm and/or CAL ≥ 4mm	(30 – 50)		33/27		NS	NS
**Secnidazole**												
Li et al. (2017)	China	NR	Single	Parallel	ChP	≥ 6 teeth including incisors and/or first molars with at least 1 site each with PPD and CAL ≥ 5 mm<br>≥ 6 teeth (exclude than first molars and incisors) with at least 1 site each with PPD and CAL ≥ 5 mm	52		NR		NR	

AgP: Aggressive Periodontitis; ChP: Chronic Periodontitis; NS: Non-smoker's study; NR: Not reported

*Include more than 1 arm of AMX+MET with different dosages

Table S4. Intervention characteristics of included studies according to provided adjuncts

Author (Year)	Delivery of NSPT				Test group		Control	Provider
	OHI	SRP	No. of Session	Duration for Completion	Dosage	Antibiotic initiation		
**Amoxycillin + Metronidazole**								
Ardila et al. (2020)	Yes	OSFM	1	NI	500 mg + 500 mg tds for 7 days	Before SRP	Placebo	Clinician
Liaw et al. (2019)	Yes	HM	2	7 days (90 mins each)	500 mg + 400 mg tds for 14 days	After 1st SRP	SRP alone	Clinician
Borges et al. (2017)	Yes	FM	NI	2 weeks	500 mg + 250 mg tds for 7 days	After SRP	Placebo	Periodontists
					500 mg + 250 mg tds for 14 days			
					500 mg + 400 mg tds for 7 days			
					500 mg + 400 mg tds for 14 days			
Dukić et al. (2016)	NI	NI	NI	NI	500 mg + 400 mg tds for 7 days	After SRP	SRP alone	NI
Saleh et al. (2016)	Yes	FM	2 – 4	NI	500 mg + 200 mg tds for 7 days	NI	Placebo	Clinician
Taiete et al. (2016)	Yes	NI	NI	NI	375 mg + 250 mg tds for 7 days	After SRP	Placebo	NI
Harks et al. (2015)	Yes	FM	2	NI	500 mg + 400 mg tds for 7 days	After SRP	Placebo	Dentist/Dental hygienist
Silva et al. (2011)	Yes	FM	4 – 6	19 – 21 days (1 hour each)	500 mg + 400 mg tds for 14 days	After 1st SRP	Placebo	Periodontists
Buchmann et al. (2010)	NI	NI	NI	NI	500 mg + 250 mg tds for 7 days	NI	Placebo	Clinician
Ribeiro et al. (2009)	Yes	OSFM	1	45 mins	375 mg + 250 mg tds for 7 days	After SRP	Placebo	Clinician
Moeintaghavi et al. (2007)	Yes	OSFM	1	1 hour	500 mg + 250 mg tds for 7 days	After SRP	Placebo	NI
Xajigeorgiou et al. (2006)	Yes	FM	4	NI	500 mg + 500 mg tds for 7 days	After SRP	SRP alone	NI
Winkel et al. (2001)	Yes	FM	3 – 6	NI (1 hour each)	375 mg + 250 mg tds for 7 days	After SRP	Placebo	NI

Author (Year)	Delivery of NSPT				Test group		Control	Provider
	OHI	SRP	No. of Session	Duration for Completion	Dosage	Antibiotic initiation		
**Azithromycin**								
Morales et al. (2021)	Yes	QSRP	4 – 6	NI	500 mg qid for 5 days	After SRP	Placebo	Clinician
Povšič et al. (2021)	Yes	FM	2	7 days (1.5 hours each)	500 mg od for 3 days	After SRP	Placebo	Clinician
Čuk et al. (2020)	Yes	Sites with PD ≥5 mm	2	1.5 hours per session	500 mg od for 3 days	After SRP	Placebo	Clinician
Liaw et al. (2019)	Yes	HM	2	7 days (90 mins each)	500 mg od for 3 days	After 1st SRP	SRP alone	Clinician
Saleh et al. (2016)	Yes	FM	2 – 4	NI	500 mg od for 3 days	NI	Placebo	Clinician
Fonseca et al. (2015)	NI	FM	2	24 hours (60 mins each)	500 mg od for 3 days	After 1st SRP	SRP alone	Periodontists
	NI	QSRP	4	30 mins each in weekly interval	500 mg od for 3 days	After 1st SRP	QSRP alone	Periodontists
Emingil et al. (2012)	Yes	QSRP	4	NI	500 mg od for 3 days	After SRP	Placebo	NI
Han et al. (2012)	Yes	QSRP	4	NI	500 mg od for 3 days	After SRP	Placebo	Clinician
Sampaio et al. (2011)	Yes	FM	4 – 6	2 weeks (2 hours each)	500 mg od for 5 days	After SRP	Placebo	Periodontists
Haas et al. (2008)	Yes	QSRP/SSRP	4 – 6	14 days	500 mg od for 3 days	Before SRP	Placebo	Clinician
Gomi et al. (2007)	Yes	OSFM	1	90 mins	500 mg od for 3 days	Before SRP	SRP alone	Clinician
**Cefixime**								
Dukić et al. (2016)	NI	NI	NI	NI	400 mg od for 7 days	After SRP	SRP alone	NI
**Clarithromycin**								
Bechara Andere et al. (2018)	Yes	OSFM	1	NI	500 mg bd for 3 days	After SRP	SRP alone	Clinician
Suryaprasanna et al. (2018)	NI	NI	NI	NI	500 mg tds for 7 days	Along with SRP	SRP alone	NI
Andere et al. (2017)	Yes	OSFM	1	≤1 hour	500 mg bd for 3 days	Before SRP	Placebo	Clinician
**Clindamycin**								
Sigusch et al. (2001)	Yes	FM	1 – 2	2 days (2 hours each)	150 mg qid for 8 days	After SRP	SRP alone	Dental hygienists
**Doxycycline**								
Xajigeorgiou et al. (2006)	Yes	FM	4	NI	200 mg then 100 mg od for 14 days	After SRP	SRP alone	NI
Sigusch et al. (2001)	Yes	FM	1 – 2	2 days (2 hours each)	200 mg od for 8 days	After SRP	SRP alone	Dental hygienists

Author (Year)	Delivery of NSPT				Test group		Control	Provider
	OHI	SRP	No. of Session	Duration for Completion	Dosage	Antibiotic initiation		
**Metronidazole**								
Silva et al. (2011)	Yes	FM	4 – 6	19 – 21 days (1 hour each)	400 mg tds for 14 days	After 1st SRP	Placebo	Periodontists
Xajigeorgiou et al. (2006)	Yes	FM	4	NI	500 mg tds for 7 days	After SRP	SRP alone	NI
Sigusch et al. (2001)	Yes	FM	1 – 2	2 days (2 hours each)	500 mg bd for 8 days	After SRP	SRP alone	Dental hygienists
**Moxifloxacin**								
Ardila et al. (2020)	Yes	OSFM	1	NI	400 mg qid for 7 days	Before SRP	Placebo	Clinician
Ardila et al. (2015)	Yes	OSFM	1	2.5 hours	400 mg od for 7 days	Before SRP	Placebo	Clinician
**Satranidazole**								
Pradeep et al. (2013)	Yes	NI	NI	NI	300 mg bd for 10 days	After SRP	Placebo	NI
**Secnidazole**								
Li et al. (2017)	NI	FM	NI	NI	1 g od for 30 days	48 hours after SRP	Placebo	NI

FM: Full mouth; HM: Half-mouth; NI: No information; OSFM: One-staged full mouth; QSRP: Per-quadrant Scaling & Root Planing; SSRP: Per-sextant Scaling & Root Planing

Table S5. Reported outcomes and the outcome measurements of included studies

Author (Year)	No. of Examiners	Calibration	Blinding	Probing type	Use of stent	Reported outcome of interest and outcomes measurement			
						CAL	PPD	BOP	AE
Morales et al. (2021)	1	NI	Yes	Manual	No	6 sites per tooth	6 sites per tooth	NI	Self-report
Povšič et al. (2021)	1	Yes	Yes	Manual	No	6 sites per tooth, PD + REC	6 sites per tooth	Absence/presence of bleeding after probing	Self-report
Ardila et al. (2020)	1	Yes	Yes	Manual	No	6 sites per tooth	6 sites per tooth	NI	Self-report form
Čuk et al. (2020)	1	NR	Yes	Manual	No	6 sites per tooth, PD + REC	6 sites per tooth	NI	Self-report
Liaw et al. (2019)	1	Yes	Yes	Manual	No	6 sites per tooth, Bottom of pocket to CEJ	6 sites per tooth	NI	Self-report
Bechara Andere et al. (2018)	1	Yes	Yes	Manual	No	6 sites per tooth, Bottom of pocket to CEJ	6 sites per tooth, Bottom of pocket to GM	NI	NI

Author (Year)	No. of Examiners	Calibration	Blinding	Probing type	Use of stent	Reported outcome of interest and outcomes measurement			
						CAL	PPD	BOP	AE
Suryaprasanna et al. (2018)	NI	NI	Yes	Manual	No	NI	NI	NR	NR
Andere et al. (2017)	1	Yes	Yes	Manual	No	6 sites per tooth, Bottom of pocket to CEJ	6 sites per tooth, Bottom of pocket to GM	NI	Self-report form
Borges et al. (2017)	1	Yes	Yes	Manual	No	6 sites per tooth	6 sites per tooth	NI	Self-report form
Li et al. (2017)	1	NI	NI	Manual	No	6 sites per tooth, from CEJ to the bottom of the gingival sulcus	4 sites per tooth, bottom of the periodontal pocket to the gingival line	NR	NR
Dukić et al. (2016)	NI	NI	NI	Manual	No	Routine method (unclear)	Routine method (unclear)	Mühlemann & Son (1971)	Self-report
Sampaio et al. (2016)	2	Yes	Yes	Manual	No	6 sites per tooth, Bottom of pocket to CEJ	6 sites per tooth, Bottom of pocket to GM	Absence/presence of bleeding after probing	Self-administered questionnaire
Taiete et al. (2016)	1	Yes	Yes	Manual	Yes	bottom of the pocket to the stent margin	bottom of pocket to the GM	NR	Self-administered questionnaire
Ardila et al. (2015)	1	Yes	Yes	Manual	No	6 sites per tooth, PD + (CEJ to GM)	6 sites per tooth	NI	Self-perceived questionnaire
Fonseca et al. (2015)	2	Yes	Yes	Manual	No	4 sites per tooth	4 sites per tooth	NR	NR
Harks et al. (2015)	>1 (NI)	NI	Yes	Manual & Electronic pressure-sensitive	No	6 sites per tooth, distance from occlusal surface to the bottom of the periodontal pocket	6 sites per tooth	NR	Self-report
Pradeep et al. (2013)	1	Yes	Yes	Manual	No	6 sites per tooth, CEJ to deepest probable point	6 sites per tooth, GM to bottom of pocket	NR	Self-report
Emingil et al. (2012)	1	Yes	Yes	Manual	No	6 sites per tooth, bottom of pocket to CEJ or anatomical landmark if CEJ not visible	6 sites per tooth	NI	Self-report
Han et al. (2012)	1	Yes	Yes	Manual	No	6 sites per tooth, bottom of pocket to CEJ or	6 sites per tooth	NR	Self-report

Author (Year)	No. of Examiners	Calibration	Blinding	Probing type	Use of stent	Reported outcome of interest and outcomes measurement			
						CAL	PPD	BOP	AE
						anatomical landmark if CEJ not visible			
Basegmez et al. (2011)	1	NI	Yes	Manual	Yes	NR	NR	NI	NR
Sigusch et al. (2011)	2	No	Yes	Manual	No	6 sites per tooth, Bottom of pocket to CEJ	6 sites per tooth	NR	NR
Silva et al. (2011)	2	Yes	Yes	Manual	No	6 sites per tooth, Bottom of pocket to CEJ	6 sites per tooth, Bottom of pocket to GM	NI	Self-perceived questionnaire
Buchmann et al. (2010)	1	Yes	Yes	NI	No	6 sites per tooth	6 sites per tooth	NI	NR
Ribeiro et al. (2009)	1	Yes	Yes	Manual	Yes	6 sites per tooth, from stent to bottom of pocket	6 sites per tooth, from stent to GM	Absence/presence of bleeding after probing	Self-administered questionnaire
Saleh et al. (2009)	1	Yes	Yes	Manual	No	6 sites per tooth	6 sites per tooth	Absence/presence of bleeding after probing	NI
Xajigeorgiou et al. (2006)	1	NI	Yes	Manual	No	6 sites per tooth	6 sites per tooth	NR	Self-report
Haas et al. (2008)	1	Yes	Yes	Manual	No	6 sites per tooth, PD + REC	6 sites per tooth	present/absent after PPD measurements	Self-report
Gomi et al. (2007)	NI	NI	NI	NI	NI	NI	NI	NI	NI
Moeintaghavi et al. (2007)	NI	NI	Yes	Manual	No	4 sites per tooth, bottom of pocket to CEJ	4 sites per tooth	Presence/absent 30 seconds after probing	Self-report
Winkel et al. (2001)	2	NI	NI	Manual	No	6 sites per tooth, Bottom of pocket to CEJ	6 sites per tooth	NR	NI

AE: Adverse events; BOP: Bleeding on probing; CAL: Clinical attachment level; CEJ: cemento-enamel junction; GM: Gingival margin; PPD: Probable pocket depth; NI: No information; NR: Not reported; REC: gingival recession

Table S6. Summary of CAL outcome reported in included studies

Dosage	Author (Year)	Follow-up	Intervention				Control				Notes
			Sample size (BL/End)	Baseline (Mean ± SD)	Follow-up (Mean ± SD)	CAL gain (Mean ± SD)	Sample size (BL/End)	Baseline (Mean ± SD)	Follow-up (Mean ± SD)	CAL gain (Mean ± SD)	
**Amoxycillin + Metronidazole**											
500mg + 500mg tds 7 days	Xajigeorgiou et al. (2006)	6 weeks	12/10	4.97 ± 1.01	4.31 ± 0.92	0.66 ± 0.43	12/11	4.55 ± 0.72	3.80 ± 0.62	0.75 ± 0.29	MD ± SD not reported, PP
	Ardila et al. (2020)	3 mths.	12/11	4.75 ± 0.80	3.27 ± 0.40	1.48 ± 0.26	12/11	4.74 ± 0.70	4.02 ± 0.20	0.72 ± 0.21	MD ± SD not reported, ITT
	Ardila et al. (2020)	6 mths.	12/11	4.75 ± 0.80	3.09 ± 0.30	1.66 ± 0.25	12/11	4.74 ± 0.70	3.99 ± 0.30	0.75 ± 0.23	
	Xajigeorgiou et al. (2006)		12/10	4.97 ± 1.01	4.05 ± 1.34	0.92 ± 0.53	12/11	4.55 ± 0.72	4.07 ± 0.59	0.48 ± 0.28	MD ± SD not reported, PP
500mg + 400mg tds 7 days	Dukić et al. (2016)	7 days	30/30	3.58 ± 1.13	3.20 ± 1.01	0.38 ± 0.28	30/30	3.61 ± 0.92	3.39 ± 1.17	0.22 ± 0.27	MD ± SD not reported, ITT
	Borges et al. (2017)	3 mths.	22/20	4.70 ± 1.30	4.20 ± 1.30	0.20 ± 0.32	22/19	4.40 ± 1.00	4.10 ± 1.10	0.30 ± 0.34	
		6 mths.	22/20	4.70 ± 1.30	4.10 ± 1.40	0.30 ± 0.30	22/19	4.40 ± 1.00	4.20 ± 1.10	0.20 ± 0.34	
		12 mths.	22/20	4.70 ± 1.30	4.00 ± 1.40	0.70 ± 0.41	22/19	4.40 ± 1.00	4.10 ± 1.00	0.30 ± 0.32	
500mg + 250mg tds 7 days	Borges et al. (2017)	3 mths.	22/21	4.50 ± 0.90	3.90 ± 1.00	0.60 ± 0.29	22/19	4.40 ± 1.00	4.10 ± 1.10	0.30 ± 0.34	MD ± SD not reported, ITT
		6 mths.	22/21	4.50 ± 0.90	3.90 ± 1.00	0.60 ± 0.29	22/19	4.40 ± 1.00	4.20 ± 1.10	0.20 ± 0.34	
		12 mths.	22/21	4.50 ± 0.90	3.70 ± 1.30	0.80 ± 0.34	22/19	4.40 ± 1.00	4.10 ± 1.00	0.30 ± 0.32	
	Harks et al. (2015)	27.5 mths.	251/170	4.10 ± 0.90	3.40 ± 0.90	0.70 ± 0.09	255/175	4.10 ± 1.00	3.70 ± 1.00	0.40 ± 0.11	MD ± SD not reported, RAL, ITT
500mg + 200mg tds 7 days	Buchmann et al. (2010)	3 weeks	36/36	6.70 ± 1.50	4.60 ± 1.20	2.10 ± 0.70	32/32	5.90 ± 1.00	4.60 ± 0.90	1.30 ± 0.50	
	Saleh et al. (2016)	3 mths.	13/11	4.51 ± 0.82	3.97 ± 0.82	0.55 ± 0.65	12/10	4.38 ± 0.61	4.08 ± 0.69	0.3 ± 0.35	PP
500mg + 400mg tds 14 days	Liaw et al. (2019)	2 mths.	13/12	4.21 ± 1.10	3.67 ± 1.51	0.54 ± 0.53	13/11	5.01 ± 1.19	4.35 ± 0.68	0.66 ± 0.39	Median (IQR) converted to mean (SD), PP
	Borges et al. (2017)	3 mths.	22/20	4.80 ± 1.20	4.10 ± 1.10	0.70 ± 0.35	22/19	4.40 ± 1.00	4.10 ± 1.10	0.30 ± 0.32	MD ± SD not reported, ITT

Dosage	Author (Year)	Follow-up	Intervention Sample size (BL/End)	Intervention Baseline (Mean ± SD)	Intervention Follow-up (Mean ± SD)	Intervention CAL gain (Mean ± SD)	Control Sample size (BL/End)	Control Baseline (Mean ± SD)	Control Follow-up (Mean ± SD)	Control CAL gain (Mean ± SD)	Notes
	Silva et al. (2011)		17/17	4.40 ± 0.60	3.60 ± 0.70	1.37 ± 0.39	17/17	4.20 ± 0.70	3.70 ± 0.70	1.53 ± 0.27	ITT
	Borges et al. (2017)	6 mths.	22/20	4.80 ± 1.20	4.20 ± 1.20	0.60 ± 0.36	22/19	4.40 ± 1.00	4.20 ± 1.10	0.20 ± 0.32	MD ± SD not reported, ITT
		12 mths.	22/20	4.80 ± 1.20	4.00 ± 1.10	0.80 ± 0.35	22/19	4.40 ± 1.00	4.10 ± 1.00	0.30 ± 0.30	
500mg + 250mg tds 14 days	Borges et al. (2017)	3 mths.	22/20	4.20 ± 0.80	3.50 ± 0.80	0.70 ± 0.24	22/19	4.40 ± 1.00	4.10 ± 1.10	0.30 ± 0.32	MD ± SD not reported, ITT
		6 mths.	22/20	4.20 ± 0.80	3.50 ± 0.80	0.70 ± 0.24	22/19	4.40 ± 1.00	4.20 ± 1.10	0.20 ± 0.32	
		12 mths.	22/20	4.20 ± 0.80	3.40 ± 0.70	0.80 ± 0.23	22/19	4.40 ± 1.00	4.10 ± 1.00	0.30 ± 0.30	
375mg + 250mg tds 7 days	Taiete et al. (2016)	3 mths.	24/21	8.60 ± 1.40	1.90 ± 0.60	6.70 ± 0.33	24/18	8.60 ± 1.00	1.40 ± 0.80	7.20 ± 0.30	MD ± SD not reported, rCAL, PP
	Ribeiro et al. (2009)	6 mths.	-/13	8.15 ± 0.55	6.27 ± 3.16	1.88 ± 0.89	-/12	8.22 ± 1.02	6.54 ± 3.13	1.68 ± 0.95	Follow-up not reported, RAL, PP
	Taiete et al. (2016)		24/21	8.60 ± 1.40	1.70 ± 0.90	6.90 ± 0.36	24/18	8.60 ± 1.00	1.50 ± 0.90	7.10 ± 0.32	MD ± SD not reported, rCAL, P
	Winkel et al. (2001)		-/23	3.90 ± 1.10	3.20 ± 1.00	0.70 ± 0.31	-/26	4.00 ± 1.30	3.60 ± 1.10	0.40 ± 0.33	
**Azithromycin**											
500mg od 3 days	Han et al. (2012)	1 mth.	18/14	5.68 ± 0.70	4.21 ± 0.88	1.47 ± 0.30	18/14	5.32 ± 1.00	3.96 ± 1.58	1.36 ± 0.50	Follow-up not reported, PP
	Emingil et al. (2012		18/16	5.82 ± 0.92	4.18 ± 0.73	1.64 ± 0.29	18/16	5.25 ± 0.60	3.78 ± 0.45	1.47 ± 0.18	95% CI converted to mean (SD), PP
	Liaw et al. (2019)	2 mths.	12/11	4.31 ± 0.59	3.54 ± 0.42	0.77 ± 0.22	13/11	5.01 ± 1.19	4.35 ± 0.68	0.66 ± 0.41	Median (IQR) converted to mean (SD), PP
	Emingil et al. (2012)	3 mths.	18/16	5.82 ± 0.92	3.99 ± 0.66	1.83 ± 0.28	18/16	5.25 ± 0.60	3.66 ± 0.48	1.59 ± 0.19	95% CI converted to mean (SD), PP
	Gomi et al. (2007)		17/17	7.47 ± 1.96	4.71 ± 1.12	2.76 ± 0.55	17/17	7.21 ± 1.37	5.35 ± 0.88	1.86 ± 0.39	MD ± SD not reported, ITT
	Han et al. (2012)		18/14	5.68 ± 0.70	4.10 ± 1.32	1.58 ± 0.40	18/14	5.32 ± 1.00	3.84 ± 2.01	1.48 ± 0.60	Follow-up not reported, PP
	Saleh et al. (2016)		12/11	3.96 ± 0.58	3.81 ± 0.61	0.15 ± 0.25	12/10	4.38 ± 0.61	4.08 ± 0.69	0.30 ± 0.35	PP

Dosage	Author (Year)	Follow-up	Intervention				Control				Notes
			Sample size (BL/End)	Baseline (Mean ± SD)	Follow-up (Mean ± SD)	CAL gain (Mean ± SD)	Sample size (BL/End)	Baseline (Mean ± SD)	Follow-up (Mean ± SD)	CAL gain (Mean ± SD)	
	Fonseca et al. (2015)		15/15	2.73 ± 1.15	2.68 ± 1.14	0.05 ± 0.42	15/15	2.39 ± 0.99	2.17 ± 0.77	0.22 ± 0.32	MD ± SD not reported, FMD, PP
			15/13	2.38 ± 0.79	2.27 ± 0.71	0.11 ± 0.29	15/14	2.60 ± 1.19	2.46 ± 1.11	0.14 ± 0.43	MD ± SD not reported, QSRP, PP
	Čuk et al. (2020)	6 mths.	20/19	4.24 ± 0.88	3.23 ± 0.80	1.00 ± 0.48	20/19	4.24 ± 1.04	3.41 ± 1.36	0.81 ± 0.57	Median (IQR) converted to mean (SD), PP
	Emingil et al. (2012)		18/16	5.82 ± 0.92	3.93 ± 0.68	1.89 ± 0.29	18/16	5.25 ± 0.60	3.61 ± 0.48	1.64 ± 0.19	95% CI converted to mean (SD), PP
	Gomi et al. (2007)		17/17	7.47 ± 1.96	4.85 ± 1.05	2.62 ± 0.54	17/17	7.21 ± 1.37	5.74 ± 0.96	1.47 ± 0.41	MD ± SD not reported, ITT
	Han et al. (2012)		18/14	5.68 ± 0.70	4.13 ± 1.73	1.55 ± 0.50	18/14	5.32 ± 1.00	3.78 ± 1.58	1.54 ± 0.50	Follow-up not reported, PP
	Fonseca et al. (2015)		15/15	2.73 ± 1.15	2.61 ± 1.15	0.12 ± 0.42	15/15	2.39 ± 0.99	2.20 ± 0.74	0.19 ± 0.32	MD ± SD not reported, FMD, PP
			15/13	2.38 ± 0.79	2.25 ± 0.72	0.13 ± 0.30	15/14	2.60 ± 1.19	2.41 ± 1.04	0.19 ± 0.42	MD ± SD not reported, QSRP, PP
	Haas et al. (2008)	12 mths.	12/12	5.90 ± 1.80	4.22 ± 1.57	1.68 ± 0.69	13/12	5.70 ± 1.80	4.73 ± 2.96	0.97 ± 1.00	Follow-up not reported, MD (SE) converted to MD (SD), PP
	Povšič et al. (2021)		20/19	4.24 ± 0.88	3.78 ± 1.52	0.46 ± 0.40	20/19	4.24 ± 1.04	3.52 ± 1.2	0.72 ± 0.36	Median (IQR) converted to mean (SD), PP
500mg od 5 days	Morales et al. (2021)	3 mths.	16/16	4.40 ± 0.90	3.80 ± 0.80	0.40 ± 0.30	15/15	4.70 ± 1.50	4.10 ± 1.40	0.60 ± 0.40	ITT
	Morales et al. (2021)	6 mths.	16/16	4.40 ± 0.90	4.00 ± 1.00	0.40 ± 0.40	15/15	4.70 ± 1.50	4.20 ± 1.40	0.50 ± 0.40	
	Sampaio et al. (2011)		20/19	5.51 ± 0.94	4.43 ± 0.81	1.05 ± 1.56	20/19	5.74 ± 0.83	4.70 ± 0.83	1.05 ± 1.54	
	Morales et al. (2021)	9 mths.	16/16	4.40 ± 0.90	4.10 ± 1.00	0.30 ± 0.40	15/15	4.70 ± 1.50	4.30 ± 1.50	0.40 ± 0.40	
	Morales et al. (2021)	12 mths.	16/16	4.40 ± 0.90	4.10 ± 1.10	0.30 ± 0.40	15/15	4.70 ± 1.50	4.40 ± 1.50	0.30 ± 0.40	
	Sampaio et al. (2011)		20/19	5.51 ± 0.94	4.44 ± 0.77	1.02 ± 1.62	20/19	5.74 ± 0.83	4.69 ± 0.89	1.04 ± 1.65	
**Cefixime**											
400mg od 7 days	Dukić et al. (2016)	7 days	30/30	3.54 ± 0.99	3.20 ± 1.12	0.34 ± 0.27	30/30	3.61 ± 0.92	3.39 ± 1.17	0.22 ± 0.27	MD ± SD not reported, ITT

Dosage	Author (Year)	Follow-up	Intervention				Control				Notes
			Sample size (BL/End)	Baseline (Mean ± SD)	Follow-up (Mean ± SD)	CAL gain (Mean ± SD)	Sample size (BL/End)	Baseline (Mean ± SD)	Follow-up (Mean ± SD)	CAL gain (Mean ± SD)	
**Clindamycin**											
150mg qid 8 days	Sigusch et al. (2001)	6 mths.	11/11	6.10 ± 0.96	4.40 ± 1.00	1.70 ± 0.42	10/10	6.30 ± 0.77	5.70 ± 0.96	0.60 ± 0.39	MD ± SD not reported, ITT
		24 mths.	11/11	6.10 ± 0.96	4.20 ± 0.87	1.90 ± 0.39	10/10	6.30 ± 0.77	5.90 ± 0.76	0.40 ± 0.34	
**Clarithromycin**											
500mg bd 3 days	Andere et al. (2017)	3 mths	20/20	3.48 ± 0.66	2.65 ± 0.42	0.83 ± 0.17	20/20	3.63 ± 0.70	3.00 ± 0.50	0.63 ± 0.19	MD ± SD not reported, ITT
	Andere et al. (2017)	6 mths.	20/20	3.48 ± 0.66	2.71 ± 0.35	0.77 ± 0.40	20/20	3.63 ± 0.70	3.03 ± 0.55	0.60 ± 0.40	ITT
	Bechara Andere et al. (2018)		18/18	3.28 ± 0.69	2.65 ± 0.37	0.63 ± 0.31	18/18	3.86 ± 0.76	3.06 ± 0.55	0.80 ± 0.21	ITT
500mg tds 7 days	Suryaprasanna et al. (2018)	3 mths.	15/15	6.25 ± 0.40	3.08 ± 0.66	3.17 ± 0.20	15/15	6.25 ± 0.32	3.32 ± 0.42	2.93 ± 0.14	MD ± SD not reported, ITT
		6 mths.	15/15	6.25 ± 0.40	3.32 ± 0.42	2.93 ± 0.15	15/15	6.25 ± 0.32	3.64 ± 0.44	2.61 ± 0.14	
**Doxycycline**											
200mg od 8 days	Sigusch et al. (2001)	6 mths.	12/12	6.00 ± 1.05	4.80 ± 0.83	1.20 ± 0.39	10/10	6.30 ± 0.77	5.70 ± 0.96	0.60 ± 0.39	MD ± SD not reported, ITT
		24 mths.	12/12	6.00 ± 1.05	5.10 ± 0.90	0.90 ± 0.40	10/10	6.30 ± 0.77	5.90 ± 0.76	0.40 ± 0.34	
200mg then 100mg od 14 days	Xajigeorgiou et al. (2006)	6 weeks	12/10	5.03 ± 1.42	4.43 ± 1.73	0.60 ± 0.71	12/11	4.55 ± 0.72	3.80 ± 0.62	0.75 ± 0.29	MD ± SD not reported, PP
		6 mths.	12/10	5.03 ± 1.42	4.22 ± 1.92	0.81 ± 0.76	12/11	4.55 ± 0.72	4.07 ± 0.59	0.48 ± 0.28	
**Metronidazole**											
500mg tds 7 days	Xajigeorgiou et al. (2006)	6 weeks	12/12	5.35 ± 1.27	4.61 ± 1.13	0.74 ± 0.49	12/11	4.55 ± 0.72	3.80 ± 0.62	0.75 ± 0.29	MD ± SD not reported, PP
		6 mths.	12/12	5.35 ± 1.27	4.22 ± 1.34	1.13 ± 0.53	12/11	4.55 ± 0.72	4.07 ± 0.59	0.48 ± 0.28	
500mg bd 8 days	Sigusch et al. (2001)	6 mths.	15/15	6.20 ±1.00	4.30 ± 0.74	1.90 ± 0.32	10/10	6.30 ± 0.77	5.70 ± 0.96	0.60 ± 0.39	MD ± SD not reported, ITT
		24mths.	15/15	6.20 ± 1.00	4.00 ± 1.07	2.20 ± 0.38	10/10	6.30 ± 0.77	5.90 ± 0.76	0.40 ± 0.34	

Dosage	Author (Year)	Follow-up	Intervention				Control				Notes
			Sample size (BL/End)	Baseline (Mean ± SD)	Follow-up (Mean ± SD)	CAL gain (Mean ± SD)	Sample size (BL/End)	Baseline (Mean ± SD)	Follow-up (Mean ± SD)	CAL gain (Mean ± SD)	
400mg tds 14 days	Silva et al. (2011)	3 mths.	17/17	4.00 ± 0.70	3.40 ± 0.60	1.19 ± 0.48	17/17	4.20 ± 0.70	3.70 ± 0.70	1.53 ± 0.27	ITT
**Moxifloxacin**											
400mg od 7 days	Ardila et al. (2015)	3 mths.	20/20	4.92 ± 0.50	3.17 ± 0.60	1.75 ± 0.17	20/20	4.93 ± 0.40	3.84 ± 0.50	1.09 ± 0.14	MD ± SD not reported, ITT
		6 mths.	20/20	4.92 ± 0.50	3.14 ± 0.60	1.78 ± 0.17	20/20	4.93 ± 0.40	3.77 ± 0.40	1.16 ± 0.13	
400mg qid 7 days	Ardila et al. (2020)	3 mths.	12/12	4.82 ± 0.80	3.25 ± 0.30	1.57 ± 0.25	12/11	4.74 ± 0.70	4.02 ± 0.20	0.72 ± 0.21	
		6 mths.	12/12	4.82 ± 0.80	3.05 ± 0.20	1.77 ± 0.24	12/11	4.74 ± 0.70	3.99 ± 0.30	0.75 ± 0.22	
**Satranidazole**											
300mg bd 10 days	Pradeep et al. (2013)	1 mth.	33/30	8.42 ± 0.72	6.21 ± 1.15	2.24 ± 1.21	33/30	8.62 ± 0.85	8.12 ± 0.95	0.51 ± 1.25	PP
		3 mths.	33/30	8.42 ± 0.72	5.67 ± 1.37	2.87 ± 1.66	33/30	8.62 ± 0.85	7.85 ± 1.29	0.83 ± 1.25	PP
		6 mths.	33/30	8.42 ± 0.72	5.22 ± 1.42	3.22 ± 1.01	33/30	8.62 ± 0.85	7.51 ± 1.30	1.15 ± 1.49	PP
**Secnidazole**											
1g od 30 days	Li et al. (2017)	3 mths.	25/25	5.20 ± 1.80	4.40 ± 0.90	0.80 ± 0.40	25/25	5.00 ± 1.70	4.40 ± 0.90	0.60 ± 0.38	MD ± SD not reported, ITT
**Minocycline HCl**											
200mg then 100mg od 14 days	Basegmez et al. (2011)	1 mth.	20/20	10.08 ± 1.33	9.12 ± 1.62	0.96 ± 0.47	20/20	8.70 ± 0.99	7.83 ± 1.78	0.87 ± 0.46	Follow-up not reported, RAL, Median (Min – Max) converted to Mean (SD), ITT
		3 mths.	20/20	10.08 ± 1.33	9.10 ± 1.38	0.98 ± 0.43	20/20	8.70 ± 0.99	7.82 ± 2.15	0.88 ± 0.53	
		6 mths.	20/20	10.08 ± 1.33	9.12 ± 1.30	0.97 ± 0.42	20/20	8.70 ± 0.99	7.80 ± 1.53	0.90 ± 0.41	

Table S7. Summary of PPD outcome reported in included studies

Dosage	Author (Year)	Follow-up	Intervention				Control				Notes
			Sample size (BL/End)	Baseline (Mean ± SD)	Follow-up (Mean ± SD)	PPD reduction (Mean ± SD)	Sample size (BL/End)	Baseline (Mean ± SD)	Follow-up (Mean ± SD)	PPD reduction (Mean ± SD)	
**Amoxycillin + Metronidazole**											
500mg + 500mg tds 7 days	Xajigeorgiou et al. (2006)	6 weeks	12/10	4.63 ± 0.97	3.44 ± 0.48	1.19 ± 0.34	12/11	4.21 ± 0.74	3.19 ± 0.59	1.02 ± 0.29	MD ± SD not reported, PP
	Ardila et al. (2020)	3 mths.	12/11	4.88 ± 0.60	3.11 ± 0.30	1.77 ± 0.19	12/11	4.80 ± 0.70	4.06 ± 0.20	0.74 ± 0.21	MD ± SD not reported, ITT
	Ardila et al. (2020)	6 mths.	12/11	4.88 ± 0.60	3.07 ± 0.20	1.81 ± 0.18	12/11	4.80 ± 0.70	4.02 ± 0.30	0.78 ± 0.22	
	Xajigeorgiou et al. (2006)		12/10	4.63 ± 0.97	3.12 ± 0.71	1.51 ± 0.38	12/11	4.21 ± 0.74	3.52 ± 0.76	0.69 ± 0.32	MD ± SD not reported, PP
500mg + 400mg tds 7 days	Dukić et al. (2016)	7 days	30/30	4.27 ± 0.16	4.24 ± 0.15	0.03 ± 0.04	30/30	4.30 ± 0.18	4.30 ± 0.17	0 ± 0.05	MD ± SD not reported, ITT
	Borges et al. (2017)	3 mths.	22/20	4.00 ± 0.90	2.80 ± 0.50	1.20 ± 0.22	22/19	3.80 ± 0.70	3.00 ± 0.60	0.80 ± 0.20	
		6 mths.	22/20	4.00 ± 0.90	2.60 ± 0.50	1.40 ± 0.22	22/19	3.80 ± 0.70	3.00 ± 0.60	0.80 ± 0.20	
		12 mths.	22/20	4.00 ± 0.90	2.60 ± 0.50	1.40 ± 0.22	22/19	3.80 ± 0.70	2.90 ± 0.60	0.90 ± 0.20	
500mg + 400mg tds 14 days	Liaw et al. (2019)	2 mths.	13/12	3.64 ± 0.50	2.76 ± 0.41	0.88 ± 0.18	13/11	3.89 ± 0.76	3.39 ± 0.93	0.50 ± 0.33	Median (IQR) converted to mean (SD), PP
	Borges et al. (2017)	3 mths.	22/20	4.00 ± 0.80	2.70 ± 0.30	1.30 ± 0.18	22/19	3.80 ± 0.70	3.00 ± 0.60	0.80 ± 0.20	MD ± SD not reported, ITT
	Silva et al. (2011)		17/17	3.80 ± 0.50	2.70 ± 0.50	1.08 ± 0.40	17/17	3.60 ± 0.50	3.00 ± 0.60	0.63 ± 0.26	ITT
	Borges et al. (2017)	6 mths.	22/20	4.00 ± 0.80	2.60 ± 0.40	1.40 ± 0.19	22/19	3.80 ± 0.70	3.00 ± 0.60	0.80 ± 0.20	MD ± SD not reported, ITT
		12 mths.	22/20	4.00 ± 0.80	2.50 ± 0.40	1.50 ± 0.19	22/19	3.80 ± 0.70	2.90 ± 0.60	0.90 ± 0.20	
	Harks et al. (2015)	27.5 mths.	251/170	3.60 ± 0.70	2.40 ± 0.50	1.20 ± 0.05	255/175	3.50 ± 0.80	2.70 ± 0.70	0.80 ± 0.07	
500mg + 250mg tds 7 days	Borges et al. (2017)	3 mths.	22/21	3.80 ± 0.90	2.60 ± 0.40	1.20 ± 0.21	22/19	3.80 ± 0.70	3.00 ± 0.60	0.80 ± 0.20	
		6 mths.	22/21	3.80 ± 0.90	2.60 ± 0.40	1.20 ± 0.21	22/19	3.80 ± 0.70	3.00 ± 0.60	0.80 ± 0.20	

Dosage	Author (Year)	Follow-up	Intervention				Control				Notes
			Sample size (BL/End)	Baseline (Mean ± SD)	Follow-up (Mean ± SD)	PPD reduction (Mean ± SD)	Sample size (BL/End)	Baseline (Mean ± SD)	Follow-up (Mean ± SD)	PPD reduction (Mean ± SD)	
		12 mths.	22/21	3.80 ± 0.90	2.40 ± 0.60	1.40 ± 0.23	22/19	3.80 ± 0.70	2.90 ± 0.60	0.90 ± 0.20	
500mg + 250mg tds 14 days	Borges et al. (2017)	3 mths.	22/20	3.70 ± 0.70	2.50 ± 0.30	1.20 ± 0.16	22/19	3.80 ± 0.70	3.00 ± 0.60	0.80 ± 0.20	
		6 mths.	22/20	3.70 ± 0.70	2.40 ± 0.30	1.30 ± 0.16	22/19	3.80 ± 0.70	3.00 ± 0.60	0.80 ± 0.20	
		12 mths.	22/20	3.70 ± 0.70	2.30 ± 0.30	1.40 ± 0.16	22/19	3.80 ± 0.70	2.90 ± 0.60	0.90 ± 0.20	
500mg + 200mg tds 7 days	Buchmann et al. (2010)	3 weeks	36/36	6.00 ± 1.00	3.60 ± 0.70	2.40 ± 0.60	32/32	5.90 ± 1.00	3.70 ± 0.70	1.30 ± 0.50	
	Saleh et al. (2016)	3 mths.	13/11	3.94 ± 0.70	3.02 ± 0.21	0.92 ± 0.58	12/10	3.98 ± 0.63	3.33 ± 0.55	0.65 ± 0.42	PP
375mg + 250mg tds 7 days	Taiete et al. (2016)	3 mths.	24/21	6.50 ± 0.50	2.90 ± 0.70	3.60 ± 0.19	24/18	6.40 ± 0.40	2.20 ± 0.60	4.20 ± 0.17	MD ± SD not reported, PP
	Ribeiro et al. (2009)	6 mths.	-/13	6.40 ± 0.43	3.12 ±1.41	3.28 ± 0.41	-/12	6.17 ± 0.49	4.49 ± 3.25	1.68 ± 0.95	Follow-up not reported, PP
	Taiete et al. (2016)		24/21	6.50 ± 0.50	2.70 ± 0.80	3.80 ± 0.21	24/18	6.40 ± 0.40	2.10 ± 0.70	4.30 ± 0.19	MD ± SD not reported, PP
	Winkel et al. (2001)		-/23	4.40 ± 0.60	3.00 ± 0.40	1.40 ± 0.15	-/26	4.40 ± 0.50	3.40 ± 0.50	1.00 ± 0.14	
**Azithromycin**											
500mg od 3 days	Emingil et al. (2012)	1 mth.	18/16	4.43 ± 0.71	2.61 ± 0.36	1.82 ± 0.20	18/16	4.05 ± 0.49	2.44 ± 0.22	1.62 ± 0.13	MD ± SD not reported, 95% CI converted to mean (SD), PP
	Han et al. (2012)		18/14	4.02 ± 0.50	2.46 ± 1.41	1.56 ± 0.40	18/14	3.84 ± 0.60	2.40 ± 1.77	1.44 ± 0.50	Follow-up not reported, PP
	Liaw et al. (2019)	2 mths.	12/11	3.67 ± 0.34	2.74 ± 0.08	0.93 ± 0.11	13/11	3.89 ± 0.76	3.39 ± 0.93	0.50 ± 0.36	Median (IQR) converted to mean (SD), PP
	Emingil et al. (2012)	3 mths.	18/16	4.43 ± 0.71	2.38 ± 0.29	2.05 ± 0.19	18/16	4.05 ± 0.49	2.36 ± 0.20	1.69 ± 0.13	MD ± SD not reported, 95% CI converted to mean (SD), PP
	Gomi et al. (2007)		17/17	3.98 ± 1.06	2.21 ± 0.33	1.77 ± 0.27	17/17	4.05 ± 0.68	3.28 ± 0.41	0.77 ± 0.19	MD ± SD not reported, ITT
	Han et al. (2012)		18/14	4.02 ± 0.50	2.23 ± 1.41	1.79 ± 0.40	18/14	3.84 ± 0.60	2.30 ± 1.37	1.54 ± 0.40	Follow-up not reported, PP

Dosage	Author (Year)	Follow-up	Intervention				Control				Notes
			Sample size (BL/End)	Baseline (Mean ± SD)	Follow-up (Mean ± SD)	PPD reduction (Mean ± SD)	Sample size (BL/End)	Baseline (Mean ± SD)	Follow-up (Mean ± SD)	PPD reduction (Mean ± SD)	
	Saleh et al. (2016)		12/11	3.51 ± 0.34	3.05 ± 0.20	0.45 ± 0.30	12/10	3.98 ± 0.63	3.33 ± 0.55	0.65 ± 0.42	PP
	Fonseca et al. (2015)		15/15	2.20 ± 0.41	2.11 ± 0.41	0.09 ± 0.15	15/15	2.27 ± 0.60	2.09 ± 0.52	0.18 ± 0.21	MD ± SD not reported, FMD, PP
			15/13	2.31 ± 0.40	2.21 ± 0.40	0.10 ± 0.16	15/15	2.20 ± 0.55	1.98 ± 0.54	0.22 ± 0.21	MD ± SD not reported, QSRP, PP
	Čuk et al. (2020)	6 mths.	20/19	4.04 ± 0.64	2.74 ± 0.56	1.30 ± 0.20	20/19	4.06 ± 0.72	2.97 ± 0.96	1.09 ± 0.28	Median (IQR) converted to mean (SD), PP
	Emingil et al. (2012)		18/16	4.43 ± 0.71	2.32 ± 0.27	2.11 ± 0.19	18/16	4.05 ± 0.49	2.20 ± 0.17	1.85 ± 0.13	MD ± SD not reported, 95% CI converted to mean (SD), PP
	Gomi et al. (2007)		17/17	3.98 ± 1.06	2.36 ± 0.76	1.62 ± 0.32	17/17	4.05 ± 0.68	3.30 ± 0.36	0.75 ± 0.19	MD ± SD not reported, ITT
	Han et al. (2012)		18/14	4.02 ± 0.50	2.21 ± 1.80	1.81 ± 0.50	18/14	3.84 ± 0.60	2.18 ± 1.77	1.66 ± 0.50	Follow-up not reported, PP
	Fonseca et al. (2015)		15/15	2.20 ± 0.41	1.93 ± 0.42	0.27 ± 0.15	15/15	2.27 ± 0.60	2.08 ± 0.52	0.19 ± 0.21	MD ± SD not reported, FMD, PP
			15/13	2.31 ± 0.40	2.18 ± 0.40	0.13 ± 0.16	15/15	2.20 ± 0.55	1.93 ± 0.47	0.27 ± 0.19	MD ± SD not reported, QSRP, PP
	Haas et al. (2008)	12 mths.	12/12	6.70 ± 1.50	3.82 ± 2.33	2.88 ± 0.80	13/12	6.30 ± 1.60	4.45 ± 4.02	1.85 ± 1.25	Follow-up not reported, MD (SE) converted to MD (SD), PP
	Povšič et al. (2021)		20/19	4.04 ± 0.64	2.74 ± 0.56	1.33 ± 0.48	20/19	4.06 ± 0.72	2.77 ± 0.80	1.30 ± 0.48	PP
500mg od 5 days	Morales et al. (2021)	3 mths.	16/16	2.90 ± 0.40	2.20 ± 0.30	0.70 ± 0.50	15/15	3.20 ± 0.90	2.40 ± 0.50	0.80 ± 0.50	ITT
	Morales et al. (2021)	6 mths.	16/16	2.90 ± 0.40	2.30 ± 0.30	0.60 ± 0.30	15/15	3.20 ± 0.90	2.40 ± 0.50	0.80 ± 0.60	ITT
	Sampaio et al. (2011)		20/20	4.82 ± 0.84	3.24 ± 0.41	1.54 ± 1.62	20/20	5.02 ± 0.66	3.36 ± 0.38	1.71 ± 1.71	ITT
	Morales et al. (2021)	9 mths.	16/16	2.90 ± 0.40	2.30 ± 0.30	0.60 ± 0.30	15/15	3.20 ± 0.90	2.50 ± 0.60	0.70 ± 0.60	ITT
	Morales et al. (2021)	12 mths.	16/16	2.90 ± 0.40	2.30 ± 0.30	0.60 ± 0.40	15/15	3.20 ± 0.90	2.40 ± 0.56	0.70 ± 0.50	ITT
	Sampaio et al. (2011)		16/16	2.90 ± 0.40	2.30 ± 0.30	0.60 ± 0.30	15/15	3.20 ± 0.90	2.50 ± 0.60	0.70 ± 0.60	ITT

Dosage	Author (Year)	Follow-up	Intervention Sample size (BL/End)	Intervention Baseline (Mean ± SD)	Intervention Follow-up (Mean ± SD)	Intervention PPD reduction (Mean ± SD)	Control Sample size (BL/End)	Control Baseline (Mean ± SD)	Control Follow-up (Mean ± SD)	Control PPD reduction (Mean ± SD)	Notes
			20/19	4.82 ± 0.84	3.36 ± 0.44	1.41 ± 1.68	20/19	5.02 ± 0.66	3.34 ± 0.50	1.77 ± 1.80	ITT
**Cefixime**											
400mg od 7 days	Dukić et al. (2016)	7 days	30/30	4.31 ± 0.13	4.27 ± 0.18	0.04 ± 0.04	30/30	4.30 ± 0.18	4.30 ± 0.17	0.00 ± 0.05	MD ± SD not reported, ITT
**Clindamycin**											
150 mg qid 8 days	Sigusch et al. (2001)	6 mths.	11/11	5.70 ± 1.06	3.50 ± 0.96	2.20 ± 0.43	10/10	5.90 ± 0.70	4.60 ± 1.00	1.30 ± 0.39	MD ± SD not reported, ITT
		24 mths.	11/11	5.70 ± 1.06	3.40 ± 0.80	2.30 ± 0.40	10/10	5.90 ± 0.70	5.20 ± 0.77	0.70 ± 0.33	
**Clarithromycin**											
500mg bd 3 days	Andere et al. (2017)	3 mths	20/20	3.34 ± 0.56	2.54 ± 0.34	0.80 ± 0.15	20/20	3.53 ± 0.60	2.83 ± 0.48	0.70 ± 0.17	MD ± SD not reported, ITT
	Andere et al. (2017)	6 mths.	20/20	3.34 ± 0.56	2.53 ± 0.30	0.81 ± 0.40	20/20	3.53 ± 0.60	2.86 ± 0.52	0.67 ± 0.40	ITT
	Bechara Andere et al. (2018)		18/18	3.13 ± 0.68	2.47 ± 0.32	0.66 ± 0.26	18/18	3.76 ± 0.80	2.88 ± 0.52	0.88 ± 0.28	ITT
500mg tds 7 days	Suryaprasanna et al. (2018)	3 mths.	15/15	5.95 ± 0.67	2.07 ± 0.44	3.88 ± 0.21	15/15	5.64 ± 0.82	2.80 ± 0.49	2.84 ± 0.25	MD ± SD not reported, ITT
		6 mths.	15/15	5.95 ± 0.67	2.95 ± 0.36	3.00 ± 0.20	15/15	5.64 ± 0.82	2.91 ± 0.78	2.73 ± 0.29	
**Doxycycline**											
200mg od 8 days	Sigusch et al. (2001)	6 mths.	12/12	5.50 ± 0.62	3.90 ± 0.84	1.60 ± 0.30	10/10	5.90 ± 0.70	4.60 ± 1.00	1.30 ± 0.39	MD ± SD not reported, ITT
		24 mths.	12/12	5.50 ± 0.62	4.20 ± 1.10	1.30 ± 0.36	10/10	5.90 ± 0.70	5.20 ± 0.77	0.70 ± 0.33	
200mg then 100mg od 14 days	Xajigeorgiou et al. (2006)	6 weeks	12/10	4.24 ± 0.57	3.48 ± 0.67	0.76 ± 0.28	12/11	4.21 ± 0.74	3.19 ± 0.59	1.02 ± 0.29	MD ± SD not reported, PP
		6 mths.	12/10	4.24 ± 0.57	3.35 ± 0.76	0.89 ± 0.30	12/11	4.21 ± 0.74	3.52 ± 0.76	0.69 ± 0.32	
**Metronidazole**											
500mg tds 7 days	Xajigeorgiou et al. (2006)	6 weeks	12/12	4.71 ± 0.57	3.47 ± 0.51	1.24 ± 0.22	12/11	4.21 ± 0.74	3.19 ± 0.59	1.02 ± 0.29	MD ± SD not reported, PP
		6 mths.	12/12	4.71 ± 0.57	2.86 ± 0.65	1.85 ± 0.25	12/11	4.21 ± 0.74	3.52 ± 0.76	0.69 ± 0.32	

Dosage	Author (Year)	Follow-up	Intervention				Control				Notes
			Sample size (BL/End)	Baseline (Mean ± SD)	Follow-up (Mean ± SD)	PPD reduction (Mean ± SD)	Sample size (BL/End)	Baseline (Mean ± SD)	Follow-up (Mean ± SD)	PPD reduction (Mean ± SD)	
500mg bd 8 days	Sigusch et al. (2001)	6 mths.	15/15	5.80 ± 0.73	3.60 ± 0.78	2.20 ± 0.28	10/10	5.90 ± 0.70	4.60 ± 1.00	1.30 ± 0.39	MD ± SD not reported, ITT
		24 mths.	15/15	5.80 ± 0.73	3.20 ± 0.65	2.60 ± 0.25	10/10	5.90 ± 0.70	5.20 ± 0.77	0.70 ± 0.33	
400mg tds 14 days	Silva et al. (2011)	3 mths.	17/17	3.60 ± 0.60	2.90 ± 0.50	0.76 ± 0.48	17/17	3.60 ± 0.50	3.00 ± 0.60	0.63 ± 0.26	ITT
**Moxifloxacin**											
400mg od 7 days	Ardila et al. (2015)	3 mths.	20/20	4.27 ± 0.40	3.12 ± 0.60	1.15 ± 0.16	20/20	4.34 ± 0.50	3.67 ± 0.40	0.67 ± 0.14	MD ± SD not reported, ITT
		6 mths.	20/20	4.76 ± 0.70	3.08 ± 0.10	1.68 ± 0.20	20/20	4.80 ± 0.70	4.06 ± 0.20	0.74 ± 0.21	
400mg qid 7 days	Ardila et al. (2020)	3 mths.	12/12	4.27 ± 0.40	3.08 ± 0.60	1.19 ± 0.16	12/11	4.34 ± 0.50	3.50 ± 0.40	0.84 ± 0.14	
		6 mths.	12/12	4.76 ± 0.70	3.02 ± 0.20	1.74 ± 0.21	12/11	4.80 ± 0.70	4.02 ± 0.30	0.78 ± 0.22	
**Satranidazole**											
300mg bd 10 days	Pradeep et al. (2013)	1 mth.	33/30	7.91 ± 0.92	5.52 ± 1.07	2.41 ± 1.09	33/30	7.71 ± 0.98	6.75 ± 1.08	0.96 ± 1.19	PP
		3 mths.	33/30	7.91 ± 0.92	4.51 ± 1.09	3.55 ± 1.54	33/30	7.71 ± 0.98	6.13 ± 1.06	1.63 ± 1.55	PP
		6 mths.	33/30	7.91 ± 0.92	4.17 ± 1.12	3.84 ± 1.31	33/30	7.71 ± 0.98	6.32 ± 0.98	1.42 ± 1.01	PP
**Secnidazole**											
1g od 30 days	Li et al. (2017)	3 mths.	25/25	6.40 ± 1.60	5.40 ± 1.20	1.00 ± 0.40	25/25	6.10 ± 1.20	5.30 ± 0.80	0.80 ± 0.29	MD ± SD not reported, ITT
**Minocycline HCl**											
200mg then 100mg od 14 days	Basegmez et al. (2011)	1 mth.	20/20	4.84 ± 0.52	2.97 ± 2.12	1.87 ± 0.49	20/20	4.67 ± 0.49	2.97 ± 2.56	1.70 ± 0.58	Follow-up not reported, RAL, Median (Min – Max) converted to Mean (SD), ITT
		3 mths.	20/20	4.84 ± 0.52	2.96 ± 1.99	1.88 ± 0.46	20/20	4.67 ± 0.49	2.99 ± 2.82	1.68 ± 0.64	
		6 mths.	20/20	4.84 ± 0.52	2.93 ± 2.40	1.91 ± 0.55	20/20	4.67 ± 0.49	2.96 ± 3.16	1.71 ± 0.72	

Table S8. Summary of BOP outcome reported in included studies

Dosage	Author (Year)	Follow-up	Intervention				Control				Notes
			Sample size (BL/End)	Baseline (Mean ± SD)	Follow-up (Mean ± SD)	BOP reduction (Mean ± SD)	Sample size (BL/End)	Baseline (Mean ± SD)	Follow-up (Mean ± SD)	BOP reduction (Mean ± SD)	
**Amoxycillin + Metronidazole**											
500mg + 500mg tds 7 days	Ardila et al. (2020)	3 mths.	12/11	46.10 ± 3.00	8.10 ± 1.50	38.00 ± 1.01	12/11	45.50 ± 4.00	9.10 ± 0.80	36.40 ± 1.18	MD ± SD not reported, ITT
	Ardila et al. (2020)	6 mths.	12/11	46.10 ± 3.00	8.00 ± 1.20	38.10 ± 0.97	12/11	45.50 ± 4.00	8.90 ± 0.50	36.60 ± 1.16	
500mg + 400mg tds 7 days	Borges et al. (2017)	3 mths.	22/20	68.00 ± 21.20	31.90 ± 15.10	36.10 ± 5.80	22/19	67.90 ± 14.60	42.90 ± 17.30	25.00 ± 4.83	
		6 mths.	22/20	68.00 ± 21.20	30.70 ± 14.30	37.30 ± 5.70	22/19	64.90 ± 18.50	39.00 ± 17.40	28.90 ± 4.84	
		12 mths.	22/20	68.00 ± 21.20	28.60 ± 15.30	39.40 ± 5.83	22/19	64.90 ± 18.50	40.00 ± 17.40	27.90 ± 4.84	
500mg + 400mg tds 14 days	Liaw et al. (2019)	2 mths.	13/12	38.86 ± 17.36	12.67 ± 9.14	26.19 ± 5.44	13/11	49.67 ± 13.23	25.35 ± 23.24	24.32 ± 8.06	Median (IQR) converted to mean (SD), PP
	Borges et al. (2017)	3 mths.	22/20	69.10 ± 20.50	28.70 ± 13.10	40.40 ± 5.44	22/19	67.90 ± 14.60	42.90 ± 17.30	25.00 ± 4.83	MD ± SD not reported, ITT
	Silva et al. (2011)		17/17	72.90 ± 17.20	31.50 ± 25.40	41.40 ± 7.44	17/17	70.90 ± 22.40	32.80 ± 25.10	38.10 ± 8.16	
	Borges et al. (2017)	6 mths.	22/20	69.10 ± 20.50	26.80 ± 9.30	42.30 ± 5.03	22/19	64.90 ± 18.50	39.00 ± 17.40	28.90 ± 4.84	
		12 mths.	22/20	69.10 ± 20.50	25.10 ± 11.60	44.00 ± 5.27	22/19	64.90 ± 18.50	40.00 ± 17.40	27.90 ± 4.84	
500mg + 250mg tds 7 days	Moeintaghavi et al. (2007)	2 mths.	28/28	96.00 ± 10.98	32.23 ± 17.82	63.80 ± 19.50	22/22	93.10 ± 12.48	38.50 ± 13.07	50.60 ± 22.30	
	Borges et al. (2017)	3 mths.	22/21	64.90 ± 18.50	32.50 ± 12.90	32.40 ± 4.92	22/19	67.90 ± 14.60	42.90 ± 17.30	25.00 ± 4.83	
		6 mths.	22/21	64.90 ± 18.50	27.10 ± 10.30	37.80 ± 4.62	22/19	64.90 ± 18.50	39.00 ± 17.40	28.90 ± 4.84	
		12 mths.	22/21	64.90 ± 18.50	24.00 ± 12.90	40.90 ± 4.92	22/19	64.90 ± 18.50	40.00 ± 17.40	27.90 ± 4.84	
500mg + 250mg tds 14 days	Borges et al. (2017)	3 mths.	22/20	62.80 ± 18.90	24.30 ± 14.10	38.50 ± 5.27	22/19	67.90 ± 14.60	42.90 ± 17.30	25.00 ± 4.83	
		6 mths.	22/20	62.80 ± 18.90	23.10 ± 13.00	39.70 ± 5.13	22/19	64.90 ± 18.50	39.00 ± 17.40	28.90 ± 4.84	

Dosage	Author (Year)	Follow-up	Intervention				Control				Notes
			Sample size (BL/End)	Baseline (Mean ± SD)	Follow-up (Mean ± SD)	BOP reduction (Mean ± SD)	Sample size (BL/End)	Baseline (Mean ± SD)	Follow-up (Mean ± SD)	BOP reduction (Mean ± SD)	
		12 mths.	22/20	62.80 ± 18.90	18.50 ± 12.70	44.30 ± 5.09	22/19	64.90 ± 18.50	40.00 ± 17.40	27.90 ± 4.84	
500mg + 200mg tds 7 days	Saleh et al. (2016)	3 mths.	13/11	55.50 ± 19.80	16.20 ± 10.30	39.30 ± 20.10	12/10	52.20 ± 12.70	19.50 ± 10.10	32.60 ± 12.50	PP
**Azithromycin**											
500mg od 3 days	Han et al. (2012)		18/14	70.77 ± 25.70	20.86 ± 31.41	51.36 ± 20.70	18/14	65.23 ± 18.50	18.16 ± 60.08	47.07 ± 16.80	Follow-up not reported, PP
	Liaw et al. (2019)	2 mths.	12/11	37.53 ± 21.29	14.13 ± 5.85	23.40 ± 6.66	13/11	49.67 ± 13.23	25.35 ± 23.24	24.32 ± 7.42	Median (IQR) converted to mean (SD), PP
	Emingil et al. (2012)	3 mths.	18/16	74.75 ± 16.48	18.22 ± 7.08	56.53 ± 4.48	18/16	72.47 ± 13.45	21.19 ± 8.66	51.28 ± 3.77	95% CI converted to mean (SD), PP
	Gomi et al. (2007)		17/17	31.42 ± 17.15	4.46 ± 3.27	26.96 ± 4.23	17/17	31.43 ± 18.46	12.08 ± 7.15	19.35 ± 4.80	MD ± SD not reported, ITT
	Han et al. (2012)		18/14	70.77 ± 25.70	19.41 ± 73.06	53.43 ± 20.30	18/14	65.23 ± 18.50	17.23 ± 66.70	48.00 ± 18.50	Follow-up not reported, PP
	Saleh et al. (2016)		12/11	42.30 ± 19.90	13.90 ± 6.00	28.40 ± 10.60	12/10	52.20 ± 12.70	19.50 ± 10.10	32.60 ± 12.50	PP
	Čuk et al. (2020)	6 mths.	20/19	70.00 ± 32.04	16.40 ± 8.01	53.60 ± 7.58	20/19	76.40 ± 24.03	23.60 ± 24.03	52.80 ± 7.58	Median (IQR) converted to mean (SD), PP
	Emingil et al. (2012)		18/16	74.75 ± 16.48	17.89 ± 6.98	56.86 ± 4.47	18/16	72.47 ± 13.45	18.97 ± 6.31	53.50 ± 3.50	95% CI converted to mean (SD), PP
	Gomi et al. (2007)		17/17	31.42 ± 17.15	5.35 ± 2.91	26.07 ± 4.22	17/17	31.43 ± 18.46	12.91 ± 7.62	18.52 ± 4.84	MD ± SD not reported, ITT
	Han et al. (2012)		18/14	70.77 ± 25.70	17.34 ± 71.48	54.08 ± 19.30	18/14	65.23 ± 18.50	14.94 ± 61.64	50.29 ± 17.20	Follow-up not reported, PP
	Haas et al. (2008)	12 mths.	12/12	65.90 ± 24.50	20.86 ± 31.41	45.04 ± 11.50	13/12	76.40 ± 21.80	31.94 ± 41.30	44.46 ± 13.48	MD (SE) converted to MD (SD), PP
	Povšič et al. (2021)		20/19	70.00 ± 32.04	13.66 ± 10.41	51.29 ± 24.11	20/19	76.40 ± 24.03	14.25 ± 14.42	56.58 ± 15.06	PP
500mg od 5 days	Morales et al. (2021)	3 mths.	16/16	57.40 ± 10.20	41.90 ± 10.90	14.70 ± 11.10	15/15	52.50 ± 12.60	40.80 ± 13.30	0.20 ± 0.20	ITT
	Morales et al. (2021)	6 mths.	16/16	57.40 ± 10.20	44.10 ± 13.50	13.20 ± 14.90	15/15	52.50 ± 12.60	42.30 ± 15.30	0.20 ± 0.30	ITT
	Sampaio et al. (2011)		20/20	75.78 ± 24.51	9.91 ± 7.24	65.87 ± 5.71	20/20	81.93 ± 12.86	12.31 ± 8.83	69.62 ± 3.49	MD ± SD not reported, ITT

Dosage	Author (Year)	Follow-up	Intervention				Control				Notes
			Sample size (BL/End)	Baseline (Mean ± SD)	Follow-up (Mean ± SD)	BOP reduction (Mean ± SD)	Sample size (BL/End)	Baseline (Mean ± SD)	Follow-up (Mean ± SD)	BOP reduction (Mean ± SD)	
	Morales et al. (2021)	9 mths.	16/16	57.40 ± 10.20	48.10 ± 14.10	9.20 ± 15.00	15/15	52.50 ± 12.60	45.20 ± 13.10	0.10 ± 0.30	ITT
	Morales et al. (2021)	12 mths.	16/16	57.40 ± 10.20	42.30 ± 13.30	15.00 ± 12.60	15/15	52.50 ± 12.60	40.80 ± 11.50	0.20 ± 0.20	ITT
	Sampaio et al. (2011)		20/19	75.78 ± 24.51	10.01 ± 5.52	65.77 ± 5.76	20/19	81.93 ± 12.86	9.47 ± 7.71	72.46 ± 3.35	MD ± SD not reported, ITT
**Clarithromycin**											
500mg bd 3 days	Andere et al. (2017)	3 mths	20/20	53.00 ± 19.00	15.00 ± 6.00	38.00 ± 4.46	20/20	54.00 ± 30.00	18.00 ± 5.90	36.00 ± 6.84	MD ± SD not reported, ITT
	Andere et al. (2017)	6 mths.	20/20	53.00 ± 19.00	13.30 ± 5.00	39.70 ± 4.39	20/20	54.00 ± 30.00	18.00 ± 6.85	36.00 ± 6.88	
	Bechara Andere et al. (2018)		18/18	52.00 ± 19.00	13.00 ± 5.00	39.00 ± 4.63	18/18	59.00 ± 32.00	20.00 ± 8.00	39.00 ± 7.77	
**Metronidazole**											
400mg tds 14 days	Silva et al. (2011)	3 mths.	17/17	73.30 ± 22.40	15.70 ± 19.30	57.60 ± 7.17	17/17	70.90 ± 22.40	32.80 ± 25.10	38.10 ± 8.16	MD ± SD not reported, ITT
**Moxifloxacin**											
400mg od 7 days	Ardila et al. (2015)	3 mths.	20/20	44.00 ± 10.00	9.30 ± 0.60	34.70 ± 1.76	20/20	47.00 ± 16.00	12.70 ± 0.40	34.30 ± 3.58	MD ± SD not reported, ITT
		6 mths.	20/20	44.00 ± 10.00	9.40 ± 0.60	34.60 ± 2.24	20/20	47.00 ± 16.00	10.20 ± 0.40	36.80 ± 3.58	
400mg qid 7 days	Ardila et al. (2020)	3 mths.	12/12	46.20 ± 6.00	8.80 ± 1.10	37.40 ± 1.74	12/11	45.50 ± 4.00	9.10 ± 0.80	36.40 ± 1.18	
		6 mths.	12/12	46.20 ± 6.00	8.50 ± 0.70	37.70 ± 1.74	12/11	45.50 ± 4.00	8.90 ± 0.50	36.60 ± 1.16	

Table S9. Summary of adverse events reported in included studies

Dosages	Authors	Test		Control	
		Adverse events	n	Adverse events	n
**Amoxicillin + Metronidazole**					
500mg + 500mg tds 7 days	Ardila et al. (2020)	Diarrhoea	3	None	
		Nausea	3		
		Vomiting	3		
	Xajigeorgiou et al. (2006)	Mild gastrointestinal discomfort	2	None	
500mg + 400mg tds 7 days	Borges et al. (2017)	Nausea	3	Nausea	1
		Diarrhoea	1	Diarrhoea	1
		Metallic taste	3	Metallic taste	2
		Headache or dizziness	4	Headache or dizziness	1
		Irritability or bad mood	2	Excessive sleep	2
		Weakness	2		
		Excessive sleep	3		
	Dukić et al. (2016)	Nausea, dizziness and/or anorexia	NR	None	
	Harks et al. (2015)	Serious adverse events (e.g., allergic reaction, anaphylactic reaction)	3	Serious adverse events (e.g., allergic reaction)	4
500mg + 400mg tds 14 days	Borges et al. (2017)	Nausea	3	Nausea	1
		Diarrhoea	3	Diarrhoea	1
		Metallic taste	6	Metallic taste	2
		Headache or dizziness	6	Headache or dizziness	1
		Irritability or bad mood	1	Excessive sleep	2
		Weakness	3		
		Excessive sleep	2		
	Liaw et al. (2019)	Mild gastrointestinal discomfort	3	None	
	Silva et al. (2011)	Headache	2	Metallic taste	1
		Metallic taste	3	Weakness	2
		Vomiting	3		
		Irritability	1		
500mg + 250mg tds 7 days	Borges et al. (2017)	Nausea	2	Nausea	1
		Diarrhoea	5	Diarrhoea	1
		Metallic taste	6	Metallic taste	2
		Headache or dizziness	5	Headache or dizziness	1
		Irritability or bad mood	1	Excessive sleep	2
		Weakness	2		
		Excessive sleep	5		
	Moeintaghavi et al. (2007)	Gastrointestinal disturbance	1	None	
500mg + 250mg tds 14 days	Borges et al. (2017)	Nausea	2	Nausea	1

Dosages	Authors	Test		Control	
		Adverse events	n	Adverse events	n
		Diarrhoea	2	Diarrhoea	1
		Metallic taste	7	Metallic taste	2
		Headache or dizziness	2	Headache or dizziness	1
		Irritability or bad mood	1	Excessive sleep	2
		Weakness	1		
		Excessive sleep	4		
500mg + 200mg tds 7 days	Saleh et al. (2016)	Stomach upset and nausea	2	None	
		Diarrhoea	1		
		Stomach upset and diarrhoea	2		
375mg + 250mg tds 7 days	Ribeiro et al. (2009)	Nausea and diarrhoea	4	None	
	Taiete et al. (2016)	Gastrointestinal discomfort	4	Gastrointestinal discomfort	1
	Winkel et al. (2001)	Gastrointestinal intolerance	9	Rash	1
		Rash	2		
		Nausea	1		
**Azithromycin**					
500mg od 3 days	Čuk et al. (2020)	Headaches	2	Nausea	1
				Headaches	1
	Gomi et al. (2007)	Diarrhoea	1	None	
	Haas et al. (2008)	None		None	
	Han et al. (2012)	None		None	
	Emingil et al. (2012)	None		None	
	Liaw et al. (2019)	Mild gastrointestinal discomfort	1	None	
	Saleh et al. (2016)	Stomach upset	2	None	
		Diarrhoea	1		
		Increased frequency of urination	1		
500mg od 5 days	Sampaio et al. (2011)	Diarrhoea	2	Headache and dizziness	2
		Headache and dizziness	1	Excessive sleepiness	3
		Excessive sleepiness	3	Metallic taste	3
		Metallic taste	2	General unwellness	1
		General unwellness	1		
500mg qid 5 days	Morales et al. (2021)	Nausea	1	None	
**Cefixime**					
400mg od 7 days	Dukić et al. (2016)	Nausea, dizziness and/or anorexia	NR	None	
**Clarithromycin**					
500mg bd 3 days	Andere et al. (2017)	Gastrointestinal discomfort	2	None	
	Bechara Andere et al. (2018)	Gastrointestinal discomfort		None	
500mg tds 7 days	Suryaprasanna et al. (2018)	None		None	

Dosages	Authors	Test		Control	
		Adverse events	n	Adverse events	n
**Doxycycline**					
200mg then 100mg od 14 days	Xajigeorgiou et al. (2006)	None		None	
**Metronidazole**					
500mg tds 7 days	Xajigeorgiou et al. (2006)	Metallic taste	1	None	
400mg tds 14 days	Silva et al. (2011)	Diarrhoea	1	Metallic taste	1
		Headache	1	Weakness	2
		Metallic taste	3	Irritability	1
		Vomiting	1		
		Irritability	1		
**Moxifloxacin**					
400mg od 7 days	Ardila et al. (2015)	None		None	
400mg qid 7 days	Ardila et al. (2020)	None		None	
**Satranidazole**					
300mg bd 10 days	Pradeep et al. (2013)	Taste alteration	3	None	
		Nausea	1		

Table S10. The authors' judgement and support for judgements for each risk of bias of all included studies

Domain	Randomisation process		Deviations from intended interventions		Missing outcome data		Measurement of outcome		Selection of the reported result		Overall bias
Study	Authors' judgement	Support for judgement	Authors' judgement	Support for judgement	Authors' judgement	Support for judgement	Authors' judgement	Support for judgement	Authors' judgement	Support for judgement	
Andere et al., 2017	Low	Computer-generated; independent personnel; concealed in sealed envelopes; no baseline characteristics imbalances.	Low	Identical placebos; blinded clinician; ITT.	Low	No dropouts; ITT.	Low	Appropriate method; calibrated and blinded examiner.	Low	Pre-specified analysis conducted and reported.	Low

Domain	Randomisation process		Deviations from intended interventions		Missing outcome data		Measurement of outcome		Selection of the reported result		Overall bias
Study	Authors' judgement	Support for judgement	Authors' judgement	Support for judgement	Authors' judgement	Support for judgement	Authors' judgement	Support for judgement	Authors' judgement	Support for judgement	
Ardila et al. (2015)	Low	Computer-generated permuted block; independent personnel; opaque envelopes; no baseline characteristics imbalances.	Low	Identical placebo; blinded clinician; ITT.	Low	4/40 dropouts; ITT.	Low	Appropriate method; blinded and calibrated examiner.	Low	Pre-specified analysis conducted and reported.	Low
Ardila et al. (2020)	Low	Computer-generated permuted block; independent personnel; opaque envelopes; no baseline characteristics imbalances.	Low	Identical placebo; triple blind; ITT.	Low	2/36 dropouts; ITT.	Low	Appropriate method; blinded and calibrated examiner.	Low	Pre-specified analysis conducted and reported.	Low
Basegmez et al. (2011)	Some concerns	Computer-generated; No information on allocation concealment; no baseline characteristics imbalances.	Low	Identical placebo; blinded clinician; ITT.	Low	No dropouts; ITT.	Low	Appropriate method; calibrated and blinded examiner.	Low	Pre-specified analysis conducted and reported.	Some concerns
Bechara Andere et al. (2018)	Low	Computer-generated; independent personnel; no imbalances.	Low	Identical placebo; blinded clinician; ITT.	Low	No dropouts; ITT.	Low	Appropriate method; calibrated and blinded examiner.	Low	Pre-specified analysis conducted and reported.	Low

Domain	Randomisation process		Deviations from intended interventions		Missing outcome data		Measurement of outcome		Selection of the reported result		Overall bias
Study	Authors' judgement	Support for judgement	Authors' judgement	Support for judgement	Authors' judgement	Support for judgement	Authors' judgement	Support for judgement	Authors' judgement	Support for judgement	
Borges et al. (2017)	Low	Computer-generated; independent personnel; no imbalances.	Low	Identical placebos; blinded clinician; ITT.	Low	10/110 dropouts; ITT.	Low	Appropriate method; calibrated and blinded examiner.	Low	Pre-specified analysis conducted and reported.	Low
Buchmann et al. (2010)	Low	Randomisation (unclear); independent personnel; no information on baseline characteristics imbalances.	Low	Identical placebos; blinded clinician; ITT	Low	No dropouts; ITT	Low	Appropriate method; calibrated and blinded examiner.	Low	Pre-specified analysis conducted and reported.	Low
Čuk et al, (2020)	Low	Computer-generated; independent personnel; baseline characteristics included only those analysed.	High	Identical placebo; blinded clinician; PP analysis; characteristics of dropouts not described.	Low	2/40 dropouts; PP; dropouts due to personal reasons.	Low	Appropriate method; blinded and calibrated examiner	Low	Pre-specified analysis conducted and reported.	High
Dukić et al. (2016)	Some concerns	Randomisation done; No information on allocation concealment and baseline characteristics imbalances	High	No placebo; no information on clinician; number of subjects included in the analysis not reported.	High	Number of subjects completed and included in the analysis not reported.	Some concerns	Appropriate method; No information on examiner; clinical assessment does not require judgement.	High	Number of included participants not reported.	High

Domain	Randomisation process		Deviations from intended interventions		Missing outcome data		Measurement of outcome		Selection of the reported result		Overall bias
**Study**	**Authors' judgement**	**Support for judgement**	**Authors' judgement**	**Support for judgement**	**Authors' judgement**	**Support for judgement**	**Authors' judgement**	**Support for judgement**	**Authors' judgement**	**Support for judgement**	
Emingil et al. (2012)	Low	Computer-generated; independent personnel; baseline characteristics included only those analysed.	High	Identical placebo; blinded clinician; PP analysis; characteristics of dropouts not described.	Low	4/36 dropouts; PP; dropouts due to personal reasons.	Low	Appropriate methods; calibrated and blinded examiner.	Low	Pre-specified analysis conducted and reported.	High
Fonseca et al. (2015)	Low	Simple randomisation (unclear); sealed and opaque envelopes; baseline characteristics included only those analysed.	High	No placebo; blinded clinician; PP analysis; characteristics of dropouts not described.	Low	3/60 dropouts; PP; >95% available data.	Low	Appropriate method; calibrated and blinded examiner.	Low	Pre-specified analysis conducted and reported.	High
Gomi et al. (2007)	Some concerns	Randomisation done (unclear); No information on allocation concealment; no baseline characteristics imbalances	Low	No placebo; No information on clinician; according to study protocol; ITT.	Low	No dropouts; ITT.	Some concerns	No information on method and examiner; clinical assessment does not require judgement.	Low	Pre-specified analysis conducted and reported.	High
Haas et al. (2008)	Low	Means of draw; independent personnel; no baseline characteristics imbalances	High	Identical placebos; blinded clinician; PP analysis; characteristics of dropouts not described.	Low	1/25 dropout; PP; >95% available data	Low	Appropriate method; calibrated and blinded examiner.	Low	Pre-specified analysis conducted and reported.	High

Domain	Randomisation process		Deviations from intended interventions		Missing outcome data		Measurement of outcome		Selection of the reported result		Overall bias
Study	Authors' judgement	Support for judgement	Authors' judgement	Support for judgement	Authors' judgement	Support for judgement	Authors' judgement	Support for judgement	Authors' judgement	Support for judgement	
Han et al. (2012)	Low	Computer-generated; independent personnel; baseline characteristics included only those analysed.	High	Identical placebos; blinded clinician; PP analysis; characteristics of dropouts not described.	Low	8/36 dropouts; PP; dropouts due to personal reasons.	Low	Appropriate method; calibrated and blinded examiner.	Low	Pre-specified analysis conducted and reported.	High
Harks et al. (2015)	Low	Quad-block randomisation; independent personnel; no baseline characteristics imbalances.	High	Identical placebo; blinded clinician; PP analysis; characteristics of dropouts not described.	High	161/506 dropouts; PP; dropouts due to SAE.	Low	Appropriate method; calibrated and blinded examiner.	Low	Pre-specified analysis conducted and reported.	High
Li et al. (2017)	Some concerns	Randomisation done (unclear); no information on allocation concealment and baseline characteristics imbalances.	Low	Placebo; no information on clinician; ITT	Low	No dropouts; ITT	Some concerns	No information on method and calibration; blinded examiner.	Low	Pre-specified analysis conducted and reported	High
Liaw et al. (2019)	Low	Block randomisation; sealed and opaque envelope; no baseline characteristics imbalances	High	No placebo; blinded clinician; consistent to study protocol; PP analysis; characteristics of dropouts not described.	Low	4/38 dropouts; PP; proportions of missing outcome between groups were comparable.	Low	Appropriate method; calibrated and blinded examiner.	Low	Pre-specified analysis conducted and reported	High

Domain	Randomisation process		Deviations from intended interventions		Missing outcome data		Measurement of outcome		Selection of the reported result		Overall bias
Study	Authors' judgement	Support for judgement	Authors' judgement	Support for judgement	Authors' judgement	Support for judgement	Authors' judgement	Support for judgement	Authors' judgement	Support for judgement	
Moeintaghavi et al. (2007)	Low	Coded study medication (unclear); sealed master code; no baseline characteristics imbalances.	Low	Identical placebo; blinded clinician; ITT.	Low	No dropouts; ITT.	Low	Appropriate method; calibrated and blinded examiner.	Low	Pre-specified analysis conducted and reported	Low
Morales et al. (2018)	Low	Computer-generated; independent personnel; no baseline characteristics imbalances.	Low	Identical placebo; blinded clinician; ITT.	Low	No dropouts; ITT.	Low	Appropriate method; calibrated and blinded examiner.	Low	Pre-specified analysis conducted and reported	Low
Povšič et al. (2021)	Low	Computer-generated; independent personnel; no baseline characteristics imbalances	High	Identical placebo; blinded clinician; PP analysis; characteristics of dropouts not described	Low	2/40 dropouts; PP; >95% available data	Low	Appropriate method; calibrated and blinded examiner.	Low	Pre-specified analysis conducted and reported	High
Pradeep et al. (2013)	Low	Computer-generated; independent personnel; baseline characteristics included only those analysed.	High	Identical placebo; blinded clinician; PP analysis; characteristics of dropouts not described.	High	6/66 dropouts; PP; no description on reason of missingness	Low	Appropriate method; calibrated and blinded examiner.	Low	Pre-specified analysis conducted and reported	High
Ribeiro et al. (2009)	Low	Computer-generated; independent personnel; baseline characteristics included only those analysed.	High	Placebo; blinded clinician; PP analysis; characteristics of dropouts not described.	Low	3/25 dropouts; PP; dropouts due to personal reasons.	Low	Appropriate method; calibrated and blinded examiner.	Low	Pre-specified analysis conducted and reported	High

Domain	Randomisation process		Deviations from intended interventions		Missing outcome data		Measurement of outcome		Selection of the reported result		Overall bias
Study	Authors' judgement	Support for judgement	Authors' judgement	Support for judgement	Authors' judgement	Support for judgement	Authors' judgement	Support for judgement	Authors' judgement	Support for judgement	
Saleh et al. (2016)	Low	Computer-generated; independent personnel; no baseline characteristics imbalances	High	Identical placebo; blinded clinician; PP analysis; characteristics of dropouts not described.	Low	5/37 dropouts; PP; no difference in proportions of missing data between both groups.	Low	Appropriate method; calibrated and blinded examiner.	Low	Pre-specified analysis conducted and reported	High
Sampaio et al. (2011)	Low	Computer-generated; independent personnel; no baseline characteristics imbalances	Low	Identical placebo; blinded clinician; ITT.	Low	2/40 dropouts; ITT.	Low	Appropriate method; calibrated and blinded examiner.	Low	Pre-specified analysis conducted and reported	Low
Sigusch et al. (2001)	Some concerns	Randomisation done (unclear); no information on the allocation concealment; no baseline characteristics imbalances.	Low	No placebo; no information on clinician; no deviations from study protocol; ITT.	Low	No dropouts; ITT.	Low	Appropriate method; calibrated and blinded examiner.	Low	Pre-specified analysis conducted and reported	Some concerns
Silva et al. (2011)	Low	Computer-generated; independent personnel; no baseline characteristics imbalances	Low	Placebo in identical opaque plastic bottle; blinded clinician; ITT.	Low	No dropouts; ITT.	Low	Appropriate method; calibrated and blinded examiner.	Low	Pre-specified analysis conducted and reported	Low
Suryaprasanna et al. (2018)	Some concerns	Randomisation done (unclear); no information on allocation concealment; no apparent baseline characteristics imbalances.	Low	No placebo; blinded clinician; no deviations from study protocol; ITT.	Low	No dropouts; ITT.	Low	Appropriate method; by one examiner; no information on calibration; blinded examiner.	Low	Pre-specified analysis conducted and reported	Some concerns

Domain	Randomisation process		Deviations from intended interventions		Missing outcome data		Measurement of outcome		Selection of the reported result		Overall bias
**Study**	**Authors' judgement**	**Support for judgement**	**Authors' judgement**	**Support for judgement**	**Authors' judgement**	**Support for judgement**	**Authors' judgement**	**Support for judgement**	**Authors' judgement**	**Support for judgement**	
Taiete et al. (2016)	Low	Computer-generated; independent personnel; baseline characteristics included only those analysed.	High	No placebo; blinded clinician; PP analysis; characteristics of dropouts not described	Low	9/48 dropouts; PP analysis; dropouts due to personal reasons	Low	Appropriate method; calibrated and blinded examiner.	Low	Pre-specified analysis conducted and reported	High
Winkel et al. (2001)	Some concerns	Randomisation done (unclear); no information on allocation concealment and baseline characteristics imbalances.	High	Identical placebo; no information on clinician; PP analysis; characteristics of dropouts not described.	High	4/54 dropouts; PP analysis; 2 dropouts due to AE.	Some concerns	Appropriate method; no information on calibration; blinded examiner.	Low	Pre-specified analysis conducted and reported	High
Xajigeorgiou et al. (2006)	Low	Random tables; independent personnel; baseline characteristics included only those analysed.	High	No placebo; blinded clinician; PP analysis; characteristics of dropouts not described	Low	5/48 dropouts; PP analysis; dropouts due to personal reasons.	Low	Appropriate method; calibrated and blinded examiner.	Low	Pre-specified analysis conducted and reported	High

Table S11. Pairwise comparisons of interventions with direct comparisons and source of heterogeneity in the substantially heterogenous comparison

No.	Comparisons		m	o	MD [95% CI] (IV, Random)	I²	Outliers	Influential cases	Large overall heterogeneity contribution
	Treatment 1	Treatment 2							
**Systemic antibiotics: Short-term CAL gain**									
1.	SRP + CLM	SRP + Placebo/alone	2	70	-0.22 [-0.30, -0.14]	0%			
2.	SRP + MOX	SRP + Placebo/alone	2	64	-0.74 [-0.92, -0.55]	67.7%	None	None	Ardila et al. (2020)
3.	SRP + AMOX + MET	SRP +MOX	1	24	0.09 [-0.11, 0.29]				
4.	SRP + AMOX + MET	SRP + Placebo/alone	12	466	-0.29 [-0.46, -0.12]	83.7%	Ardila et al. (2020)<br>Silva et al. (2011)<br>Buchmann et al. (2010)	Ardila et al. (2020)<br>Silva et al. (2011)	Ardila et al. (2020)<br>Silva et al. (2011)
5.	SRP + MINO HCl	SRP + Placebo/alone	2	80	-0.09 [-0.30, 0.11]	0%	-	-	
6.	SRP + AZ	SRP + Placebo/alone	10	285	-0.10 [-0.27, 0.07]	78.1%	Gomi et al., (2007)	Gomi et al., (2007)	Gomi et al., (2007)
7.	SRP + SEC	SRP + Placebo/alone	1	50	-0.20 [-0.42, 0.02]				
8.	SRP + SZ	SRP + Placebo/alone	2	120	-1.86 [-2.34, -1.38]	0%			
9.	SRP + AMOX + MET	SRP + AZ	2	45	-0.07 [-0.69, 0.54]	81.9%	None	None	Saleh et al. (2016)
10.	SRP + AMOX + MET	SRP + MET	2	56	-0.08 [-0.33, 0.17]	9.8%			
11.	SRP + MET	SRP + Placebo/alone	2	57	-0.19 [-0.13, 0.51]	58.2%	None	None	Xajigeorgiou et al. (2006)
12.	SRP + AMOX + MET	SRP + DOX	1	20	-0.06 [-0.57, 0.45]				
13.	SRP + DOX	SRP + MET	1	22	0.14 [-0.38, 0.66]				
14.	SRP + DOX	SRP + Placebo/alone	1	21	0.15 [-0.32, 0.62]				
15.	SRP + AMOX + MET	SRP + CEF	1	60	-0.04 [-0.18, 0.10]				
16.	SRP + CEF	SRP + Placebo/alone	1	60	-0.12 [-0.26, 0.02]				
**Systemic antibiotics intermediate-term CAL gain**									
1.	SRP + CLM	SRP + Placebo/alone	3	106	-0.11 [-0.44, 0.22]	91.2%	None	Bechara Andere et al. (2018)	Bechara Andere et al. (2018)
2.	SRP + MOX	SRP + Placebo/alone	2	64	-0.81 [-1.20, -0.42]	92.8%	None	None	Ardila et al. (2020)
3.	SRP + AMOX + MET	SRP + Placebo/alone	9	334	-0.46 [-0.61, -0.31]	69.8%	None	Ardila et al. (2020)	Ardila et al. (2020)
4.	SRP + MOX	SRP + AMOX + MET	1	24	-0.11 [-0.31, 0.09]				
5.	SRP + MINO HCl	SRP + Placebo/alone	1	40	-0.07 [-0.33, 0.19]				
6.	SRP + AZ	SRP + Placebo/alone	9	294	-0.15 [-0.40, 0.10]	84.1%	Gomi et al., (2007)	Gomi et al., (2007)	Gomi et al., (2007)

No.	Comparisons		m	o	MD [95% CI] (IV, Random)	I²	Outliers	Influential cases	Large overall heterogeneity contribution
	Treatment 1	Treatment 2							
7.	SRP + SZ	SRP + Placebo/alone	1	60	-2.07 [-2.71, -1.43]				
8.	SRP + Clindamycin	SRP + Placebo/alone	1	21	-1.10 [-1.45, -0.75]				
9.	SRP + DOX	SRP + Placebo/alone	2	43	-0.52 [0.79, -0.25]	0%			
10.	SRP + MET	SRP + Placebo/alone	2	48	-0.98 [-1.62, -0.34]	87.6%	None	None	Xajigeorgiou et al. (2006)
11.	SRP + Clindamycin	SRP + DOX	1	23	-0.50 [-0.83, -0.17]				
12.	SRP + Clindamycin	SRP + MET	1	26	0.20 [-0.10, 0.50]				
13.	SRP + DOX	SRP + MET	2	49	0.59 [0.25, 0.93]	30.3%			
14.	SRP + DOX	SRP + AMOX + MET	1	20	0.11 [-0.46, 0.69]				
15.	SRP + MET	SRP + AMOX + MET	1	22	-0.21 [-0.65, 0.23]				
**Systemic antibiotics: Long-term CAL gain**									
1.	SRP + AZ	SRP + Placebo/alone	4	136	-0.03 [-0.40, 0.35]	60.2%	None	None	Haas et al. (2008)
2.	SRP + Clindamycin	SRP + Placebo/alone	1	21	-1.50 [-1.81, -1.19]				
3.	SRP + DOX	SRP + Placebo/alone	1	22	-0.50 [-0.81, -0.19]				
4.	SRP + MET	SRP + Placebo/alone	1	25	-1.80 [-2.09, -1.51]				
5.	SRP + Clindamycin	SRP + DOX	1	23	-1.00 [-1.32, -0.68]				
6.	SRP + Clindamycin	SRP + MET	1	26	0.30 [-0.00, 0.60]				
7.	SRP + DOX	SRP + MET	1	27	1.30 [1.00, 1.60]				
8.	SRP + AMOX + MET	SRP + Placebo/alone	5	682	-0.42 [-0.52, -0.31]	71.4%	None	None	Borges et al. (2017) – Arm A, B & C
**Systemic antibiotics: Short-term PPD reduction**									
1.	SRP + MINO HCl	SRP + Placebo/alone	2	80	-0.18 [-0.42, 0.06]	0%			
2.	SRP + AZ	SRP + Placebo/alone	10	285	-0.14 [-0.43, 0.15]	95.7%	Emingil et al. (2012) at 3-months<br>Gomi et al. (2007)	Emingil et al. (2012) at 3-months<br>Gomi et al. (2007)	Emingil et al. (2012) at 3-months<br>Gomi et al. (2007)
3.	SRP + Placebo/alone	SRP + SZ	2	120	-1.62 [-2.08, -1.15]	0%			
4.	SRP + AZ	SRP + AMOX + MET	2	45	0.18 [-0.33, 0.68]	84.3%	None	None	Saleh et al. (2016)
5.	SRP + AMOX + MET	SRP + Placebo/alone	12	466	-0.56 [-0.81, -0.32]	97.9%	Ardila et al. (2020)<br>Dukić et al. (2016)<br>Silva et al. (2011)	None	Ardila et al. (2020)<br>Sillva et al. (2011)
6.	SRP + CLM	SRP + Placebo/alone	2	70	-0.57 [-1.49, 0.35]	98.9%	None	None	Suryaprasanna et al. (2018)

No.	Comparisons		m	o	MD [95% CI] (IV, Random)	I^2	Outliers	Influential cases	Large overall heterogeneity contribution
	Treatment 1	Treatment 2							
7.	SRP + MOX	SRP + Placebo/alone	2	64	-0.70 [-1.16, -0.25]	95.6%	None	None	Ardila et al. (2020)
8.	SRP + MOX	SRP + AMOX + MET	1	24	0.09 [-0.07, 0.25]				
9.	SRP + SEC	SRP + Placebo/alone	1	50	-0.20 [-0.39, -0.01]				
10.	SRP + MET	SRP + AMOX + MET	2	56	0.13 [-0.24, 0.49]	71.8%	None	None	Silva et al. (2011)
11.	SRP + MET	SRP + Placebo/alone	2	57	-0.80 [-1.95, 0.34]	97.9%	None	None	Silva et al. (2011)
12.	SRP + DOX	SRP + AMOX + MET	1	60	0.43 [0.16, 0.70]				
13.	SRP + DOX	SRP + MET	1	22	0.48 [0.27, 0.69]				
14.	SRP + DOX	SRP + Placebo/alone	1	21	0.26 [0.02, 0.50]				
15.	SRP + CEF	SRP + AMOX + MET	1	60	-0.01 [-0.03, 0.01]				
16.	SRP + CEF	SRP + Placebo/alone	1	60	-0.04 [-0.06, -0.02]				
**Systemic antibiotics: Intermediate-term PPD reduction**									
1.	SRP + CLM	SRP + Placebo/alone	3	106	-0.06 [-0.38, 0.26]	87.0%	None	Bechara Andere et al. (2018) Suryaprasanna et al. (2018)	Bechara Andere et al. (2018) Suryaprasanna et al. (2018)
2.	SRP + MOX	SRP + Placebo/alone	2	64	-0.65 [-1.25, -0.05]	97.3%	None	None	Ardila et al. (2020)
3.	SRP + AMOX + MET	SRP + Placebo/alone	9	334	-0.65 [-0.80, -0.50]	88.7%	Ardila et al. (2020) Ribeiro et al. (2009)	None	Ardila et al. (2020)
4.	SRP + MOX	SRP + AMOX + MET	1	24	0.07 [-0.09, 0.23]				
5.	SRP + MINO HCl	SRP + Placebo/alone	1	40	-0.20 [-0.60, 0.20]				
6.	SRP + AZ	SRP + Placebo/alone	9	294	-0.14 [-0.36, 0.08]	91.5%	Pradeep et al. (2013)	Gomi et al. (2007)	Pradeep et al. (2013) Gomi et al. (2007)
7.	SRP + SZ	SRP + Placebo/alone	1	60	-2.42 [-3.01, -1.83]				
8.	SRP + Clindamycin	SRP + Placebo/alone	1	21	-0.90 [-1.25, -0.55]				
9.	SRP + DOX	SRP + Placebo/alone	2	43	-0.24 [-0.44, -0.05]	0%			
10.	SRP + MET	SRP + Placebo/alone	2	48	-1.04 [-1.30, -0.79]	48.3%			
11.	SRP + Clindamycin	SRP + DOX	1	23	-0.60 [-0.91, -0.29]				
12.	SRP + Clindamycin	SRP + MET	1	26	0.00 [-0.29, 0.29]				
13.	SRP + DOX	SRP + MET	2	49	0.78 [0.43, 1.13]	79.2%	None	None	Xajigeorgiou et al. (2006)
14.	SRP + DOX	SRP + AMOX + MET	1	20	0.62 [0.32, 0.92]				

No.	Comparisons		m	o	MD [95% CI] (IV, Random)	I²	Outliers	Influential cases	Large overall heterogeneity contribution
	Treatment 1	Treatment 2							
15.	SRP + MET	SRP + AMOX + MET	1	22	-0.34 [-0.61, -0.07]				
**Systemic antibiotics: Long-term PPD reduction**									
1.	SRP + AZ	SRP + Placebo/alone	4	136	-0.15 [-0.51, 0.21]	53.9%	None	None	Haas et al. (2008)
2.	SRP + Clindamycin	SRP + Placebo/alone	1	26	-1.60 [-1.91, -1.29]				
3.	SRP + DOX	SRP + Placebo/alone	1	22	-0.60 [-0.89 -0.31]				
4.	SRP + MET	SRP + Placebo/alone	1	25	-1.90 [-2.14, -1.66]				
5.	SRP + Clindamycin	SRP + DOX	1	23	-1.00 [-1.31, -0.69]				
6.	SRP + Clindamycin	SRP + MET	1	26	0.30 [0.03, 0.57]				
7.	SRP + DOX	SRP + MET	1	27	1.30 [1.06, 1.54]				
8.	SRP + AMOX + MET	SRP + Placebo/alone	5	682	-0.49 [-0.58, - 0.40]	79.2%	Harks et al. (2014)	None	Borges et al. (2017) – Arm D
**Systemic antibiotics: Short-term BOP reduction**									
1.	SRP + CLM	SRP + Placebo/alone	1	40	-2.00 [-5.58, 1.58]				
2.	SRP + MOX	SRP + Placebo/alone	2	64	-0.82 [-1.83, 0.18]	0%			
3.	SRP +MOX	SRP + AMOX + MET	1	24	0.60 [-0.55, 1.75]				
4.	SRP + AMOX + MET	SRP + Placebo/alone	9	328	-8.17 [-12.85, -3.49]	94.6%	Ardila et al. (2020) Borges et al. (2017) – Arm D	Ardila et al. (2020)	Borges et al. (2017) – Arm C & D
5.	SRP + AZ	SRP + Placebo/alone	8	228	-4.26 [-6.47, -2.05]	33.0%			
6.	SRP + AZ	SRP + AMOX + MET	2	45	4.35 [-1.92; 10.62]	18.7%			
7.	SRP + MET	SRP + AMOX + MET	1	34	-16.20 [-21.11, -11.29]				
8.	SRP + MET	SRP + Placebo/alone	1	34	-19.50 [-24.67, -14.34]				
**Systemic antibiotics: Intermediate-term BOP reduction**									
1.	SRP + CLM	SRP + Placebo/alone	2	76	-2.01 [-5.63, 1.60]	42.5%			
2.	SRP + MOX	SRP + Placebo/alone	2	64	0.47 [-2.76, 3.70]	88.5%	None	None	Ardila et al. (2015)
3.	SRP + AMOX + MET	SRP + Placebo/alone	5	200	-8.52 [-13.92, -3.12]	96.4%	Ardila et al. (2020)	Ardila et al. (2020)	Ardila et al. (2020) Borges et al. (2017) – Arm D
4.	SRP + MOX	SRP + AMOX + MET	1	24	0.40 [-0.73, 1.53]				
5.	SRP + AZ	SRP + Placebo/alone	6	197	-3.80 [-6.61, -0.99]	39.9%			

No.	Comparisons		m	o	MD [95% CI] (IV, Random)	I²	Outliers	Influential cases	Large overall heterogeneity contribution
	Treatment 1	Treatment 2							
**Systemic antibiotics: Long-term BOP reduction**									
1.	SRP + AZ	SRP + Placebo/alone	4	136	2.58 [-3.15, 8.30]	56.7%	None	None	Morales et al. (2021)
2.	SRP + AMOX + MET	SRP + Placebo/alone	4	176	-14.28 [-16.59, -11.97]	58.0%	None	Borges et al. (2017) – Arm B	Borges et al. (2017) – Arm B & C
**Local antibiotics: Short-term CAL gain**									
1.	SRP + TET fibre	SRP + Placebo/alone	3	140	0.68 [0.57, 0.79]	0%			
2.	SRP + MINO microsphere	SRP + MET gel	2	80	-0.60 [-0.83, -0.37]	0%			
3.	SRP + MET gel	SRP + Placebo/alone	2	80	0.35 [0.11, 0.60]	0%			
4.	SRP + MINO microsphere	SRP + Placebo/alone	2	80	-0.95 [-1.17, -0.73]	0%			
**Local antibiotics: Intermediate-term CAL gain**									
1.	SRP + MET gel	SRP + Placebo/alone	2	318	-0.64 [-1.76, 0.48]	99.9%	None	None	Stelzel & Flores-de-Jacoby (2000)
**Local antibiotics: Short-term PPD reduction**									
1.	SRP + MET gel	SRP + Placebo/alone	3	100	-0.45 [-1.35, 0.45]	98.7%	None	Mahmood et al. (2019)	Mahmood et al. (2019)
2.	SRP + TET fibre	SRP + Placebo/alone	3	140	-0.62 [-0.87, -0.37]	86.8%	None	None	Dang et al. (2016)
3.	SRP + MINO microsphere	SRP + Placebo/alone	4	178	-0.96 [-1.35, -0.56]	94.7%	None	Pandit et al. (2013) Querido et al. (2004)	Pandit et al. (2013) Querido et al. (2004)
4.	SRP + MINO microsphere	SRP + MET gel	2	80	-0.40 [-0.52, -0.28]	0%			
**Local antibiotics: Intermediate-term PPD reduction**									
1.	SRP + MET gel	SRP + Placebo/alone	2	318	-0.50 [-1.13, 0.13]	99.8%	None	None	Stelzel & Flores-de-Jacoby (2000)
2.	SRP + MINO microsphere	SRP + Placebo/alone	2	52	-0.74 [-1.02, -0.47]	29.8%			
**Local antibiotics: Long-term PPD reduction**									
1.	SRP + MINO microsphere	SRP + Placebo/alone	2	52	-0.79 [-1.35, -0.22]	75.6%	None	None	Cortelli et al. (2006) at 24-months
**Local antibiotics: Intermediate-term BOP reduction**									
1.	SRP + MET gel	SRP + Placebo/alone	1	118	8.00 [6.20, 9.80]				
**Antimicrobial Peptides: Short-term CAL gain**									
1.	SRP + AMP	SRP + Placebo/alone	2	318	-0.18 [-0.61, 0.25]	0%			
**Antimicrobial Peptides: Short-term PPD reduction**									
1.	SRP + AMP	SRP + Placebo/alone	2	318	-0.10 [-0.52, 0.32]	0%			

No.	Comparisons		m	o	MD [95% CI] (IV, Random)	I^2	Outliers	Influential cases	Large overall heterogeneity contribution
	Treatment 1	Treatment 2							
**Antimicrobial Peptides: Short-term BOP reduction**									
1.	SRP + AMP	SRP + Placebo/alone	2	318	-9.15 [-22.37, 4.08]	0%			

Short-term CAL gain

	**Direct comparison**									
**SZ**	NA	NA	NA	NA	NA	NA	NA	NA	NA	-1.87 (-2.43 to -1.30)
-1.22 (-1.86 to -0.59)	**MOX**	-0.09 (-0.56 to 0.38))	NA	NA	NA	NA	NA	NA	NA	-0.75 (-1.06 to -0.44)
-1.57 (-2.15 to -0.99)	-0.35 (-0.66 to -0.04)	**AM**	NA	NA	-0.04 (-0.48 to 0.40)	-0.05 (-0.46 to 0.35)	NA	-0.06 (-0.72 to 0.60)	-0.06 (-0.44 to 0.32)	-0.29 (-0.43 to -0.15)
-1.65 (-2.29 to -1.00)	-0.42 (-0.85 to 0.01)	-0.07 (-0.41 to 0.26)	**CLM**	NA	NA	NA	NA	NA	NA	-0.22 (-0.53 to 0.09)
-1.67 (-2.40 to -0.93)	-0.44 (-1.00 to 0.12)	-0.09 (-0.58 to 0.40)	-0.02 (-0.58 to 0.54)	**SEC**	NA	NA	NA	NA	NA	-0.20 (-0.67 to 0.27)
-1.68 (-2.36 to -0.99)	-0.45 (-0.94 to 0.03)	-0.11 (-0.49 to 0.28)	-0.03 (-0.53 to 0.46)	-0.01 (-0.62 to 0.60)	**CEF**	NA	NA	NA	NA	-0.12 (-0.56 to 0.32)
-1.74 (-2.33 to -1.16)	-0.52 (-0.85 to -0.19)	-0.17 (-0.37 to 0.03)	-0.10 (-0.44 to 0.25)	-0.08 (-0.57 to 0.42)	-0.06 (-0.48 to 0.35)	**AZ**	NA	NA	NA	-0.10 (-0.26 to 0.05)
-1.77 (-2.44 to -1.10)	-0.55 (-1.01 to -0.08)	-0.20 (-0.59 to 0.19)	-0.12 (-0.60 to 0.35)	-0.11 (-0.70 to 0.49)	-0.09 (-0.62 to 0.44)	-0.03 (-0.42 to 0.37)	**MINO hcl**	NA	NA	-0.09 (-0.46 to 0.27)
-1.90 (-2.71 to -1.09)	-0.67 (-1.32 to -0.02)	-0.33 (-0.91 to 0.26)	-0.25 (-0.91 to 0.41)	-0.23 (-0.98 to 0.52)	-0.22 (-0.91 to 0.48)	-0.15 (-0.76 to 0.45)	-0.13 (-0.81 to 0.56)	**DOX**	0.14 (-0.53 to 0.81)	0.15 (-0.48 to 0.78)
-1.87 (-2.52 to -1.21)	-0.64 (-1.08 to -0.20)	-0.29 (-0.63 to 0.04)	-0.22 (-0.67 to 0.23)	-0.20 (-0.78 to 0.37)	-0.19 (-0.69 to 0.31)	-0.12 (-0.49 to 0.24)	-0.10 (-0.59 to 0.39)	0.03 (-0.59 to 0.65)	**MET**	0.19 (-0.17 to 0.55)
-1.87 (-2.43 to -1.30)	-0.64 (-0.94 to -0.35)	-0.29 (-0.43 to -0.15)	-0.22 (-0.53 to 0.09)	-0.20 (-0.67 to 0.27)	-0.19 (-0.57 to 0.20)	-0.12 (-0.27 to 0.03)	-0.09 (-0.46 to 0.27)	0.03 (-0.55 to 0.62)	0.00 (-0.33 to 0.33)	**Placebo/SD alone**
**Indirect comparison**										

highest - lowest ranked (left - right)

Intermediate-term CAL gain

	**Direct comparison**								
**SZ**	NA	NA	NA	NA	NA	NA	NA	NA	-2.07 (-2.88 to -1.26)
-1.10 (-2.00 to -0.20)	**MET**	-0.20 (-0.77 to 0.37)	NA	-0.21 (-0.87 to 0.45)	-0.56 (-1.01 to -0.11)	NA	NA	NA	-0.99 (-1.40 to -0.58)
-1.15 (-2.13 to -0.18)	-0.05 (-0.58 to 0.49)	**Clindamycin**	NA	NA	-0.50 (-1.09 to 0.09)	NA	NA	NA	-1.10 (-1.70 to -0.50)
-1.35 (-2.23 to -0.48)	-0.25 (-0.77 to 0.27)	-0.20 (-0.84 to 0.44)	**MOX**	-0.11 (-0.64to 0.42)	NA	NA	NA	NA	-0.81 (-1.17 to -0.45)
-1.61 (-2.44 to -0.78)	-0.51 (-0.93 to -0.09)	-0.46 (-1.03 to 0.10)	-0.26 (-0.62 to 0.10)	**AM**	-0.11 (-0.87to 0.65)	NA	NA	NA	-0.45 (-0.64 to -0.26)
-1.65 (-2.57 to -0.73)	-0.54 (-0.99 to -0.10)	-0.50 (-1.05 to 0.06)	-0.29 (-0.84 to 0.25)	-0.04 (-0.49 to 0.42)	**DOX**	NA	NA	NA	-0.49 (-0.94 to -0.04)
-1.89 (-2.72 to -1.05)	-0.78 (-1.23 to -0.34)	-0.74 (-1.32 to -0.15)	-0.53 (-0.93 to -0.13)	-0.27 (-0.56 to 0.01)	-0.24 (-0.72 to 0.24)	**AZ**	NA	NA	-0.18 (-0.39 to 0.03)
-1.96 (-2.82 to -1.10)	-0.86 (-1.35 to -0.36)	-0.81 (-1.43 to -0.19)	-0.61 (-1.06 to -0.15)	-0.35 (-0.70 to 0.01)	-0.31 (-0.84 to 0.22)	-0.07 (-0.44 to 0.30)	**CLM**	NA	-0.11 (-0.41 to 0.19)
-2.00 (-2.98 to -1.02)	-0.90 (-1.58 to -0.22)	-0.85 (-1.62 to -0.07)	-0.65 (-1.30 to 0.00)	-0.39 (-0.97 to 0.20)	-0.35 (-1.05 to 0.35)	-0.11 (-0.70 to 0.48)	-0.04 (-0.67 to 0.59)	**MINO**	-0.07 (-0.62 to 0.48)
-2.07 (-2.88 to -1.26)	-0.97 (-1.36 to -0.57)	-0.92 (-1.46 to -0.38)	-0.72 (-1.06 to -0.38)	-0.46 (-0.64 to -0.27)	-0.42 (-0.85 to 0.01)	-0.18 (-0.39 to 0.03)	-0.11 (-0.41 to 0.19)	-0.07 (-0.62 to 0.48)	**Placebo/SD alone**
**Indirect comparison**									

highest - lowest ranked (left - right)

Long-term CAL gain

	**Direct comparison**				
**MET**	-0.30 (-0.69to 0.09)	-1.30 (-1.69to-0.91)	NA	-1.80 (-2.18to-1.42)	NA
-0.30 (-0.69 to 0.09)	**Clindamycin**	-1.00 (-1.41to-0.59)	NA	-1.50 (-1.90to-1.10)	NA
-1.30 (-1.69 to -0.91)	-1.00 (-1.41to-0.59)	**DOX**	NA	-0.50 (-0.90to-0.10)	NA
-1.37 (-1.77 to -0.97)	-1.07 (-1.49to-0.65)	-0.07 (-0.49to 0.35)	**AM**	-0.43 (-0.56to-0.29)	NA
-1.80 (-2.18 to -1.42)	-1.50 (-1.90to-1.10)	-0.50 (-0.90to-0.10)	-0.43 (-0.56to-0.29)	**Placebo/SD alone**	-0.05 (-0.28to 0.19)
-1.85 (-2.29 to -1.40)	-1.55 (-2.01to-1.08)	-0.55 (-1.01to-0.09)	-0.47 (-0.74to-0.20)	-0.05 (-0.28to 0.19)	**AZ**
**Indirect comparison**					

highest - lowest ranked (left - right)

Figure S1: League table of pairwise comparisons in the NMA for CAL gain following the adjunctive used of systemic antibiotics

Short-term PPD reduction

	Direct comparison									
**SZ**	NA	NA	NA	NA	NA	NA	NA	NA	NA	-1.66 (-2.41 to -0.91)
-1.05 (-1.97 to -0.12)	**MET**	NA	0.13 (-0.48 to 0.74)	NA	NA	NA	NA	NA	-0.48 (-1.33 to 0.37)	-0.80 (-1.40 to -0.19)
-1.05 (-1.98 to -0.12)	-0.01 (-0.76 to 0.75)	**MOX**	0.09 (-0.74t o 0.92)	NA	NA	NA	NA	NA	.	-0.71 (-1.29 to -0.12)
-1.11 (-1.89 to -0.32)	-0.06 (-0.60 to 0.48)	-0.05 (-0.62 to 0.51)	**AM**	NA	0.01 (-0.81 to 0.83)	NA	NA	-0.19 (-0.80 to 0.42)	-0.43 (-1.29 to 0.43)	-0.56 (-0.81 to -0.32)
-1.09 (-2.04 to -0.14)	-0.05 (-0.84 to 0.75)	-0.04 (-0.84 to 0.76)	0.02 (-0.62 to 0.65)	**CLM**	NA	NA	NA	NA	NA	-0.56 (-1.15 to 0.02)
-1.36 (-2.40 to -0.32)	-0.31 (-1.19 to 0.57)	-0.30 (-1.20 to 0.59)	-0.25 (-0.97 to 0.47)	-0.26 (-1.19 to 0.66)	**CEF**	NA	NA	NA	NA	-0.04 (-0.86 to 0.78)
-1.46 (-2.58 to -0.33)	-0.41 (-1.41 to 0.59)	-0.40 (-1.41 to 0.60)	-0.35 (-1.22 to 0.52)	-0.36 (-1.39 to 0.66)	-0.10 (-1.21 to 1.01)	**SEC**	NA	NA	NA	-0.20 (-1.04 to 0.64)
-1.47 (-2.45 to -0.49)	-0.43 (-1.25 to 0.40)	-0.42 (-1.25 to 0.41)	-0.36 (-1.03 to 0.31)	-0.38 (-1.24 to 0.48)	-0.11 (-1.07 to 0.84)	-0.02 (-1.06 to 1.03)	**MINO hcl**	NA	NA	-0.18 (-0.81 to 0.44)
-1.49 (-2.28 to -0.69)	-0.44 (-1.03 to 0.15)	-0.43 (-1.04 to 0.17)	-0.38 (-0.71 to -0.05)	-0.39 (-1.04 to 0.25)	-0.13 (-0.89 to 0.63)	-0.03 (-0.91 to 0.85)	-0.02 (-0.70 to 0.66)	**AZ**	NA	-0.14 (-0.41 to 0.13)
-1.66 (-2.71 to -0.61)	-0.61 (-1.39 to 0.16)	-0.61 (-1.51 to 0.30)	-0.55 (-1.28 to 0.18)	-0.57 (-1.50 to 0.37)	-0.30 (-1.31 to 0.71)	-0.20 (-1.32 to 0.91)	-0.19 (-1.15 to 0.77)	-0.17 (-0.94 to 0.60)	**DOX**	0.26 (-0.59t o 1.11)
-1.66 (-2.41 to -0.91)	-0.61 (-1.15 to -0.07)	-0.60 (-1.15 to -0.06)	-0.55 (-0.79 to -0.31)	-0.56 (-1.15 to 0.02)	-0.30 (-1.02 to 0.42)	-0.20 (-1.04 to 0.64)	-0.18 (-0.81 to 0.44)	-0.17 (-0.43 to 0.09)	0.00 (-0.73 to 0.73)	**Placebo/SD alone**
Indirect comparison										

highest - lowest ranked (left - right)

Intermediate-term PPD reduction

	Direct comparison								
**SZ**	.	.	.	.	.	.	.	.	-2.42 (-3.18 to -1.66)
-1.43 (-2.26 to -0.60)	**MET**	-0.00 (-0.55 to 0.55)	-0.34 (-0.89 to 0.21)	.	-0.78 (-1.15 to -0.41)	.	.	.	-1.04 (-1.41 to -0.66)
-1.52 (-2.44 to -0.60)	-0.09 (-0.60 to 0.43)	**Clindamycin**	.	.	-0.60 (-1.16 to -0.04)	.	.	.	-0.90 (-1.49 to -0.31)
-1.77 (-2.55 to -1.00)	-0.34 (-0.71 to 0.03)	-0.26 (-0.79 to 0.28)	**AM**	-0.07 (-0.57 to 0.43)	-0.62 (-1.18 to -0.06)	.	.	.	-0.66 (-0.84 to -0.49)
-1.84 (-2.66 to -1.02)	-0.41 (-0.89 to 0.06)	-0.32 (-0.93 to 0.29)	-0.07 (-0.41 to 0.28)	**MOX**	.	.	.	.	-0.64 (-0.99 to -0.30)
-2.21 (-3.05 to -1.37)	-0.78 (-1.15 to -0.41)	-0.69 (-1.21 to -0.17)	-0.44 (-0.81 to -0.06)	-0.37 (-0.85 to 0.11)	**DOX**	.	.	.	-0.25 (-0.64 to 0.14)
-2.22 (-3.20 to -1.24)	-0.79 (-1.50 to -0.08)	-0.70 (-1.51 to 0.10)	-0.45 (-1.09 to 0.19)	-0.38 (-1.08 to 0.32)	-0.01 (-0.73 to 0.70)	**MINO**	.	.	-0.20 (-0.82 to 0.42)
-2.27 (-3.05 to -1.49)	-0.84 (-1.24 to -0.44)	-0.75 (-1.31 to -0.20)	-0.50 (-0.75 to -0.25)	-0.43 (-0.80 to -0.06)	-0.06 (-0.47 to 0.34)	-0.05 (-0.69 to 0.59)	**AZ**	.	-0.15 (-0.33 to 0.03)
-2.36 (-3.17 to -1.55)	-0.93 (-1.39 to -0.47)	-0.84 (-1.44 to -0.24)	-0.59 (-0.93 to -0.25)	-0.52 (-0.96 to -0.08)	-0.15 (-0.62 to 0.31)	-0.14 (-0.82 to 0.54)	-0.09 (-0.44 to 0.26)	**CLM**	-0.06 (-0.36 to 0.24)
-2.42 (-3.18 to -1.66)	-0.99 (-1.34 to -0.64)	-0.90 (-1.42 to -0.38)	-0.65 (-0.82 to -0.48)	-0.58 (-0.90 to -0.25)	-0.21 (-0.57 to 0.15)	-0.20 (-0.82 to 0.42)	-0.15 (-0.33 to 0.03)	-0.06 (-0.36 to 0.24)	**Placebo/SD alone**
Indirect comparison									

highest - lowest ranked (left - right)

Long-term PPD reduction

	Direct comparison				
**MET**	-0.30 (-0.62 to 0.02)	-1.30 (-1.60 to -1.00)	NA	NA	-1.90 (-2.20 to -1.60)
-0.30 (-0.62 to 0.02)	**Clindamycin**	-1.00 (-1.36 to -0.64)	NA	NA	-1.60 (-1.96 to -1.24)
-1.30 (-1.60 to -1.00)	-1.00 (-1.36 to -0.64)	**DOX**	NA	NA	-0.60 (-0.94 to -0.26)
-1.41 (-1.72 to -1.09)	-1.11 (-1.48 to -0.74)	-0.11 (-0.46 to 0.24)	**AM**	NA	-0.49 (-0.58 to -0.40)
-1.84 (-2.22 to -1.46)	-1.54 (-1.97 to -1.11)	-0.54 (-0.95 to -0.13)	-0.43 (-0.68 to -0.18)	**AZ**	-0.06 (-0.30 to 0.17)
-1.90 (-2.20 to -1.60)	-1.60 (-1.96 to -1.24)	-0.60 (-0.94 to -0.26)	-0.49 (-0.58 to -0.40)	-0.06 (-0.30 to 0.17)	**Placebo/SD alone**
Indirect comparison					

highest - lowest ranked (left - right)

Figure S2: League table of pairwise comparisons in the NMA for PPD reduction following the adjunctive used of systemic antibiotics

Short-term BOP reduction

	Direct comparison				
**MET**	-16.20 (-27.05 to -5.35)	NA	NA	NA	-19.50 (-30.46 to -8.54)
-13.74 (-23.32 to -4.16)	**AM**	-5.24 (-14.33 to 3.86)	-0.60 (-10.34 to 9.14)	NA	-8.17 (-11.86 to -4.48)
-18.39 (-28.74 to -8.04)	-4.65 (-9.77 to 0.48)	**AZ**	NA	NA	-3.36 (-7.61 to 0.89)
-19.38 (-30.80 to -7.96)	-5.64 (-12.54 to 1.26)	-0.99 (-8.62 to 6.63)	**MOX**	NA	-0.70 (-7.63 to 6.22)
-20.06 (-34.15 to -5.98)	-6.32 (-17.24 to 4.59)	-1.68 (-12.78 to 9.43)	-0.68 (-12.87 to 11.50)	**CLM**	-2.00 (-12.31 to 8.31)
-22.06 (-31.66 to -12.47)	-8.32 (-11.90 to -4.74)	-3.68 (-7.80 to 0.44)	-2.68 (-9.18 to 3.81)	-2.00 (-12.31 to 8.31)	**Placebo/SD alone**
Indirect comparison					

highest - lowest ranked (left - right)

Intermediate-term BOP reduction

	Direct comparison			
**AM**	NA	NA	-0.40 (-9.90 to 9.10)	-8.48 (-12.86 to -4.10)
-4.92 (-11.38 to 1.54)	**AZ**	NA	NA	-3.16 (-7.97 to 1.65)
-6.19 (-14.60 to 2.22)	-1.27 (-9.94 to 7.40)	**CLM**	NA	-1.89 (-9.11 to 5.32)
-6.48 (-13.45 to 0.49)	-1.56 (-9.55 to 6.43)	-0.29 (-9.92 to 9.34)	**MOX**	0.53 (-6.23 to 7.29)
-8.08 (-12.40 to -3.77)	-3.16 (-7.97 to 1.65)	-1.89 (-9.11 to 5.32)	-1.60 (-7.98 to 4.78)	**Placebo/SD alone**
Indirect comparison				

highest - lowest ranked (left - right)

Long-term BOP reduction

	Direct comparison	
**AM**	-14.27 (-16.99 to -11.55)	.
-14.27 (-16.99 to -11.55)	**Placebo/SD alone**	-3.51 (-7.58 to 0.56)
-17.78 (-22.67 to -12.89)	-3.51 (-7.58 to 0.56)	**AZ**
Indirect comparison		

highest - lowest ranked (left - right)

Figure S3: League table of pairwise comparisons in the NMA for BOP reduction following the adjunctive used of systemic antibiotics

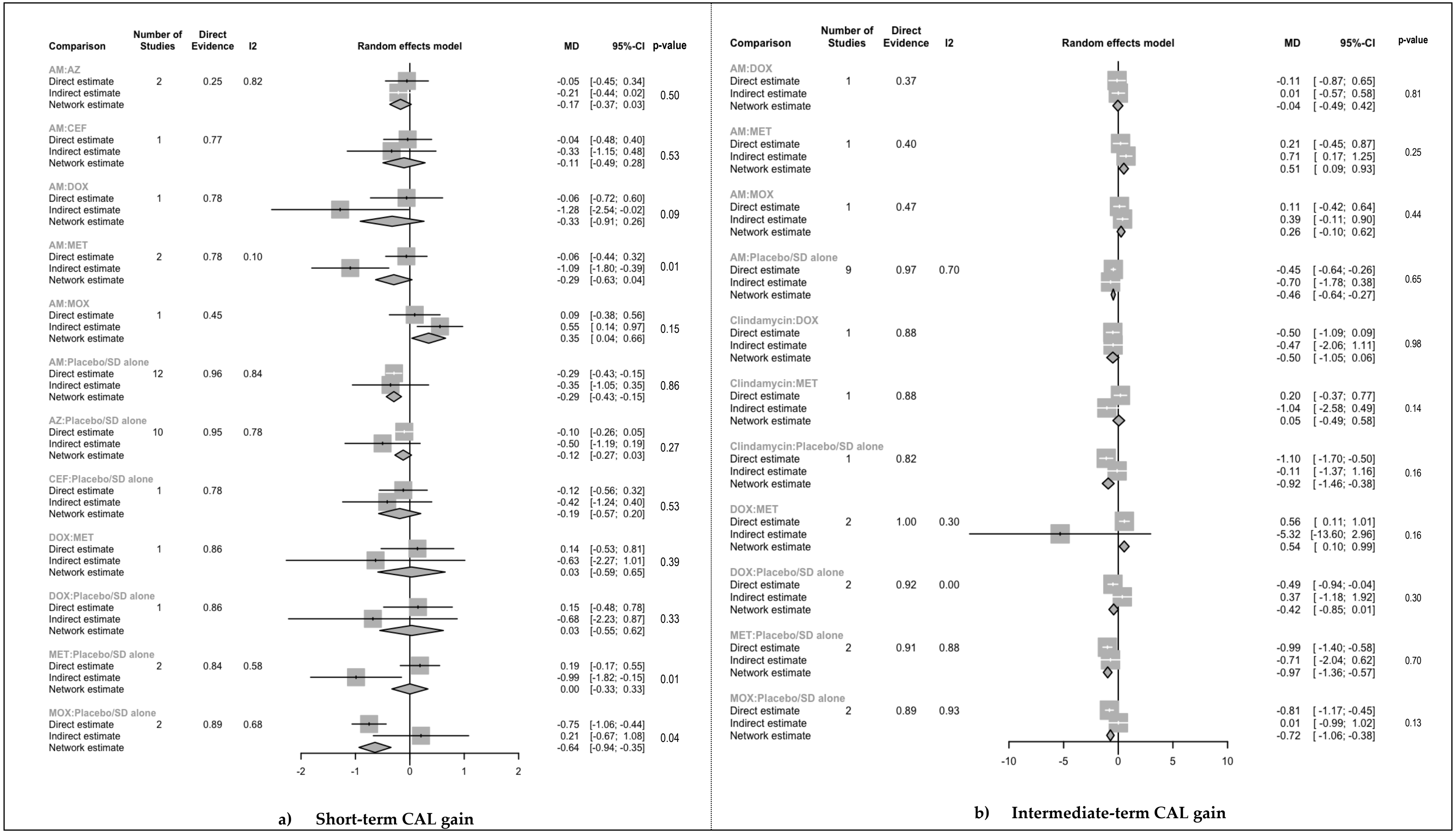

Figure S4. Inconsistency plot for the NMA of CAL gain following adjunctive use of systemic antibiotics

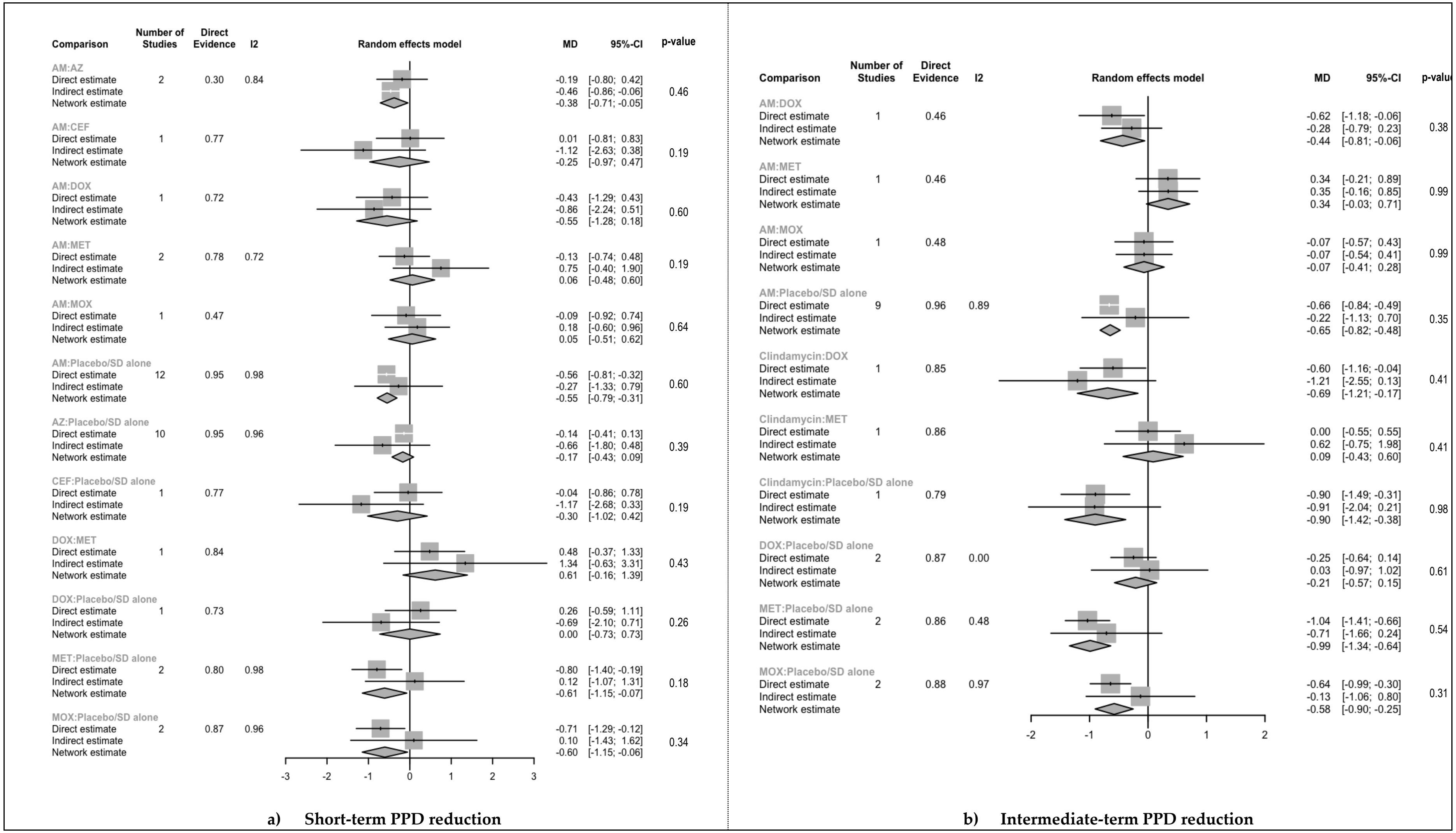

Figure S5. Inconsistency plot for the NMA of PPD reduction following adjunctive use of systemic antibiotics

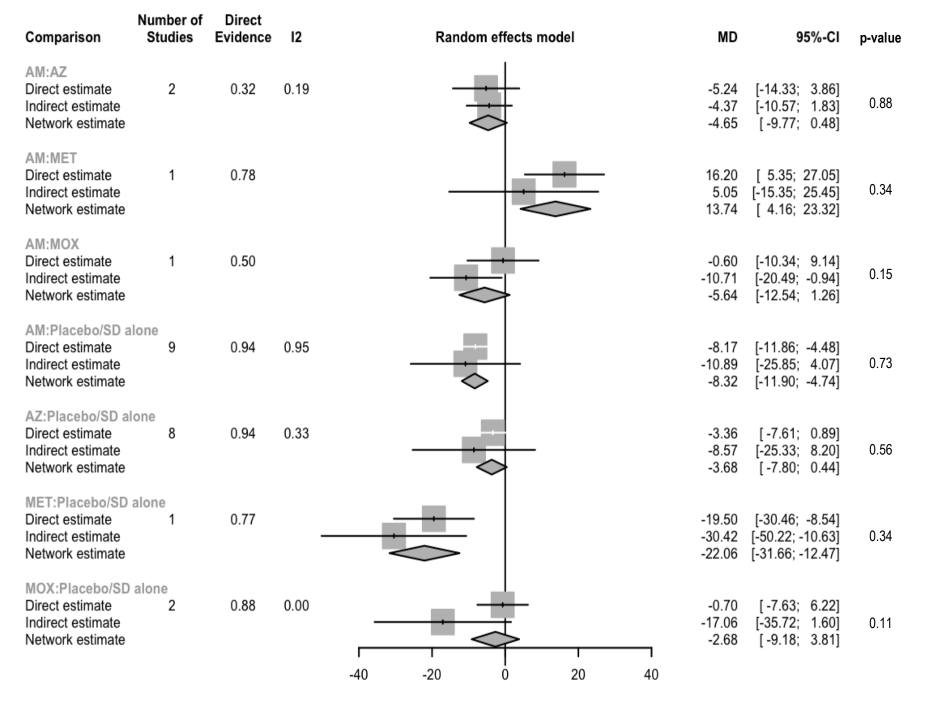

a) Short-term BOP reduction

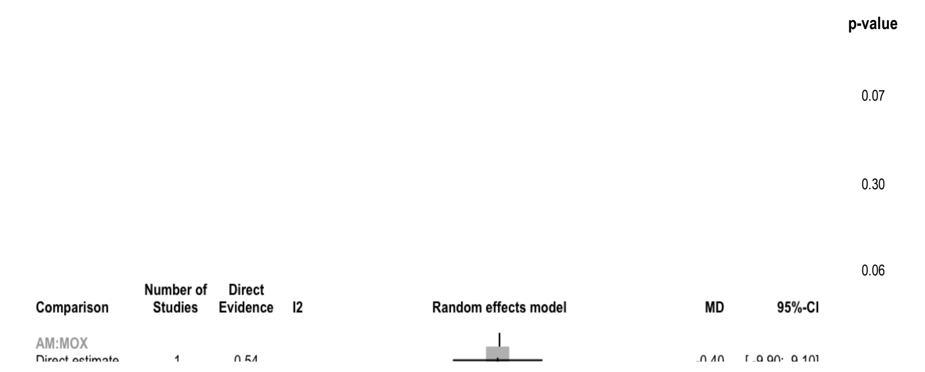

a) Intermediate-term BOP reduction

Figure S6. Inconsistency plot for the NMA of BOP reduction following adjunctive use of systemic antibiotics

Table S12. Subgroup analyses of specific parameters for included studies for CAL gain following adjunctive use of systemic antibiotics

No.	Variables	CAL gain														
		Short-term					Intermediate-term					Long-term				
		No. of studies	MD	95% CI	I^2 (%)	p-value	No. of studies	MD	95% CI	I^2 (%)	p-value	No. of studies	MD	95% CI	I^2 (%)	p-value
**Type of periodontitis**																
1.	Aggressive	18	0.26	-0.11 to 0.41	90.5	<0.0001	14	0.37	0.18 to 0.57	91.2	0.01	1	0.71	(0.02 to 1.40	-	<0.0001
2.	Chronic	16	0.26	0.00 to 0.51	85.1		18	0.41	0.17 to 0.66	88.9		12	0.53	0.11 to 0.96	95.5%	
3.	Severe/advance	1	-0.10	0.58 to 1.22	-		2	0.73	-0.19 to 1.66	81.9						
4.	Moderate-to-advance	6	-0.01	-0.19 to 0.17	44.4		1	0.19	-0.14 to 0.52	-		1	0.30	0.28 to 0.32	-	
5.	Mild-to-moderate	2	0.90	-0.29 to 0.09	0		2	-0.07	-0.26 to 0.13	88.9						
6.	Stage III/IV											1	-0.26	-0.50 to -0.12	-	
**Smokers**																
1.	Include smokers	13	0.03	-0.07 to -.13	24.3	0.05	14	0.15	0.03 to 0.28	53.6	0.00	5	0.11	-0.19 to 0.41	85.1	0.07
2.	Non-smoker	30	0.30	0.13 to 0.45	90.4		23	0.51	0.30 to 0.72	92.1		10	0.63	0.15 to 1.11	96.0	
**Antibiotics initiation**																
1.	After 1st SD	9	-0.03	-0.18 to 0.11	71.6	0.00	3	0.09	-0.18 to 0.36	83.4	0.02					0.53
2.	After SD	22	0.23	0.08 to 0.37	76.5		27	0.36	0.18 to 0.53	86.4		14	0.46	0.08 to 0.84	95.8	
3.	Before SD	6	0.57	0.29 to 0.84	93.7		6	0.66	0.31 to 1.00	93.3		1	0.71	0.02 to 1.40	-	
4.	Unclear	6	0.12	-0.21 to 0.44	84.4		1	0.07	-0.19 to 0.33	-						
**Dosages**																
**AMOX+MET**						<0.0001					<0.0001					<0.0001
1.	375 mg + 250 mg tds for 7 days	1	0.50	0.05 to 0.95	-		3	0.29	0.12 to 0.45	0						
2.	500 mg + 200 mg tds for 7 days	1	0.25	-0.19 to 0.69	-											
3.	500 mg + 250 mg tds for 7 days	2	0.54	0.05 to 1.03	87.8		1	0.40	0.21 to 0.59	-		1	0.50	0.30 to 0.70	-	
4.	500 mg + 400 mg tds for 7 days	1	0.20	-0.02 to 0.42	-		1	0.20	0.18 to 0.62	-		2	0.30	0.28 to 0.32	0	
5.	500 mg + 500 mg tds for 7 days	2	0.34	-0.49 to 1.18	84.7		2	0.70	0.25 to 1.16	79.7						

No.	Variables	CAL gain														
		Short-term					Intermediate-term					Long-term				
		No. of studies	MD	95% CI	I^2 (%)	p-value	No. of studies	MD	95% CI	I^2 (%)	p-value	No. of studies	MD	95% CI	I^2 (%)	p-value
6.	500 mg + 250 mg tds for 14 days	1	0.40	0.23 to 0.57	-		1	0.50	0.33 to 0.67	-		1	0.50	0.34 to 0.66	-	
7.	500 mg + 400 mg tds for 14 days	3	0.14	-0.17 to 0.44	95.0		1	0.40	0.19 to 0.61	-		1	0.50	0.30 to 0.70	-	
8.	500 mg + 500 mg tds for 14 days	1	-0.12	-0.51 to 0.27	-											
**AZ**																
9.	500 mg od for 3 days	9	0.14	-0.06 to 0.33	76.9		7	0.23	-0.11 to 0.56	85.8		3	0.10	-0.53 to 0.73	70.9	
10.	500 mg qid for 5 days	1	-0.20	-0.45 to 0.05	-		2	-0.10	-0.29 to 0.09	0		1	0.00	-0.27 to 0.27	-	
**CEF**																
11.	400 mg od for 7 days	1	0.12	-0.02 to 0.26	-											
**CLM**																
12.	500 mg bd for 3 days	1	0.20	0.09 to 0.31	-		2	-0.01	-0.34 to 0.32	79.4						
13.	500 mg tds for 7 days	1	0.24	0.12 to 0.36	-		1	0.32	0.22 to 0.42	-						
**DOX**																
14.	200 mg od for 8 days						1	0.60	0.27 to 0.93	-		1	0.50	0.19 to 0.81	-	
15.	200 mg then 100 mg od for 14 days	1	-0.15	-0.62 to 0.32)	-		1	0.33	-0.17 to 0.83	-						
**MET**																
16.	400 mg tds for 14 days	1	-0.34	-0.60 to - 0.08	-											
17.	500 mg tds for 7 days	1	-0.01	-0.34 to 0.32	-		1	1.30	1.01 to 1.59	-						
18.	500 mg bd for 8 days						1	0.65	0.31 to 0.99	-		1	1.80	1.51 to 2.09	-	
**MOX**																
19.	400 mg od for 7 days	1	0.66	0.56 to 0.76	-		1	0.62	0.53 to 0.71	-						
20.	400 mg qid for 7 days	1	0.85	0.66 to 1.03	-		1	1.02	0.83 to 1.21	-						

No.	Variables	CAL gain														
		Short-term					Intermediate-term					Long-term				
		No. of studies	MD	95% CI	I^2 (%)	p-value	No. of studies	MD	95% CI	I^2 (%)	p-value	No. of studies	MD	95% CI	I^2 (%)	p-value
**SZ**																
21.	300 mg bd for 10 days	2	1.86	1.38 to 2.34	0		2	1.86	1.38 to 2.33	0						
**SEC**																
22.	1 g of for 30 days	1	0.20	-0.02 to 0.41	-											

Table S13. Subgroup analyses of specific parameters for included studies for PPD reduction following adjunctive use of systemic antibiotics

No.	Variables	PPD reduction														
		Short-term					Intermediate-term					Long-term				
		No. of studies	MD	95% CI	I^2 (%)	p-value	No. of studies	MD	95% CI	I^2 (%)	p-value	No. of studies	MD	95% CI	I^2 (%)	p-value
**Type of periodontitis**																
1.	Aggressive	18	0.22	0.03 to 0.42	96.5	<0.0001	14	0.42	0.15 to 0.70	95.8	0.00	1	1.03	0.19 to 1.87	-	0.04
2.	Chronic	16	0.58	0.27 to 0.89	94.8		18	0.40	0.13 to 0.66	89.8		12	0.60	0.16 to 1.03	96.5	
3.	Severe/advance	1	1.00	0.84 to 1.16	-		2	1.18	0.47 to 1.89	81.9						
4.	Moderate-to-advance	6	0.09	-0.18 to 0.37	82.6		1	0.21	0.06 to 0.36	-		1	0.40	0.39 to 0.41	-	
5.	Mild-to-moderate	2	0.01	-0.13 to 0.15	0		2	-0.03	-0.25 to 0.19	81.4						
6.	Stage III/IV											1	0.03	-0.28 to 0.34	0	
**Smokers**																
1.	Include smokers	16	0.20	-0.09 to 0.49	96.3	0.19	22	0.54	0.30 to 0.78	94.2	0.09	10	0.68	0.19 to 1.17	97.0	0.14
2.	Non-smoker	27	0.42	0.25 to 0.59	97.1		15	0.24	0.00 to 0.48	93.2		5	0.24	-0.07 to 0.55	78.1	
**Antibiotics initiation**																
1.	After 1st SD	9	0.52	0.07 to 0.98	97.5	0.24	3	0.06	-0.16 to 0.29	85.6	0.06					0.30
2.	After SD	22	0.23	0.07 to 0.40	93.7		27	0.44	0.21 to 0.66	92.8		14	0.54	0.16 to 0.92	96.5	
3.	Before SD	6	0.57	0.18 to 0.97	97.8		6	0.55	0.17 to 0.92	96.8		1	1.03	0.19 to 1.87	-	
4.	Unclear	6	0.13	-0.22 to 0.49	85.6		1	0.20	-0.20 to 0.60	-						
**Dosages**																
**AMOX+MET**						<0.0001					<0.0001					<0.0001

No.	Variables	PPD reduction														
		Short-term					Intermediate-term					Long-term				
		No. of studies	MD	95% CI	I^2 (%)	p-value	No. of studies	MD	95% CI	I^2 (%)	p-value	No. of studies	MD	95% CI	I^2 (%)	p-value
1.	375 mg + 250 mg tds for 7 days	1	0.70	0.29 to 1.11	-		3	0.82	0.12 to 1.53	87.9						
2.	500 mg + 200 mg tds for 7 days	1	0.27	-0.16 to 0.70	-											
3.	500 mg + 250 mg tds for 7 days	2	0.58	0.19 to 0.97	84.1		1	0.60	0.48 to 0.72	-		1	0.50	0.38 to 0.62	-	
4.	500 mg + 400 mg tds for 7 days	1	0.50	0.39 to 0.61	-		1	0.60	0.48 to 0.72	-						
5.	500 mg + 500 mg tds for 7 days	2	0.61	-0.24 to 1.45	96.5		2	0.97	0.78 to 1.16	30.9		2	0.49	0.30 to 0.69	91.3	
6.	500 mg + 250 mg tds for 14 days	1	0.40	0.28 to 0.52	-		1	0.40	0.28 to 0.52	-		1	0.50	0.37 to 0.63	-	
7.	500 mg + 400 mg tds for 14 days	3	0.71	-0.29 to 1.70	99.2		1	0.50	0.39 to 0.61	-		1	0.50	0.39 to 0.61	-	
8.	500 mg + 500 mg tds for 14 days	1	0.38	0.16 to 0.60	-											
**AZ**																
9.	500 mg od for 3 days	9	0.17	-0.10 to 0.43	96.2		7	0.22	-0.05 to 0.48	93.0		3	0.39	-0.25 to 1.02	59.6	
10.	500 mg qid for 5 days	1	-0.10	-0.45 to 0.25	-		2	-0.15	-0.38 to 0.08	0		1	-0.10	-0.40 to 0.21	-	
**CEF**																
11.	400 mg od for 7 days	1	0.04	0.02 to 0.06	-											
**CLM**																
12.	500 mg bd for 3 days	1	0.10	0.00 to 0.20	-		2	-0.05	-0.40 to 0.30	81.4						
13.	500 mg tds for 7 days	1	1.04	0.87 to 1.21	-		1	0.27	0.09 to 0.45	-						
**DOX**																
14.	200 mg od for 8 days						1	0.30	0.00 to 0.60	-		1	0.60	0.31 to 0.89	-	
15.	200 mg then 100 mg od for 14 days	1	-0.26	-0.50 to -0.02	-		1	0.20	-0.07 to 0.47	-						
**MET**																
16.	400 mg tds for 14 days	1	1.39	1.13 to 1.65	-											

No.	Variables	PPD reduction														
		Short-term					Intermediate-term					Long-term				
		No. of studies	MD	95% CI	I^2 (%)	p-value	No. of studies	MD	95% CI	I^2 (%)	p-value	No. of studies	MD	95% CI	I^2 (%)	p-value
17.	500 mg tds for 7 days	1	0.22	0.01 to 0.43	-		1	1.16	0.92 to 1.40	-						
18.	500 mg bd for 8 days						1	0.90	0.62 to 1.18	-		1	1.90	1.66 to 2.14		
**MINO HCl**																
19.	200 mg then 100 mg od for 14 days	2	0.18	-0.06 to 0.42	0		1	0.20	-0.20 to 0.60	-						
**MOX**																
20.	400 mg od for 7 days	1	0.48	0.39 to 0.58	-		1	0.35	-0.26 to 0.44	-						
21.	400 mg qid for 7 days	1	0.94	0.78 to 1.10	-		1	0.96	0.79 to 1.13	-						
**SZ**																
22.	300 mg bd for 10 days	2	1.62	1.15 to 2.08	0		1	2.42	1.83 to 1.01	-						
**SEC**																
23.	1 g of for 30 days	1	0.18	-0.06 to 0.42												

Table S14. Subgroup analyses of specific parameters for included studies for BOP reduction following adjunctive use of systemic antibiotics

No.	Variables	BOP reduction									
		Short-term					Intermediate-term				
		No. of studies	MD	95% CI	I^2 (%)	p-value	No. of studies	MD	95% CI	I^2 (%)	p-value
**Type of periodontitis**											
1.	Aggressive	7	-1.64	-2.65 to -0.62	55.1	0.00	7	0.81	-0.62 to 2.24	74.1	<0.0001
2.	Chronic	10	-6.90	-13.34 to -0.45	94.1		7	9.07	6.38 to 11.76	59.7	
3.	Severe/advance	2	-7.96	-10.90 to -5.01	0		1	7.55	4.50 to 10.60	-	
4.	Moderate-to-advance	6	-1.67	-4.58 to 1.24	0		1	0.80	-4.09 to 5.69	-	
5.	Mild-to-moderate										
6.	Stage III/IV										
**Smokers**											
1.	Include smokers	8	-3.43	-10.69 to 3.82	93.0	0.67	5	2.55	0.29 to 4.82	0	0.27

No.	Variables	BOP reduction									
		Short-term					Intermediate-term				
		No. of studies	MD	95% CI	I^2 (%)	p-value	No. of studies	MD	95% CI	I^2 (%)	p-value
2.	Non-smoker	17	-5.14	-7.90 to -2.37	92.2		11	4.72	1.62 to 7.81	94.7	
**Antibiotics initiation**											
1.	After 1st SD	6	-1.72	-10.89 to 7.45	94.9	0.00					0.05
2.	After SD	10	-8.74	-11.87 to -5.60	82.3		10	5.86	2.53 to 9.19	83.4	
3.	Before SD	6	-1.93	-3.78 to -0.08	74.6		6	1.65	-0.91 to 4.21	87.0	
4.	Unclear	3	-3.48	-13.13 to 6.17	44.1						
**Dosages**											
**AMOX+MET**						<0.0001					<0.0001
2.	500 mg + 200 mg tds for 7 days	1	-6.70	-20.88 to 7.48	-						
3.	500 mg + 250 mg tds for 7 days	2	-11.24	-14.28 to -8.20	0		1	-8.40	-11.52 to -5.28	-	
4.	500 mg + 400 mg tds for 7 days	1	-15.40	-18.44 to -12.36	-		1	-13.40	-16.32 to -10.48	-	
5.	500 mg + 500 mg tds for 7 days	1	-1.60	-2.48 to -0.72	-		1	-1.50	-2.36 to 0.64	-	
6.	500 mg + 250 mg tds for 14 days	1	-7.40	-10.28 to -4.52	-		1	-8.90	-11.70 to -6.10	-	
7.	500 mg + 400 mg tds for 14 days	3	-6.55	-14.00 to 0.90	89.6		1	-10.80	-13.75 to -7.85	-	
**AZ**											
9.	500 mg od for 3 days	6	-4.78	-7.13 to -2.44	28.6		4	-4.21	-7.61 to -0.81	55.1	
10.	500 mg qid for 5 days	2	-0.04	-6.86 to 6.79	16.9		2	-0.55	-7.84 to 6.74	0	
**CLM**											
12.	500 mg bd for 3 days	1	-2.00	-5.58 to 1.58			1	0.00	-4.18 to 4.18		
13.	500 mg tds for 7 days						1	-3.70	-7.28 to -0.12		
**MET**											
16.	400 mg tds for 14 days	1	-19.5	-24.67 to 14.34	-						
**MOX**											
20.	400 mg od for 7 days	1	-0.40	-2.25 to 1.45	-		1	2.20	0.35 to 4.05	-	

No.	Variables	BOP reduction									
		Short-term					Intermediate-term				
		No. of studies	MD	95% CI	I^2 (%)	p-value	No. of studies	MD	95% CI	I^2 (%)	p-value
21.	400 mg qid for 7 days	1	-1.00	-2.20 to 0.20			1	-1.10	-2.28 to 0.08	-	

Table S15. Sensitivity analyses for short-term CAL gain following adjunctive use of systemic antibiotics

Interventions	All studies						Studies without outliers and influential cases						Studies without imputed data						Studies without high ROB					
	k	o	MD	95% CI	p-score	I^2 (%)	k	o	MD	95% CI	p-score	I^2 (%)	k	o	MD	95% CI	p-score	I^2 (%)	k	o	MD	95% CI	p-score	I^2 (%)
SZ			-1.87	-2.43 to -1.30	1.00				-1.86	-2.35 to -1.36	1.00				-1.87	-2.44 to -1.29	1.00							
MOX			-0.64	-0.94 to -0.35	0.88				-0.66	-0.87 to -0.45	0.87				-0.65	-0.96 to -0.34	0.87				-0.66	-0.93 to -0.38	0.99	
AMOX+MET			-0.29	-0.43 to -0.15	0.66				-0.26	-0.37 to -0.15	0.62				-0.33	-0.48 to -0.17	0.66				-0.35	-0.50 to 0.20	0.78	
CLM			-0.22	-0.53 to 0.09	0.54				-0.22	-0.37 to -0.06	0.53				-0.22	-0.55 to 0.11	0.51				-0.22	-0.50 to 0.06	0.60	
SEC	28	1,171	-0.20	-0.67 to 0.27	0.49	78.0	22	907	-0.20	-0.48 to 0.08	0.47	42.4	23	993	-0.20	-0.70 to 0.30	0.47	80.3	14	642				82.2
CEF			-0.19	-0.57 to 0.20	0.48				-0.17	-0.38 to 0.04	0.42				-0.20	-0.62 to 0.21	0.48				-0.21	-0.57 to 0.15	0.59	
AZ			-0.12	-0.27 to 0.03	0.40				-0.09	-0.19 to 0.02	0.25				-0.06	-0.26 to 0.14	0.30				0.20	-0.26 to 0.66	0.13	
MINO HCl			-0.09	-0.46 to 0.27	0.36				-0.09	-0.34 to 0.15	0.28										-0.09	-0.44 to 0.25	0.43	
DOX			0.03	-0.55 to 0.62	0.25										0.02	-0.58 to 0.62	0.26							
MET			0.00	-0.33 to 0.33	0.23										-0.02	-0.36 to 0.33	0.25				0.11	-0.31 to 0.53	0.20	
SD alone			0.00		0.19				0.00		0.05				0.00		0.19				0.00		0.28	

Table S16. Sensitivity analyses for intermediate-term CAL gain following adjunctive use of systemic antibiotics

Interventions	All studies						Studies without outliers and influential cases						Studies without imputed data						Studies without high ROB					
	**k**	**o**	**MD**	**95% CI**	**p-score**	**I² (%)**	**k**	**o**	**MD**	**95% CI**	**p-score**	**I² (%)**	**k**	**o**	**MD**	**95% CI**	**p-score**	**I² (%)**	**k**	**o**	**MD**	**95% CI**	**p-score**	**I² (%)**
SZ	25	956	-2.07	-2.88 to -1.26	1.00	81.5%	21	807	-2.07	-2.71 to -1.43	1.00	1.0	22	846	-2.07	-2.89 to -1.25	1.00	82.6	14	551				81.1
MET			-0.97	-1.36 to -0.57	0.82				-1.30	-1.59 to -1.01	0.88				-0.96	-1.37 to -0.56	0.80				-1.30	-1.80 to -0.57	0.97	
Clindamycin			-0.92	-1.46 to -0.38	0.78				-1.10	-1.45 to -0.75	0.79				-0.92	-1.48 to -0.36	0.76				-1.10	-1.63 to -0.57	0.88	
MOX			-0.72	-1.05 to -0.38	0.69				-0.62	-0.72 to -0.52	0.62				-0.72	-1.07 to -0.36	0.65				-0.73	-1.02 to -0.44	0.71	
AMOX+MET			-0.46	-0.64 to -0.27	0.51				-0.39	-0.48 to -0.31	0.45				-0.46	-0.65 to -0.26	0.46				-0.51	-0.71 to -0.31	0.55	
DOX			-0.42	-0.85 to 0.01	0.47				-0.60	-0.93 to -0.27	0.59				-0.42	-0.87 to 0.02	0.42				-0.60	-1.12 to -0.08	0.61	
AZ			-0.15	-0.34 to 0.05	0.25				-0.06	-0.15 to 0.04	0.15				-0.12	-0.36 to 0.11	0.19				0.09	-0.24 to 0.42	0.10	
CLM			-0.11	-0.41 to -.19	0.21				-0.30	-0.39 to -0.20	0.34				-0.11	-0.42 to 0.20	0.18				-0.11	-0.37 to 0.14	0.28	
MINO HCl			-0.07	-0.62 to 0.48	0.19				-0.07	-0.33 to 0.19	0.15										-0.07	-0.55 to 0.41	0.24	
SD alone			0.00		0.08				0.00		0.05				0.00		0.05				0.00		0.16	

Table S17. Sensitivity analyses for long-term CAL gain following adjunctive use of systemic antibiotics

Interventions	All studies						Studies without outliers and influential cases						Studies without imputed data						Studies without high ROB					
	k	o	MD	95% CI	p-score	I^2 (%)	k	o	MD	95% CI	p-score	I^2 (%)	k	o	MD	95% CI	p-score	I^2 (%)	k	o	MD	95% CI	p-score	I^2 (%)
MET	10	866	-1.80	-2.18 to -1.42	0.99	67.5	6	710	-1.80	-2.09 to -1.51	0.99	0	8	804	-1.80	-2.15 to -1.45	0.99	64.2	7	298	-1.80	-2.09 to -1.51	0.99	0
Clindamycin			-1.50	-1.90 to -1.10	0.81				-1.50	-1.81 to -1.19	0.81				-1.50	-1.87 to -0.13	0.81				-1.50	-1.81 to -1.19	0.81	
DOX			-0.50	-0.90 to -0.10	0.52				-0.50	-0.81 to -0.19	0.58				-0.50	-0.87 to -0.13	0.53				-0.50	-0.81 to -0.19	0.51	
AMOX+MET			-0.43	-0.56 to -0.29	0.47				-0.30	0.32 to -0.28	0.42				-0.42	-0.54 to -0.31	0.47				-0.48	-0.58 to -0.39	0.49	
SD alone			0.00		0.13				0.00		0.19				0.00		0.10				0.00		0.10	
AZ			0.05	-0.19 to 0.28	0.07				0.14	-0.04 to 0.32	0.01				0.00	-0.32 to 0.32	0.11				0.00	-0.26 to 0.26	0.10	

Table S18. Sensitivity analyses for short-term PPD reduction following adjunctive use of systemic antibiotics

Interventions	All studies						Studies without outliers and influential cases						Studies without imputed data						Studies without high ROB					
	k	o	MD	95% CI	p-score	I² (%)	k	o	MD	95% CI	p-score	I² (%)	k	o	MD	95% CI	p-score	I² (%)	k	o	MD	95% CI	p-score	I² (%)
SZ			-1.66	-2.41 to -0.91	0.99				-1.62	-2.12 to -1.12	1.00				-1.66	-2.44 to -0.88	0.99							
MET			-0.51	-1.15 to -0.07	0.69				-0.33	-0.62 to -0.04	0.63				-0.63	-1.20 to -0.06	0.67				-0.91	-1.63 to -0.20	0.85	
MOX			-0.60	-1.15 to -0.06	0.68				-0.48	-0.75 to -0.21	0.80				-0.61	-1.19 to -0.04	0.65				-0.65	-1.16 to -0.14	0.68	
AMOX+MET			-0.55	-0.79 to -0.31	0.66				-0.41	-0.52 to -0.30	0.76				-0.59	-0.85 to -0.32	0.65				-0.73	-1.02 to -0.44	0.75	
CLM			-0.56	-1.15 to 0.02	0.65				-0.10	-0.37 to 0.17	0.31				-0.56	-1.19 to 0.06	0.61				-0.56	-1.11 to -0.02	0.61	
CEF	28	1,171	-0.30	-1.02 to 0.42	0.43	97.0	21	866				66.2	23	993	-0.32	-1.08 to 0.44	0.42	97.3	13	552				96.5
SEC			-0.20	-1.04 to 0.64	0.37				-0.20	-0.52 to 0.12	0.45				-0.20	-1.09 to 0.69	0.35							
MINO HCl			-0.18	-0.81 to 0.44	0.34				-0.18	-0.48 to 0.11	0.43										-0.18	-0.77 to 0.40	0.31	
AZ			-0.17	-0.43 to 0.09	0.32				-0.18	-0.30 to -0.06	0.42				-0.18	-0.51 to 0.16	0.30				0.10	-0.73 to 0.93	0.15	
DOX			0.00	-0.73 to 0.73	0.22				0.15	-0.16 to 0.47	0.05				-0.01	-0.79 to 0.76	0.21							
SD alone			0.00		0.15				0.00		0.14				0.00		0.14				0.00		0.15	

Table S19. Sensitivity analyses for intermediate-term PPD reduction following adjunctive use of systemic antibiotics

Interventions	All studies						Studies without outliers and influential cases						Studies without imputed data						Studies without high ROB					
	k	o	MD	95% CI	p-score	I^2 (%)	k	o	MD	95% CI	p-score	I^2 (%)	k	o	MD	95% CI	p-score	I^2 (%)	k	o	MD	95% CI	p-score	I^2 (%)
SZ	25	956	-2.42	-3.18 to -1.66	1.00	89.4	18	692				70.6	22	846	-2.42	-3.20 to -1.64	1.00	90.2	14	551				87.4
MET			-0.99	-1.34 to -0.64	0.84				-0.90	-1.26 to -0.54	0.93				-0.99	-1.36 to -0.62	0.82				-0.90	-1.39 to -0.41	0.90	
Clindamycin			-0.90	-1.42 to -0.38	0.78				-0.90	-1.32 to -0.48	0.93				-0.90	-1.45 to -0.36	0.75				-0.90	-1.43 to -0.37	0.90	
AMOX+MET			-0.65	-0.82 to -0.48	0.64				-0.50	-0.61 to -0.39	0.70				-0.65	-0.84 to -0.47	0.61				-0.60	-0.78 to -0.41	0.70	
MOX			-0.58	-0.90 to -0.25	0.59				-0.35	-0.60 to -0.10	0.53				-0.58	-0.93 to -0.23	0.56				-0.56	-0.84 to -0.28	0.67	
DOX			-0.21	-0.57 to 0.15	0.31				-0.30	-0.67 to 0.07	0.47				-0.21	-0.59 to 0.17	0.30				-0.30	-0.80 to 0.20	0.44	
MINO HCl			-0.20	-0.82 to 0.42	0.30				-0.20	-0.66 to 0.26	0.36										-0.20	-0.77 to 0.37	0.37	
AZ			-0.15	-0.33 to 0.03	0.27				-0.06	-0.18 to 0.05	0.30				-0.11	-0.34 to 0.11	0.22				0.15	-0.20 to 0.50	0.07	
CLM			-0.06	-0.36 to 0.24	0.18				-0.14	-0.48 to 0.20	0.29				-0.06	-0.37 to 0.25	0.17				-0.06	-0.32 to 0.20	0.26	
SD alone			0.00		0.09				0.00		0.08				0.00		0.08				0.00		0.19	

Table S20. Sensitivity analyses for long-term PPD reduction following adjunctive use of systemic antibiotics

Interventions	All studies						Studies without outliers and influential cases						Studies without imputed data						Studies without high ROB					
	k	o	MD	95% CI	p-score	I² (%)	k	o	MD	95% CI	p-score	I² (%)	k	o	MD	95% CI	p-score	I² (%)	k	o	MD	95% CI	p-score	I² (%)
MET			-1.90	-2.20 to -1.60	0.99				-1.90	-2.16 to -1.64	1.00				-1.90	-2.19 to -1.61	0.99			298	-1.90	-2.14 to -1.66	1.00	
Clindamycin			-1.60	-1.96 to -1.24	0.81				-1.60	-1.93 to -1.27	0.80				-1.60	-1.95 to -1.25	0.81				-1.60	-1.91 to -1.29	0.80	
DOX	10	866	-0.60	-0.94 to -0.26	0.54	72.8	8	798	-0.60	-0.90 to -0.30	0.56	43.6	8	804	-0.60	-0.93 to -0.27	0.55	74.8	7		-0.60	-0.89 to -0.31	0.54	0
AMOX+MET			-0.49	-0.58 to -0.40	0.45				-0.45	-0.52 to -0.39	0.44				-0.49	-0.58 to -0.41	0.45				-0.53	-0.58 to -0.47	0.46	
AZ			-0.06	-0.30 to 0.17	0.14				0.02	-0.20 to 0.24	0.09				0.06	-0.27 to 0.39	0.07				0.07	-0.23 to 0.36	0.07	
SD alone			0.00		0.06				0.00		0.11				0.00		0.13				0.00		0.13	

Table S21. Sensitivity analyses for short-term BOP reduction following adjunctive use of systemic antibiotics

Interventions	All studies						Studies without outliers and influential cases						Studies without imputed data						Studies without high ROB					
	k	o	MD	95% CI	p-score	I² (%)	k	o	MD	95% CI	p-score	I² (%)	k	o	MD	95% CI	p-score	I² (%)	k	o	MD	95% CI	p-score	I² (%)
MET			-22.06	-31.66 to -12.47	1.00				-21.4	-26.29 to -15.98	1.00				-22.41	-32.92 to -11.91	1.00				-22.34	-33.03 to -11.65	0.99	
AMOX+MET			-8.32	-11.90 to -4.74	0.76				-6.36	-9.04 to -3.68	0.77				-9.03	-13.42 to -4.81	0.75				-8.89	-13.33 to -4.45	0.72	
AZ	17	644	-3.68	-7.80 to 0.44	0.44	90.3	13	476	-4.42	-6.44 to -2.40	0.58	24.4	14	546	-3.42	-9.67 to 2.82	0.41	92.5	10	424	-2.90	-16.41 to 10.61	0.38	95.4
MOX			-2.68	-9.18 to 3.81	0.36				-0.40	-3.79 to 2.99	0.18				-2.88	-10.12 to 4.35	0.37				-2.84	-10.22 to 4.54	0.38	
CLM			-2.00	-12.31 to 8.31	0.32				-2.00	-6.57 to 2.57	0.35				-2.00	-13.36 to 9.36	0.33				-2.00	-13.56 to 9.56	0.34	
SD alone			0.00		0.12				0.00		0.12				0.00		0.14				0.00		0.19	

Table S22. Sensitivity analyses for intermediate-term BOP reduction following adjunctive use of systemic antibiotics

Interventions	All studies						Studies without outliers and influential cases						Studies without imputed data						Studies without high ROB					
	k	o	MD	95% CI	p-score	I^2 (%)	k	o	MD	95% CI	p-score	I^2 (%)	k	o	MD	95% CI	p-score	I^2 (%)	k	o	MD	95% CI	p-score	I^2 (%)
AMOX+MET	14	525	-8.08	-12.40 to -3.77	0.96	92.1	11	405	-9.38	-11.66 to -7.10	1.00	30.1	12	455	-8.10	-12.61 to -3.58	0.93	93.3	10	393	-8.10	-12.65 to -3.54	0.95	94.7
AZ			-3.16	-7.97 to 1.65	0.56				-4.05	-6.37 to -1.73	0.61				-3.88	-10.53 to 2.77	0.59				-0.41	-10.57 to 9.76	0.36	
CLM			-1.89	-9.11 to 5.32	0.42				-2.05	-5.35 to 1.25	0.35				-1.89	-9.41 to 5.64	0.41				-1.89	-9.47 to 5.14	0.44	
MOX			-1.60	-7.98 to 4.78	0.39										-1.60	-8.29 to 5.09	0.38				-1.60	-8.35 to 5.14	0.44	
SD alone			0.00		0.18				0.00		0.04				0.00		0.19				0.00		0.28	

Table S23. Sensitivity analyses for long-term BOP reduction following adjunctive use of systemic antibiotics

Interventions	All studies						Studies without outliers and influential cases						Studies without imputed data						Studies without high ROB					
	k	o	MD	95% CI	p-score	I^2 (%)	k	o	MD	95% CI	p-score	I^2 (%)	k	o	MD	95% CI	p-score	I^2 (%)	k	o	MD	95% CI	p-score	I^2 (%)
AMOX+MET	8	312	-14.27	-16.99 to -11.55	1.00	57.4	5	190	-14.52	-17.31 to -11.72	1.00	25.3	6	250	-14.27	-17.14 to -11.40	1.00	68.7	6	250	-14.27	-17.14 to -11.40	1.00	68.7
SD alone			0.00		0.48				0.00		0.50				0.00		0.47				0.00		0.47	
AZ			3.51	-0.56 to 7.58	0.02				5.74	2.19 to 9.28	0.00				3.89	-0.96 to 8.73	0.03				3.89	-0.96 to 8.73	0.03	

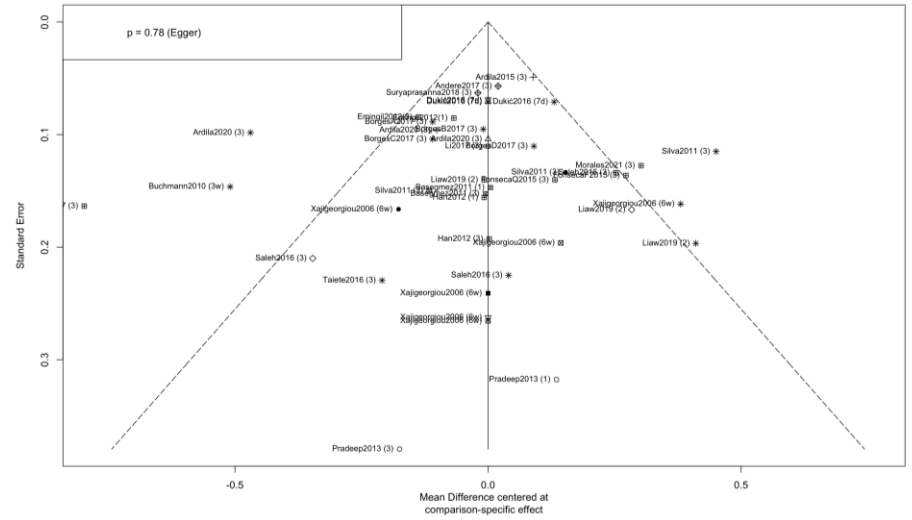

a) Short-term CAL gain

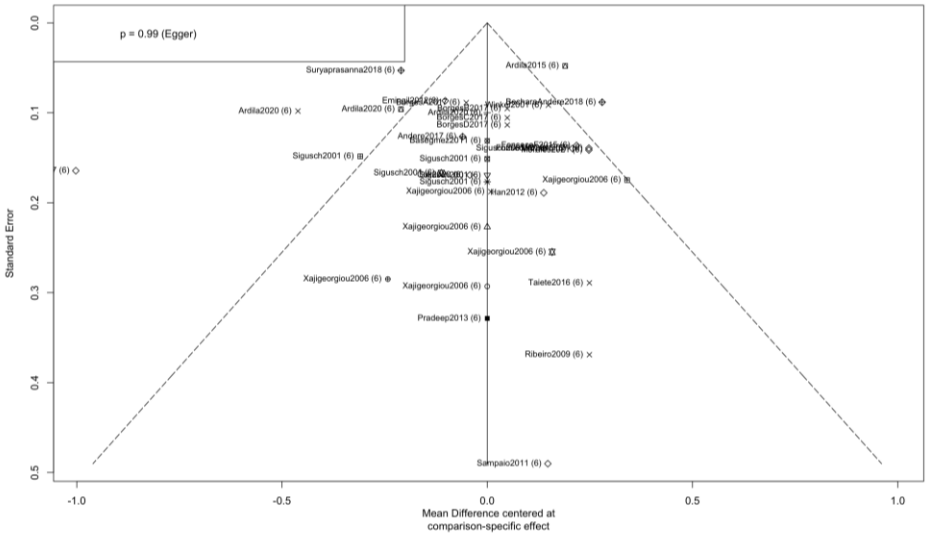

b) Intermediate-term CAL gain

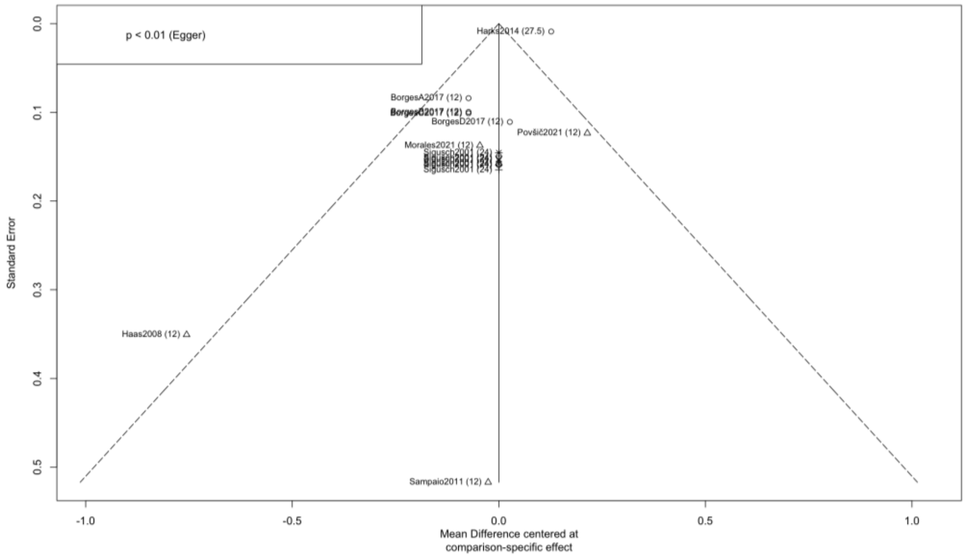

c) Long-term CAL gain

Figure S7. Funnel plots for NMA of CAL gain following adjunctive use of systemic antibiotics

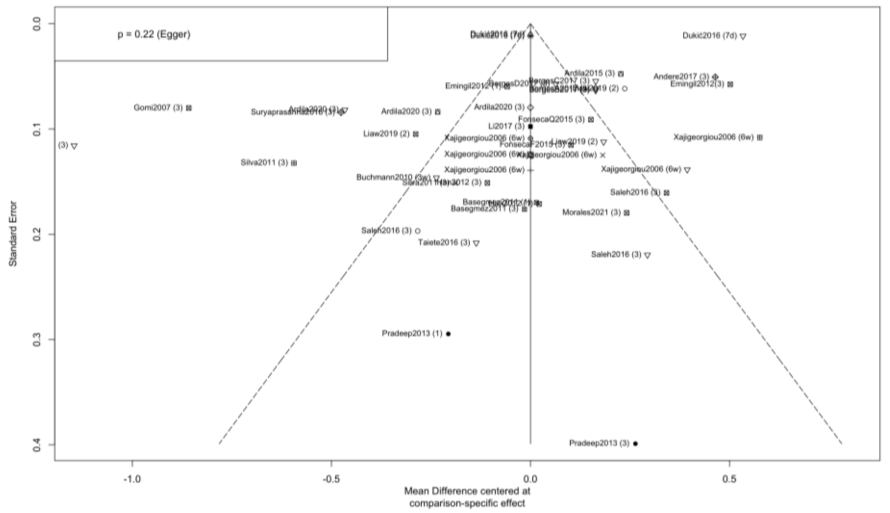

a) **Short-term PPD reduction**

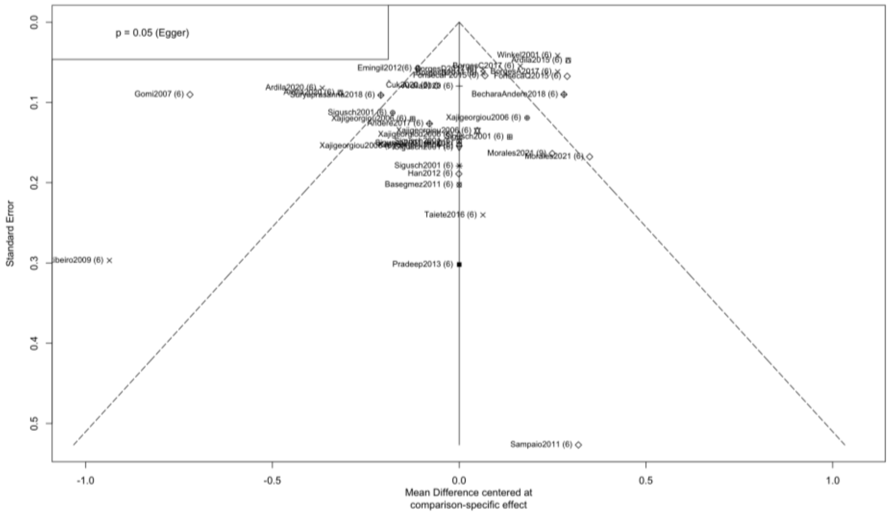

b) **Intermediate-term PPD reduction**

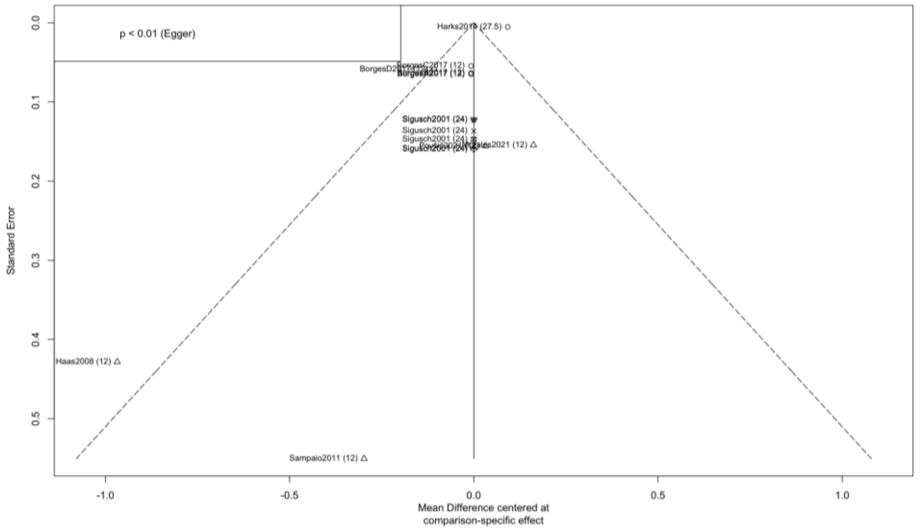

c) **Long-term PPD reduction**

Figure S8. Funnel plots for NMA of PPD reduction following adjunctive use of systemic antibiotics

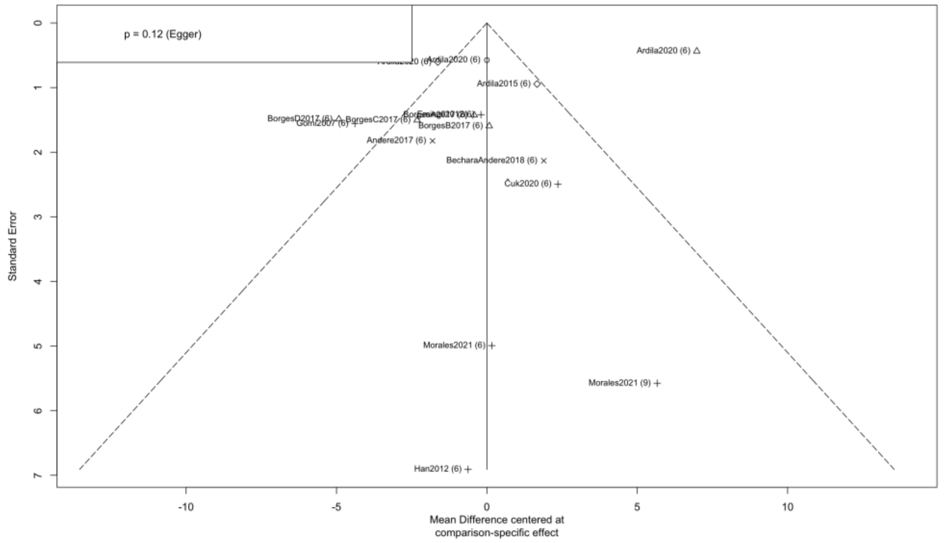

a) Short-term BOP reduction

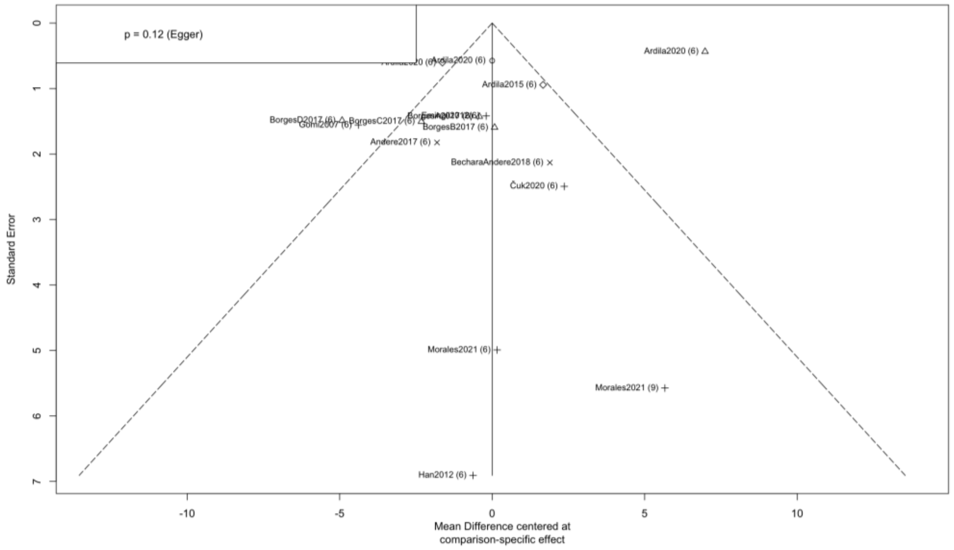

b) Intermediate-term BOP reduction

Figure S9. Funnel plots for NMA of BOP reduction following adjunctive use of systemic antibiotics

Table S24. Certainty of evidence for short-term CAL gain following the use of systemic antibiotics as adjunct to subgingival debridement

Comparisons	GRADE					
	Direct evidence		Indirect evidence		Network meta-analysis	
	MD (95% CI)	Certainty of evidence	MD (95% CI)	Certainty of evidence	MD (95% CI)	Certainty of evidence
**SZ vs.**						
MOX	-	-	-1.22 (-1.86 to -0.59)	⨁⨁◯◯ h<br>Low	-1.22 (-1.86 to -0.59)	⨁⨁◯◯ h<br>Low
AMOX+MET	-	-	-1.57 (-2.15 to -0.99)	⨁◯◯◯ i<br>Very low	-1.57 (-2.15 to -0.99)	⨁◯◯◯ i<br>Very low
CLM	-	-	-1.65 (-2.29 to -1.00)	⨁⨁◯◯ h<br>Low	-1.65 (-2.29 to -1.00)	⨁⨁◯◯ h<br>Low
SEC	-	-	-1.67 (-2.40 to -0.93)	⨁⨁◯◯ h<br>Low	-1.67 (-2.40 to -0.93)	⨁⨁◯◯ h<br>Low
CEF	-	-	-1.68 (-2.36 to -0.99)	⨁⨁◯◯ h<br>Low	-1.68 (-2.36 to -0.99)	⨁⨁◯◯ h<br>Low
AZ	-	-	-1.74 (-2.33 to -1.16)	⨁⨁◯◯ h<br>Low	-1.74 (-2.33 to -1.16)	⨁⨁◯◯ h<br>Low
MINO HCl	-	-	-1.77 (-2.44 to -1.10)	⨁⨁◯◯ h<br>Low	-1.77 (-2.44 to -1.10)	⨁⨁◯◯ h<br>Low
DOX	-	-	-1.90 (-2.71 to -1.09)	⨁⨁◯◯ h<br>Low	-1.90 (-2.71 to -1.09)	⨁⨁◯◯ h<br>Low
MET	-	-	-1.87 (-2.52 to -1.21)	⨁◯◯◯ i<br>Very low	-1.87 (-2.52 to -1.21)	⨁◯◯◯ i<br>Very low
SD alone	-1.87 (-2.43 to -1.30)	⨁⨁◯◯ b<br>Low	-	-	-1.87 (-2.43 to -1.30)	⨁⨁◯◯ h<br>Low
**MOX vs.**						
SZ	-	-	1.22 (0.59 to 1.86)	⨁⨁◯◯ h<br>Low	1.22 (0.59 to 1.86)	⨁⨁◯◯ h<br>Low
AMOX+MET	-0.09 (-0.56 to 0.38)	⨁⨁⨁◯ e<br>Moderate	-0.55 (-0.97 to -0.14)	⨁◯◯◯ i<br>Very low	-0.35 (-0.66 to -0.04)	⨁◯◯◯ i<br>Very low
CLM	-	-	-0.42 (-0.85 to 0.01)	⨁⨁⨁◯ g<br>Moderate	-0.42 (-0.85 to 0.01)	⨁⨁◯◯ e, g<br>Low
SEC	-	-	-0.44 (-1.00 to 0.12)	⨁⨁◯◯ g<br>Low	-0.44 (-1.00 to 0.12)	⨁◯◯◯ e, h<br>Very low

Comparisons	GRADE					
	Direct evidence		Indirect evidence		Network meta-analysis	
	MD (95% CI)	Certainty of evidence	MD (95% CI)	Certainty of evidence	MD (95% CI)	Certainty of evidence
CEF	-	-	-0.45 (-0.94 to 0.03)	⊕○○○ [i] Very low	-0.45 (-0.94 to 0.03)	⊕○○○ [e, i] Very low
AZ	-	-	-0.52 (-0.85 to -0.19)	⊕○○○ [i] Very low	-0.52 (-0.85 to -0.19)	⊕○○○ [i] Very low
MINO HCl	-	-	-0.55 (-1.01 to -0.08)	⊕⊕⊕○ [g] Moderate	-0.55 (-1.01 to -0.08)	⊕⊕⊕○ [g] Moderate
DOX	-	-	-0.67 (-1.32 to -0.02)	⊕⊕○○ [h] Low	-0.67 (-1.32 to -0.02)	⊕⊕○○ [h] Low
MET	-	-	-0.64 (-1.08 to -0.20)	⊕○○○ [i] Very low	-0.64 (-1.08 to -0.20)	⊕○○○ [i] Very low
SD alone	-0.75 (-1.06 to -0.44)	⊕⊕⊕⊕ High	0.21 (-0.67 to 1.08)	⊕⊕⊕○ [e, j] Moderate	-0.64 (-0.94 to -0.35)	⊕⊕○○ [g, k] Low
**AMOX+MET vs.**						
SZ	-	-	1.57 (0.99 to 2.15)	⊕○○○ [i] Very low	1.57 (0.99 to 2.15)	⊕○○○ [i] Very low
MOX	0.09 (-0.38 to 0.56)	⊕⊕⊕○ [e] Moderate	0.55 (0.14 to 0.97)	⊕○○○ [i] Very low	0.35 (0.44 to 0.66)	⊕○○○ [i] Very low
CLM	-	-	-0.07 (-0.41 to 0.26)	⊕○○○ [i] Very low	-0.07 (-0.41 to 0.26)	⊕○○○ [e, i] Very low
SEC	-	-	-0.09 (-0.58 to 0.40)	⊕○○○ [i] Very low	-0.09 (-0.58 to 0.40)	⊕○○○ [e, i] Very low
CEF	-0.04 (-0.48 to 0.40)	⊕○○○ [b, e] Very low	-0.33 (-1.15 to 0.48)	⊕⊕○○ [h] Low	-0.11 (-0.49 to 0.28)	⊕○○○ [e, h] Very low
AZ	-0.05 (-0.45 to 0.34)	⊕○○○ [a, c, e] Very low	-0.21 (-0.44 to 0.22)	⊕○○○ [i] Very low	-0.17 (-0.37 to 0.03)	⊕○○○ [e, i] Very low
MINO HCl	-	-	-0.20 (-0.59 to 0.19)	⊕○○○ [i] Very low	-0.20 (-0.59 to 0.19)	⊕○○○ [e, i] Very low
DOX	-0.06 (-0.72 to 0.60)	⊕⊕○○ [a, e] Low	-1.28 (-2.54 to -0.02)	⊕○○○ [i] Very low	-0.33 (-0.91 to 0.26)	⊕○○○ [e, i] Very low
MET	-0.06 (-0.44 to 0.32)	⊕○○○ [a, c, d, e] Very low	-1.09 (-1.80 to -0.39)	⊕○○○ [i] Very low	-0.29 (-0.63 to 0.04)	⊕○○○ [e, i, k] Very low
SD alone	-0.29 (-0.43 to -0.15)	⊕○○○ [a, c, d, e] Very low	-0.35 (-1.05 to 0.35)	⊕○○○ [i] Very low	-0.29 (-0.43 to -0.15)	⊕○○○ [i] Very low

Comparisons	GRADE					
	Direct evidence		Indirect evidence		Network meta-analysis	
	MD (95% CI)	Certainty of evidence	MD (95% CI)	Certainty of evidence	MD (95% CI)	Certainty of evidence
**CLM vs.**						
SZ	-	-	1.65 (1.00 to 2.29)	⊕⊕◯◯ [h] Low	1.65 (1.00 to 2.29)	⊕⊕◯◯ [h] Low
MOX	-	-	0.42 (0.01 to 0.85)	⊕⊕◯◯ [h] Low	0.42 (0.01 to 0.85)	⊕⊕◯◯ [h] Low
AMOX+MET	-	-	0.07 (0.26 to 0.41)	⊕◯◯◯ [i] Very low	0.07 (0.26 to 0.41)	⊕◯◯◯ [i] Very low
SEC	-	-	-0.02 (-0.58 to 0.54)	⊕⊕◯◯ [h] Low	-0.02 (-0.58 to 0.54)	⊕◯◯◯ [e, i] Very low
CEF	-	-	-0.03 (-0.53 to 0.46)	⊕⊕◯◯ [h] Low	-0.03 (-0.53 to 0.46)	⊕◯◯◯ [e, h] Very low
AZ	-	-	-0.10 (-0.44 to 0.25)	⊕◯◯◯ [i] Very low	-0.10 (-0.44 to 0.25)	⊕◯◯◯ [e, i] Very low
MINO HCl	-	-	-0.12 (-0.60 to 0.35)	⊕⊕◯◯ [h] Low	-0.12 (-0.60 to 0.35)	⊕◯◯◯ [e, i] Very low
DOX	-	-	-0.25 (-0.91 to 0.41)	⊕⊕◯◯ [h] Low	-0.25 (-0.91 to 0.41)	⊕◯◯◯ [e, i] Very low
MET	-	-	-0.22 (-0.67 to 0.23)	⊕◯◯◯ [i] Very low	-0.22 (-0.67 to 0.23)	⊕◯◯◯ [e, i] Very low
SD alone	-0.22 (-0.53 to 0.09)	⊕⊕◯◯ [d, e] Low	-	-	-0.22 (-0.53 to 0.09)	⊕◯◯◯ [e, i] Very low
**SEC vs.**						
SZ	-	-	1.67 (0.93 to 2.40)	⊕⊕◯◯ [h] Low	1.67 (0.93 to 2.40)	⊕⊕◯◯ [h] Low
MOX	-	-	0.44 (-0.12 to 1.00)	⊕⊕⊕◯ [g] Moderate	0.44 (-0.12 to 1.00)	⊕⊕◯◯ [e, g] Low
AMOX+MET	-	-	0.09 (-0.40 to 0.58)	⊕◯◯◯ [i] Very low	0.09 (-0.40 to 0.58)	⊕◯◯◯ [e, i] Very low
CLM	-	-	0.02 (-0.54 to 0.58)	⊕⊕◯◯ [h] Low	0.02 (-0.54 to 0.58)	⊕◯◯◯ [e, h] Very low
CEF	-	-	-0.01 (-0.62 to 0.60)	⊕⊕◯◯ [h] Low	-0.01 (-0.62 to 0.60)	⊕◯◯◯ [e, h] Very low
AZ	-	-	-0.08 (-0.57 to 0.42)	⊕◯◯◯ [i] Very low	-0.08 (-0.57 to 0.42)	⊕◯◯◯ [e, i] Very low

Comparisons	GRADE					
	Direct evidence		Indirect evidence		Network meta-analysis	
	MD (95% CI)	Certainty of evidence	MD (95% CI)	Certainty of evidence	MD (95% CI)	Certainty of evidence
MINO HCl	-	-	-0.11 (-0.70 to 0.49)	⊕⊕⊕◯ [g] Moderate	-0.11 (-0.70 to 0.49)	⊕⊕◯◯ [e, g] Low
DOX	-	-	-0.23 (-0.98 to 0.52)	⊕⊕◯◯ [h] Low	-0.23 (-0.98 to 0.52)	⊕◯◯◯ [e, h] Very low
MET	-	-	-0.20 (-0.78 to 0.37)	⊕◯◯◯ [i] Very low	-0.20 (-0.78 to 0.37)	⊕◯◯◯ [e, i] Very low
SD alone	-0.20 (-0.67 to 0.27)	⊕⊕⊕◯ [e] Moderate	-	-	-0.20 (-0.67 to 0.27)	⊕⊕◯◯ [e, g] Low
**CEF vs.**						
SZ	-	-	1.68 (0.99 to 2.36)	⊕⊕◯◯ [h] Low	1.68 (0.99 to 2.36)	⊕⊕◯◯ [h] Low
MOX	-	-	0.45 (-0.03 to 0.94)	⊕⊕◯◯ [h] Low	0.45 (-0.03 to 0.94)	⊕⊕◯◯ [h] Low
AMOX+MET	0.04 (-0.40 to 0.48)	⊕◯◯◯ [b, e] Very low	0.33 (-0.48 to 1.15)	⊕◯◯◯ [i] Very low	0.11 (-0.28 to 0.49)	⊕◯◯◯ [e, i] Very low
CLM	-	-	0.10 (-0.25 to 0.44)	⊕⊕◯◯ [h] Low	0.03 (-0.46 to 0.53)	⊕◯◯◯ [e, h] Very low
SEC	-	-	0.08 (-0.42 to 0.57)	⊕⊕◯◯ [h] Low	0.01 (-0.60 to 0.62)	⊕◯◯◯ [e, h] Very low
AZ	-	-	-0.06 (-0.48 to 0.35)	⊕◯◯◯ [i] Very low	-0.06 (-0.48 to 0.35)	⊕◯◯◯ [e, i] Very low
MINO HCl	-	-	-0.09 (-0.62 to 0.44)	⊕⊕◯◯ [h] Low	-0.09 (-0.62 to 0.44)	⊕◯◯◯ [e, h] Very low
DOX	-	-	-0.22 (-0.91 to 0.48)	⊕⊕◯◯ [h] Low	-0.22 (-0.91 to 0.48)	⊕◯◯◯ [e, h] Very low
MET	-	-	-0.19 (-0.69 to 0.31)	⊕◯◯◯ [i] Very low	-0.19 (-0.69 to 0.31)	⊕◯◯◯ [e, i] Very low
SD alone	-0.12 (-0.56 to 0.32)	⊕⊕◯◯ [b, e] Low	-0.42 (-1.24 to 0.40)	⊕⊕◯◯ [h] Low	-0.19 (-0.57 to 0.20)	⊕◯◯◯ [e, h] Very low
**AZ vs.**						
SZ	-	-	1.74 (1.16 to 2.33)	⊕◯◯◯ [i] Very low	1.74 (1.16 to 2.33)	⊕◯◯◯ [i] Very low

Comparisons	GRADE					
	Direct evidence		Indirect evidence		Network meta-analysis	
	MD (95% CI)	Certainty of evidence	MD (95% CI)	Certainty of evidence	MD (95% CI)	Certainty of evidence
MOX	-	-	0.52 (0.19 to 0.85)	⊕◯◯◯ [i]<br>Very low	0.52 (0.19 to 0.85)	⊕◯◯◯ [i]<br>Very low
AMOX+MET	0.05 (-0.34 to 0.45)	⊕◯◯◯ [a, c, e]<br>Very low	0.21 (-0.22 to 0.44)	⊕◯◯◯ [i]<br>Very low	0.17 (-0.03 to 0.37)	⊕◯◯◯ [e, i]<br>Very low
CLM		-	0.10 (-0.25 to 0.44)	⊕◯◯◯ [i]<br>Very low	0.10 (-0.25 to 0.44)	⊕◯◯◯ [e, i]<br>Very low
SEC	-	-	0.08 (-0.42 to 0.57)	⊕◯◯◯ [i]<br>Very low	0.08 (-0.42 to 0.57)	⊕◯◯◯ [e, i]<br>Very low
CEF	-	-	0.06 (-0.35 to 0.48)	⊕◯◯◯ [i]<br>Very low	0.06 (-0.35 to 0.48)	⊕◯◯◯ [e, i]<br>Very low
MINO HCl	-	-	-0.03 (-0.42 to 0.37)	⊕◯◯◯ [i]<br>Very low	-0.03 (-0.42 to 0.37)	⊕◯◯◯ [e, i]<br>Very low
DOX	-	-	-0.15 (-0.76 to 0.45)	⊕◯◯◯ [i]<br>Very low	-0.15 (-0.76 to 0.45)	⊕◯◯◯ [e, i]<br>Very low
MET	-	-	-0.12 (-0.49 to 0.24)	⊕◯◯◯ [i]<br>Very low	-0.12 (-0.49 to 0.24)	⊕◯◯◯ [e, i]<br>Very low
SD alone	-0.10 (-0.26 to 0.05)	⊕◯◯◯ [a, c, d, e]<br>Very low	-0.50 (-1.19 to 0.19)	⊕◯◯◯ [i]<br>Very low	-0.12 (-0.27 to 0.03)	⊕◯◯◯ [e, i]<br>Very low
**MINO HCl vs.**						
SZ	-	-	1.77 (1.10 to 2.44)	⊕⊕◯◯ [h]<br>Low	1.77 (1.10 to 2.44)	⊕⊕◯◯ [h]<br>Low
MOX	-	-	0.55 (0.08 to 1.01)	⊕⊕⊕◯ [g]<br>Moderate	0.55 (0.08 to 1.01)	⊕⊕⊕◯ [g]<br>Moderate
AMOX+MET	-	-	0.20 (-0.03 to 0.37)	⊕◯◯◯ [i]<br>Very low	0.20 (-0.03 to 0.37)	⊕◯◯◯ [e, i]<br>Very low
CLM	-	-	0.12 (-0.35 to 0.60)	⊕⊕◯◯ [h]<br>Low	0.12 (-0.35 to 0.60)	⊕◯◯◯ [e, h]<br>Very low
SEC	-	-	0.11 (-0.49 to 0.70)	⊕⊕⊕◯ [g]<br>Moderate	0.11 (-0.49 to 0.70)	⊕⊕◯◯ [e, h]<br>Low
CEF	-	-	0.09 (-0.44 to 0.62)	⊕⊕◯◯ [h]<br>Low	0.09 (-0.44 to 0.62)	⊕◯◯◯ [e, h]<br>Very low
AZ	-	-	0.03 (-0.37 to 0.42)	⊕◯◯◯ [i]<br>Very low	0.03 (-0.37 to 0.42)	⊕◯◯◯ [e, i]<br>Very low

Comparisons	GRADE					
	Direct evidence		Indirect evidence		Network meta-analysis	
	MD (95% CI)	Certainty of evidence	MD (95% CI)	Certainty of evidence	MD (95% CI)	Certainty of evidence
DOX	-	-	-0.13 (-0.81 to 0.56)	⊕◯◯◯ [i] Very low	-0.13 (-0.81 to 0.56)	⊕◯◯◯ [e, i] Very low
MET	-	-	-0.10 (-0.59 to 0.39)	⊕◯◯◯ [i] Very low	-0.10 (-0.59 to 0.39)	⊕◯◯◯ [e, i] Very low
SD alone	-0.09 (-0.46 to 0.27)	⊕⊕⊕◯ [e] Moderate	-	-	-0.09 (-0.46 to 0.27)	⊕⊕◯◯ [e, g] Low
**DOX vs.**						
SZ	-	-	1.90 (1.09 to 2.71)	⊕⊕◯◯ [h] Low	1.90 (1.09 to 2.71)	⊕⊕◯◯ [h] Low
MOX	-	-	0.67 (0.02 to 1.32)	⊕⊕◯◯ [h] Low	0.67 (0.02 to 1.32)	⊕⊕◯◯ [h] Low
AMOX+MET	0.06 (-0.60 to 0.72)	⊕⊕◯◯ [a, e] Low	1.28 (-0.02 to 2.53)	⊕◯◯◯ [i] Very low	0.33 (-0.26 to 0.91)	⊕◯◯◯ [e, i] Very low
CLM	-	-	0.25 (-0.41 to 0.91)	⊕⊕◯◯ [h] Low	0.25 (-0.41 to 0.91)	⊕◯◯◯ [e, i] Very low
SEC	-	-	0.23 (-0.52 to 0.98)	⊕⊕◯◯ [h] Low	0.23 (-0.52 to 0.98)	⊕◯◯◯ [e, i] Very low
CEF	-	-	0.22 (-0.48 to 0.91)	⊕⊕◯◯ [h] Low	0.22 (-0.48 to 0.91)	⊕◯◯◯ [e, I] Very low
AZ	-	-	0.15 (-0.45 to 0.76)	⊕◯◯◯ [i] Very low	0.15 (-0.45 to 0.76)	⊕◯◯◯ [e, i] Very low
MINO HCl	-	-	0.13 (-0.56 to 0.81)	⊕⊕◯◯ [h] Low	0.13 (-0.56 to 0.81)	⊕◯◯◯ [e, i] Very low
MET	0.14 (-0.53 to 0.81)	⊕⊕◯◯ [a, e] Low	-0.63 (-2.27 to 1.01)	⊕◯◯◯ [i] Very low	0.03 (-0.59 to 0.65)	⊕◯◯◯ [e, i] Very low
SD alone	0.15 (-0.48 to 0.78)	⊕⊕◯◯ [a, e] Low	-0.68 (-2.23 to 0.87)	⊕⊕◯◯ [h] Low	0.03 (-0.55 to 0.62)	⊕◯◯◯ [e, h] Very low
**MET vs.**						
SZ	-	-	1.87 (1.21 to 2.52)	⊕◯◯◯ [i] Very low	1.87 (1.21 to 2.52)	⊕◯◯◯ [i] Very low
MOX	-	-	0.64 (0.20 to 1.08)	⊕◯◯◯ [i] Very low	0.64 (0.20 to 1.08)	⊕◯◯◯ [i] Very low
AMOX+MET	0.06 (-0.32 to 0.44)	⊕◯◯◯ [a, c, d, e] Very low	1.09 (0.39 to 1.80)	⊕◯◯◯ [i] Very low	0.29 (0.04 to 0.63)	⊕◯◯◯ [e, i, k] Very low

Comparisons	GRADE					
	Direct evidence		Indirect evidence		Network meta-analysis	
	MD (95% CI)	Certainty of evidence	MD (95% CI)	Certainty of evidence	MD (95% CI)	Certainty of evidence
CLM	-	-	0.22 (-0.23 to 0.67)	⊕◯◯◯ [i] Very low	0.22 (-0.23 to 0.67)	⊕◯◯◯ [e, i] Very low
SEC	-	-	0.20 (-0.37 to 0.78)	⊕◯◯◯ [i] Very low	0.20 (-0.37 to 0.78)	⊕◯◯◯ [e, i] Very low
CEF	-	-	0.19 (-0.31 to 0.69)	⊕◯◯◯ [i] Very low	0.19 (-0.31 to 0.69)	⊕◯◯◯ [e, i] Very low
AZ	-	-	0.12 (-0.24 to 0.49)	⊕◯◯◯ [i] Very low	0.12 (-0.24 to 0.49)	⊕◯◯◯ [e, i] Very low
MINO HCl	-	-	0.10 (-0.39 to 0.59)	⊕◯◯◯ [i] Very low	0.10 (-0.39 to 0.59)	⊕◯◯◯ [e, i] Very low
DOX	-0.14 (-0.81 to 0.53)	⊕⊕◯◯ [a, e] Low	0.63 (-1.01 to 2.27)	⊕◯◯◯ [i] Very low	-0.03 (-0.65 to 0.59)	⊕◯◯◯ [e, i] Very low
SD alone	0.19 (-0.17 to 0.55)	⊕◯◯◯ [a, d, e] Very low	-0.99 (-1.82 to -0.15)	⊕◯◯◯ [i] Very low	0.00 (-0.33 to 0.33)	⊕◯◯◯ [e, i, k] Very low
**SD alone vs.**						
SZ	1.87 (1.30 to 2.43)	⊕⊕◯◯ [b] Low	-	-	1.87 (1.30 to 2.43)	⊕⊕◯◯ [g] Low
MOX	0.75 (0.44 to 1.06)	⊕⊕⊕⊕ High	-0.21 (-1.08 to 0.67)	⊕⊕⊕⊕ [j] High	0.64 (0.35 to 0.94)	⊕⊕⊕⊕ [j] High
AMOX+MET	0.29 (0.15 to 0.43)	⊕◯◯◯ [a, c, d, e] Very low	0.35 (-0.35 to 1.05)	⊕◯◯◯ [i] Very low	0.29 (0.15 to 0.43)	⊕◯◯◯ [i] Very low
CLM	0.22 (0.09 to 0.53)	⊕⊕◯◯ [d, e] Low	-	-	0.22 (-0.09 to 0.53)	⊕◯◯◯ [e, h] Very low
SEC	0.20 (-0.27 to 0.67)	⊕⊕⊕◯ [e] Moderate	-	-	0.20 (-0.27 to 0.67)	⊕⊕◯◯ [e, g] Low
CEF	0.12 (-0.32 to 0.56)	⊕◯◯◯ [b, e] Very low	0.42 (-0.40 to 1.24)	⊕◯◯◯ [i] Very low	0.19 (-0.20 to 0.57)	⊕◯◯◯ [e, i] Very low
AZ	0.10 (-0.05 to 0.26)	⊕◯◯◯ [a, c, d, e] Very low	0.50 (-0.19 to 1.19)	⊕◯◯◯ [i] Very low	0.12 (-0.03 to 0.27)	⊕◯◯◯ [e, i] Very low
MINO HCl	0.09 (-0.27 to 0.46)	⊕⊕⊕◯ [e] Moderate	-	-	0.09 (-0.27 to 0.46)	⊕⊕◯◯ [e, g] Low
DOX	-0.15 (-0.78 to 0.48)	⊕⊕◯◯ [a, e] Low	0.68 (-0.87 to 2.23)	⊕⊕◯◯ [h] Low	-0.03 (-0.62 to 0.55)	⊕◯◯◯ [e, i] Very low

Comparisons	GRADE					
	Direct evidence		Indirect evidence		Network meta-analysis	
	MD (95% CI)	Certainty of evidence	MD (95% CI)	Certainty of evidence	MD (95% CI)	Certainty of evidence
MET	-0.19 (-0.55 to 0.17)	⨁◯◯◯ a, d, e Very low	0.99 (0.15 to 1.82)	⨁◯◯◯ i Very low	0.00 (-0.33 to 0.33)	⨁◯◯◯ e, i, k Very low

[a] serious risk of bias; [b] very serious risk of bias; [c] serious inconsistency; [d] serious indirectness [e] serious imprecision; [f] publication bias; [g] contributing evidence of moderate quality; [h] contributing evidence of low quality; [i] contributing evidence of very low quality; [j] contributing evidence of high quality; [k] incoherence

Table S25. Certainty of evidence for intermediate-term CAL gain following the use of systemic antibiotics as adjunct to subgingival debridement

Comparisons	GRADE					
	Direct evidence		Indirect evidence		Network meta-analysis	
	MD (95% CI)	Certainty of evidence	MD (95% CI)	Certainty of evidence	MD (95% CI)	Certainty of evidence
**SZ vs.**						
MET	-	-	-1.10 (-2.00 to -0.20)	⨁⨁◯◯ h Low	-1.10 (-2.00 to -0.20)	⨁⨁◯◯ h Low
Clindamycin	-	-	-1.15 (-2.13 to -0.18)	⨁⨁◯◯ h Low	-1.15 (-2.13 to -0.18)	⨁⨁◯◯ h Low
MOX	-	-	-1.35 (-2.23 to -0.48)	⨁⨁◯◯ h Low	-1.35 (-2.23 to -0.48)	⨁⨁◯◯ h Low
AMOX+MET	-	-	-1.61 (-2.44 to -0.78)	⨁◯◯◯ i Very low	-1.61 (-2.44 to -0.78)	⨁◯◯◯ i Very low
DOX	-	-	-1.65 (-2.57 to -0.73)	⨁◯◯◯ i Very low	-1.65 (-2.57 to -0.73)	⨁◯◯◯ i Very low
AZ	-	-	-1.89 (-2.72 to -1.05)	⨁◯◯◯ i Very low	-1.89 (-2.72 to -1.05)	⨁◯◯◯ i Very low
CLM	-	-	-1.96 (-2.82 to -1.10)	⨁◯◯◯ i Very low	-1.96 (-2.82 to -1.10)	⨁◯◯◯ i Very low
MINO HCl	-	-	-2.00 (-2.98 to -1.02)	⨁⨁◯◯ h Low	-2.00 (-2.98 to -1.02)	⨁⨁◯◯ h Low
SD alone	-2.07 (-2.88 to -1.26)	⨁⨁◯◯ b Low	-	-	-2.07 (-2.88 to -1.26)	⨁⨁◯◯ h Low
**MET vs.**						
SZ	-	-	1.10 (0.20 to 2.00)	⨁⨁◯◯ h Low	1.10 (0.20 to 2.00)	⨁⨁◯◯ h Low
Clindamycin	-0.20 (-0.77 to 0.37)	⨁⨁⨁◯ e Moderate	1.04 (-0.49 to 2.58)	⨁⨁◯◯ h Low	-0.05 (-0.58 to 0.49)	⨁◯◯◯ e, h Very low
MOX	-	-	-0.25 (-0.77 to 0.27)	⨁⨁◯◯ h Low	-0.25 (-0.77 to 0.27)	⨁◯◯◯ e, h Very low

Comparisons	GRADE					
	Direct evidence		Indirect evidence		Network meta-analysis	
	MD (95% CI)	Certainty of evidence	MD (95% CI)	Certainty of evidence	MD (95% CI)	Certainty of evidence
AMOX+MET	-0.21 (-0.87 to 0.45)	⊕⊕◯◯ [a, e] Low	-0.71 (-1.25 to -0.17)	⊕◯◯◯ [i] Very low	-0.51 (-0.93 to -0.09)	⊕◯◯◯ [i] Very low
DOX	-0.56 (-1.01 to -0.11)	⊕◯◯◯ [a, c, d, e] Very low	-0.54 (-0.99 to -0.10)	⊕◯◯◯ [i] Very low	-0.54 (-0.99 to -0.10)	⊕◯◯◯ [i] Very low
AZ	-	-	-0.78 (-1.23 to -0.34)	⊕◯◯◯ [i] Very low	-0.78 (-1.23 to -0.34)	⊕◯◯◯ [i] Very low
CLM	-	-	-0.86 (-1.35 to -0.36)	⊕◯◯◯ [i] Very low	-0.86 (-1.35 to -0.36)	⊕◯◯◯ [i] Very low
MINO HCl	-	-	-0.90 (-1.58 to -0.22)	⊕⊕◯◯ [h] Low	-0.90 (-1.58 to -0.22)	⊕⊕◯◯ [h] Low
SD alone	-0.99 (-1.40 to -0.58)	⊕⊕◯◯ [a, c] Low	-0.71 (-2.04 to 0.62)	⊕⊕◯◯ [h] Low	-0.97 (-1.36 to -0.57)	⊕⊕◯◯ [h] Low
**Clindamycin vs.**						
SZ	-	-	1.15 (0.18 to 2.13)	⊕⊕◯◯ [h] Low	1.15 (0.18 to 2.13)	⊕⊕◯◯ [h] Low
MET	0.20 (-0.37 to 0.77)	⊕⊕⊕◯ [e] Moderate	1.04 (-0.49 to 2.58)	⊕⊕◯◯ [h] Low	0.05 (-0.49 to 0.58)	⊕◯◯◯ [e, i] Very low
MOX	-	-	-0.20 (-0.84 to 0.44)	⊕⊕⊕◯ [g] Moderate	-0.20 (-0.84 to 0.44)	⊕⊕◯◯ [e, g] Low
AMOX+MET	-	-	-0.46 (-1.03 to 0.10)	⊕◯◯◯ [i] Very low	-0.46 (-1.03 to 0.10)	⊕◯◯◯ [e, i] Very low
DOX	-0.50 (-1.09 to 0.09)	⊕⊕⊕◯ [e] Moderate	-0.47 (-2.06 to 1.11)	⊕◯◯◯ [i] Very low	-0.50 (-1.05 to 0.06)	⊕◯◯◯ [e, i] Very low
AZ	-	-	-0.74 (-1.32 to -0.15)	⊕◯◯◯ [i] Very low	-0.74 (-1.32 to -0.15)	⊕◯◯◯ [i] Very low
CLM	-	-	-0.81 (-1.43 to -0.19)	⊕◯◯◯ [i] Very low	-0.81 (-1.43 to -0.19)	⊕◯◯◯ [e, i] Very low
MINO HCl	-	-	-0.85 (-1.62 to -0.07)	⊕⊕⊕◯ [g] Moderate	-0.85 (-1.62 to -0.07)	⊕⊕⊕◯ [g] Moderate
SD alone	-1.10 (-1.70 to -0.50)	⊕⊕⊕◯ [a] Moderate	-0.11 (-1.37 to 1.16)	⊕⊕⊕◯ [g] Moderate	-0.92 (-1.46 to -0.38)	⊕⊕⊕◯ [g] Moderate
**MOX vs.**						
SZ	-	-	1.35 (0.48 to 2.23)	⊕⊕◯◯ [h] Low	1.35 (0.48 to 2.23)	⊕⊕◯◯ [h] Low

Comparisons	GRADE					
	Direct evidence		Indirect evidence		Network meta-analysis	
	MD (95% CI)	Certainty of evidence	MD (95% CI)	Certainty of evidence	MD (95% CI)	Certainty of evidence
MET	-	-	0.25 (-0.27 to 0.77)	⨁⨁◯◯ [h] Low	0.25 (-0.27 to 0.77)	⨁◯◯◯ [e, h] Very low
Clindamycin	-	-	0.20 (-0.44 to 0.84)	⨁⨁⨁◯ [g] Moderate	0.20 (-0.44 to 0.84)	⨁⨁◯◯ [e, g] Low
AMOX+MET	- 0.11 (-0.64to 0.42)	⨁⨁⨁◯ [e] Moderate	-0.39 (-0.90 to 0.11)	⨁◯◯◯ [i] Very low	-0.26 (-0.62 to 0.10)	⨁◯◯◯ [e, i] Very low
DOX	-	-	-0.29 (-0.84 to 0.25)	⨁◯◯◯ [i] Very low	-0.29 (-0.84 to 0.25)	⨁◯◯◯ [e, i] Very low
AZ	-	-	-0.53 (-0.93 to -0.13)	⨁◯◯◯ [i] Very low	-0.53 (-0.93 to -0.13)	⨁◯◯◯ [i] Very low
CLM	-	-	-0.61 (-1.06 to -0.15)	⨁◯◯◯ [i] Very low	-0.61 (-1.06 to -0.15)	⨁◯◯◯ [i] Very low
MINO HCl	-	-	-0.65 (-1.30 to 0.00)	⨁⨁⨁◯ [g] Moderate	-0.65 (-1.30 to 0.00)	⨁⨁◯◯ [e, h] Low
SD alone	-0.81 (-1.17 to -0.45)	⨁⨁⨁◯ [c] Moderate	0.01 (-0.99 to 1.02)	⨁⨁⨁◯ [g] Moderate	-0.72 (-1.06 to -0.38)	⨁⨁⨁◯ [g] Moderate
**AMOX+MET vs.**						
SZ	-	-	1.61 (0.78 to 2.44)	⨁◯◯◯ [i] Very low	1.61 (0.78 to 2.44)	⨁◯◯◯ [i] Very low
MET	0.21 (-0.45 to 0.87)	⨁⨁◯◯ [a, e] Low	0.71 (0.17 to 1.25)	⨁◯◯◯ [i] Very low	0.51 (0.09 to 0.93)	⨁◯◯◯ [i] Very low
Clindamycin	-	-	0.46 (-0.10 to 1.03)	⨁◯◯◯ [i] Very low	0.46 (-0.10 to 1.03)	⨁◯◯◯ [e, i] Very low
MOX	0.11 (-0.42 to 0.64)	⨁⨁⨁◯ [e] Moderate	0.39 (-0.11 to 0.90)	⨁◯◯◯ [i] Very low	0.26 (-0.10 to 0.62)	⨁◯◯◯ [e, i] Very low
DOX	-0.11 (-0.87 to 0.65)	⨁⨁◯◯ [a, e] Low	0.01 (-0.57 to 0.58)	⨁◯◯◯ [i] Very low	-0.04 (-0.49 to 0.42)	⨁◯◯◯ [e, i] Very low
AZ	-	-	-0.27 (-0.56 to 0.01)	⨁◯◯◯ [i] Very low	-0.27 (-0.56 to 0.01)	⨁◯◯◯ [e, i] Very low
CLM	-	-	-0.35 (-0.70 to 0.01)	⨁◯◯◯ [i] Very low	-0.35 (-0.70 to 0.01)	⨁◯◯◯ [e, i] Very low
MINO HCl	-	-	-0.39 (-0.97 to 0.20)	⨁◯◯◯ [i] Very low	-0.39 (-0.97 to 0.20)	⨁◯◯◯ [e, i] Very low

Comparisons	GRADE					
	Direct evidence		Indirect evidence		Network meta-analysis	
	MD (95% CI)	Certainty of evidence	MD (95% CI)	Certainty of evidence	MD (95% CI)	Certainty of evidence
SD alone	-0.45 (-0.64 to -0.26)	⊕○○○ [a, d, e]<br>Very low	-0.70 (-1.78 to 0.38)	⊕○○○ [i]<br>Very low	-0.46 (-0.64 to -0.27)	⊕○○○ [i]<br>Very low
**DOX vs.**						
SZ	-	-	1.65 (0.73 to 2.57)	⊕○○○ [i]<br>Very low	1.65 (0.73 to 2.57)	⊕○○○ [i]<br>Very low
MET	0.56 (-0.11 to 1.01)	⊕○○○ [a, c, d, e]<br>Very low	-5.32 (-13.60 to 2.96)	⊕○○○ [i]<br>Very low	0.54 (0.10 to 0.99)	⊕○○○ [i]<br>Very low
Clindamycin	0.50 (-0.09 to 1.09)	⊕⊕⊕○ [e]<br>Moderate	0.47 (-1.11 to 2.08)	⊕○○○ [i]<br>Very low	0.50 (-0.06 to 1.05)	⊕○○○ [e, i]<br>Very low
MOX	-	-	0.29 (-0.25 to 0.84)	⊕○○○ [i]<br>Very low	0.29 (-0.25 to 0.84)	⊕○○○ [e, i]<br>Very low
AMOX+MET	0.11 (-0.65 to 0.87)	⊕⊕○○ [a, e]<br>Low	-0.01 (-0.58 to 0.57)	⊕○○○ [i]<br>Very low	0.04 (-0.42 to 0.49)	⊕○○○ [e, h]<br>Very low
AZ	-	-	-0.24 (-0.72 to 0.24)	⊕○○○ [i]<br>Very low	-0.24 (-0.72 to 0.24)	⊕○○○ [e, i]<br>Very low
CLM	-	-	-0.31 (-0.84 to 0.22)	⊕○○○ [i]<br>Very low	-0.31 (-0.84 to 0.22)	⊕○○○ [e, i]<br>Very low
MINO HCl	-	-	-0.35 (-1.05 to 0.35)	⊕○○○ [i]<br>Very low	-0.35 (-1.05 to 0.35)	⊕○○○ [e, i]<br>Very low
SD alone	-0.49 (-0.94 to -0.04)	⊕○○○ [a, d, e]<br>Very low	0.37 (-1.18 to 1.92)	⊕○○○ [i]<br>Very low	-0.42 (-0.85 to 0.01)	⊕○○○ [e, i]<br>Very low
**AZ vs.**						
SZ	-	-	1.89 (1.05 to 2.72)	⊕○○○ [i]<br>Very low	1.89 (1.05 to 2.72)	⊕○○○ [i]<br>Very low
MET	-	-	0.78 (0.34 to 1.23)	⊕○○○ [i]<br>Very low	0.78 (0.34 to 1.23)	⊕○○○ [i]<br>Very low
Clindamycin	-	-	0.74 (0.15 to 1.32)	⊕○○○ [i]<br>Very low	0.74 (0.15 to 1.32)	⊕○○○ [i]<br>Very low
MOX	-	-	0.53 (0.13 to 0.93)	⊕○○○ [i]<br>Very low	0.53 (0.13 to 0.93)	⊕○○○ [i]<br>Very low
AMOX+MET	-	-	0.27 (-0.01 to 0.56)	⊕○○○ [i]<br>Very low	0.27 (-0.01 to 0.56)	⊕○○○ [e, i]<br>Very low
DOX	-	-	0.24 (-0.24 to 0.72)	⊕○○○ [i]<br>Very low	0.24 (-0.24 to 0.72)	⊕○○○ [e, i]<br>Very low

Comparisons	GRADE					
	Direct evidence		Indirect evidence		Network meta-analysis	
	MD (95% CI)	Certainty of evidence	MD (95% CI)	Certainty of evidence	MD (95% CI)	Certainty of evidence
CLM	-	-	-0.07 (-0.44 to 0.30)	⊕◯◯◯ [i]<br>Very low	-0.07 (-0.44 to 0.30)	⊕◯◯◯ [e, i]<br>Very low
MINO HCl	-	-	-0.11 (-0.70 to 0.48)	⊕◯◯◯ [i]<br>Very low	-0.11 (-0.70 to 0.48)	⊕◯◯◯ [e, i]<br>Very low
SD alone	-0.18 (-0.39 to 0.03)	⊕◯◯◯ [a, c, d, e]<br>Very low	-	-	-0.18 (-0.39 to 0.03)	⊕◯◯◯ [e, i]<br>Very low
**CLM vs.**						
SZ	-	-	1.96 (1.10 to 2.82)	⊕◯◯◯ [i]<br>Very low	1.96 (1.10 to 2.82)	⊕◯◯◯ [i]<br>Very low
MET	-	-	0.86 (0.36 to 1.35)	⊕◯◯◯ [i]<br>Very low	0.86 (0.36 to 1.35)	⊕◯◯◯ [i]<br>Very low
Clindamycin	-	-	0.81 (0.19 to 1.43)	⊕◯◯◯ [i]<br>Very low	0.81 (0.19 to 1.43)	⊕◯◯◯ [i]<br>Very low
MOX	-	-	0.61 (0.15 to 1.06)	⊕◯◯◯ [i]<br>Very low	0.61 (0.15 to 1.06)	⊕◯◯◯ [i]<br>Very low
AMOX+MET	-	-	0.35 (-0.01 to 0.70)	⊕◯◯◯ [i]<br>Very low	0.35 (-0.01 to 0.70)	⊕◯◯◯ [e, i]<br>Very low
DOX	-	-	0.31 (-0.22 to 0.84)	⊕◯◯◯ [i]<br>Very low	0.31 (-0.22 to 0.84)	⊕◯◯◯ [e, i]<br>Very low
AZ	-	-	0.07 (-0.30 to 0.44)	⊕◯◯◯ [i]<br>Very low	0.07 (-0.30 to 0.44)	⊕◯◯◯ [e, i]<br>Very low
MINO HCl	-	-	-0.04 (-0.67 to 0.59)	⊕◯◯◯ [i]<br>Very low	-0.04 (-0.67 to 0.59)	⊕◯◯◯ [e, i]<br>Very low
SD alone	-0.11 (-0.41 to 0.19)	⊕◯◯◯ [c, d, e]<br>Very low	-	-	-0.11 (-0.41 to 0.19)	⊕◯◯◯ [e, i]<br>Very low
**MINO HCl vs.**						
SZ	-	-	2.00 (1.02 to 2.98)	⊕⊕◯◯ [h]<br>Low	2.00 (1.02 to 2.98)	⊕⊕◯◯ [h]<br>Low
MET	-	-	0.90 (0.22 to 1.58)	⊕⊕◯◯ [h]<br>Low	0.90 (0.22 to 1.58)	⊕⊕◯◯ [h]<br>Low
Clindamycin	-	-	0.85 (0.07 to 1.62)	⊕⊕⊕◯ [g]<br>Moderate	0.85 (0.07 to 1.62)	⊕⊕⊕◯ [g]<br>Moderate
MOX	-	-	0.65 (0.00 to 1.30)	⊕⊕⊕◯ [g]<br>Moderate	0.65 (0.00 to 1.30)	⊕⊕◯◯ [e, g]<br>Low

Comparisons	GRADE					
	Direct evidence		Indirect evidence		Network meta-analysis	
	MD (95% CI)	Certainty of evidence	MD (95% CI)	Certainty of evidence	MD (95% CI)	Certainty of evidence
AMOX+MET	-	-	0.39 (-0.20 to 0.97)	⨁◯◯◯ [i] Very low	0.39 (-0.20 to 0.97)	⨁◯◯◯ [e, i] Very low
DOX	-	-	0.35 (-0.35 to 1.05)	⨁◯◯◯ [i] Very low	0.35 (-0.35 to 1.05)	⨁◯◯◯ [e, i] Very low
AZ	-	-	0.11 (-0.48 to 0.70)	⨁◯◯◯ [i] Very low	0.11 (-0.48 to 0.70)	⨁◯◯◯ [e, i] Very low
CLM	-	-	0.04 (-0.59 to 0.67)	⨁◯◯◯ [i] Very low	0.04 (-0.59 to 0.67)	⨁◯◯◯ [e, i] Very low
SD alone	-0.07 (-0.62 to 0.48)	⨁⨁⨁◯ [e] Moderate	-	-	-0.07 (-0.62 to 0.48)	⨁⨁◯◯ [e, g] Low
**SD alone vs.**						
SZ	2.07 (1.26 to 2.88)	⨁⨁◯◯ [b] Low	-	-	2.07 (1.26 to 2.88)	⨁⨁◯◯ [h] Low
MET	0.99 (0.58 to 1.40)	⨁⨁◯◯ [a, c] Low	0.71 (-0.62 to 2.04)	⨁⨁◯◯ [h] Low	0.97 (0.57 to 1.36)	⨁⨁◯◯ [h] Low
Clindamycin	1.10 (0.50 to 1.70)	⨁⨁⨁◯ [a] Moderate	0.11 (-1.16 to 1.37)	⨁⨁⨁◯ [g] Moderate	0.92 (0.38 to 1.46)	⨁⨁⨁◯ [g] Moderate
MOX	0.81 (0.45 to 1.17)	⨁⨁⨁◯ [c] Moderate	-0.01 (-1.02 to 0.99)	⨁⨁⨁◯ [g] Moderate	0.72 (0.38 to 1.06)	⨁⨁⨁◯ [g] Moderate
AMOX+MET	0.45 (0.26 to 0.64)	⨁◯◯◯ [a, d, e] Very low	0.70 (-0.38 to 1.78)	⨁◯◯◯ [i] Very low	0.46 (0.27 to 0.64)	⨁◯◯◯ [i] Very low
DOX	0.49 (0.04 to 0.94)	⨁◯◯◯ [a, d, e] Very low	-0.37 (-1.92 to 1.18)	⨁◯◯◯ [i] Very low	0.42 (-0.01 to 0.85)	⨁◯◯◯ [e, i] Very low
AZ	0.18 (0.03 to -0.39)	⨁◯◯◯ [a, c, d, e] Very low	-	-	0.18 (-0.03 to 0.39)	⨁◯◯◯ [e, i] Very low
CLM	0.11 (0.19 to -0.41)	⨁◯◯◯ [c, d, e] Very low	-	-	0.11 (-0.19 to 0.41)	⨁◯◯◯ [e, i] Very low
MINO HCl	0.07 (-0.48 to 0.62)	⨁⨁⨁◯ [e] Moderate	-	-	0.07 (-0.48 to 0.62)	⨁⨁◯◯ [e, g] Low

[a] serious risk of bias; [b] very serious risk of bias; [c] serious inconsistency; [d] serious indirectness [e] serious imprecision; [f] publication bias; [g] contributing evidence of moderate quality; [h] contributing evidence of low quality; [i] contributing evidence of very low quality; [j] contributing evidence of high quality; [k] incoherence

Table S26. Certainty of evidence for long-term CAL gain following the use of systemic antibiotics as adjunct to subgingival debridement

Comparisons	GRADE					
	Direct evidence		Indirect evidence		Network meta-analysis	
	MD (95% CI)	Certainty of evidence	MD (95% CI)	Certainty of evidence	MD (95% CI)	Certainty of evidence
**MET vs.**						
Clindamycin	-0.30 (-0.69 to 0.09)	⊕⊕⊕◯ [a] Moderate	-	-	-0.30 (-0.69 to 0.09)	⊕⊕◯◯ [e, g] Low
DOX	-1.30 (-1.69 to-0.91)	⊕⊕⊕◯ [a] Moderate	-	-	-1.30 (-1.69 to -0.91)	⊕⊕⊕◯ [g] Moderate
AMOX+MET	-	-	-1.37 (-1.77 to -0.97)	⊕⊕◯◯ [h] Low	-1.37 (-1.77 to -0.97)	⊕⊕◯◯ [h] Low
SD alone	-1.80 (-2.18 to-1.42)	⊕⊕⊕◯ [a] Moderate	-	-	-1.80 (-2.18 to -1.42)	⊕⊕⊕◯ [g] Moderate
AZ	-	-	-1.85 (-2.29 to -1.40)	⊕◯◯◯ [i] Very low	-1.85 (-2.29 to -1.40)	⊕◯◯◯ [i] Very low
**Clindamycin vs.**						
MET	0.30 (-0.09 to 0.69)	⊕⊕⊕◯ [a] Moderate	-	-	0.30 (-0.09 to 0.69)	⊕⊕◯◯ [e, g] Low
DOX	-1.00 (-1.41to-0.59)	⊕⊕⊕◯ [a] Moderate	-	-	-1.00 (-1.41 to -0.59)	⊕⊕⊕◯ [g] Moderate
AMOX+MET	-	-	-1.07 (-1.49 to-0.65)	⊕⊕◯◯ [h] Low	-1.07 (-1.49 to -0.65)	⊕⊕◯◯ [h] Low
SD alone	-1.50 (-1.90to-1.10)	⊕⊕⊕◯ [a] Moderate	-	-	-1.50 (-1.90 to -1.10)	⊕⊕⊕◯ [g] Moderate
AZ	-	-	-1.55 (-2.01to -1.08)	⊕◯◯◯ [i] Very low	-1.55 (-2.01 to-1.08)	⊕◯◯◯ [i] Very low
**DOX vs.**						
MET	1.30 (0.91 to 1.69)	⊕⊕⊕◯ [a] Moderate	-	-	1.30 (0.91 to 1.69)	⊕⊕⊕◯ [g] Moderate
Clindamycin	1.00 (0.59 to 1.41)	⊕⊕⊕◯ [a] Moderate	-	-	1.00 (0.59 to 1.41)	⊕⊕⊕◯ [g] Moderate
AMOX+MET	-	-	-0.07 (-0.49 to 0.35)	⊕⊕◯◯ [h] Low	-0.07 (-0.49 to 0.35)	⊕◯◯◯ [e, h] Very low
SD alone	-0.50 (-0.90 to-0.10)	⊕⊕⊕◯ [a] Moderate	-	-	-0.50 (-0.90 to -0.10)	⊕⊕⊕◯ [g] Moderate
AZ	-	-	-0.55 (-1.01 to-0.09)	⊕◯◯◯ [i] Very low	-0.55 (-1.01 to -0.09)	⊕◯◯◯ [i] Very low
**AMOX+MET vs.**						

Comparisons	GRADE					
	Direct evidence		Indirect evidence		Network meta-analysis	
	MD (95% CI)	Certainty of evidence	MD (95% CI)	Certainty of evidence	MD (95% CI)	Certainty of evidence
MET	-	-	1.37 (0.97 to 1.77)	⊕⊕○○ [h] Low	1.37 (0.97 to 1.77)	⊕⊕○○ [h] Low
Clindamycin	-	-	1.07 (0.65 to 1.49)	⊕⊕○○ [h] Low	1.07 (0.65 to 1.49)	⊕⊕○○ [h] Low
DOX	-	-	0.07 (-0.35 to 0.49)	⊕⊕○○ [h] Low	0.07 (-0.35 to 0.49)	⊕○○○ [e, h] Very low
SD alone	-0.43 (-0.56 to -0.29)	⊕⊕○○ [b] Low	-	-	-0.43 (-0.56 to -0.29)	⊕⊕○○ [h] Low
AZ	-	-	-0.47 (-0.74 to -0.20)	⊕○○○ [i] Very low	-0.47 (-0.74 to -0.20)	⊕○○○ [i] Very low
**SD alone vs.**						
MET	1.80 (1.42 to 2.18)	⊕⊕⊕○ [a] Moderate	-	-	1.80 (1.42 to 2.18)	⊕⊕⊕○ [g] Moderate
Clindamycin	1.50 (1.10 to 1.90)	⊕⊕⊕○ [a] Moderate	-	-	1.50 (1.10 to 1.90)	⊕⊕⊕○ [g] Moderate
DOX	0.50 (0.10 to 0.90)	⊕⊕⊕○ [a] Moderate	-	-	0.50 (0.10 to 0.90)	⊕⊕⊕○ [g] Moderate
AMOX+MET	0.43 (0.29 to 0.56)	⊕⊕○○ [b] Low	-	-	0.43 (0.29 to 0.56)	⊕⊕○○ [h] Low
AZ	-0.05 (-0.28to 0.19)	⊕⊕○○ [a, c, d, e] Very low	-	-	-0.05 (-0.28to 0.19)	⊕○○○ [e, i] Very low
**AZ vs.**						
MET	-	-	1.85 (1.08 to 2.29)	⊕○○○ [i] Very low	1.85 (1.08 to 2.29)	⊕○○○ [i] Very low
Clindamycin	-	-	1.55 (1.08 to 2.01)	⊕○○○ [i] Very low	1.55 (1.08 to 2.01)	⊕○○○ [i] Very low
DOX	-	-	0.55 (0.09 to 1.01)	⊕○○○ [i] Very low	0.55 (0.09 to 1.01)	⊕○○○ [i] Very low
AMOX+MET	-	-	0.47 (0.20 to 0.74)	⊕○○○ [i] Very low	0.47 (0.20 to 0.74)	⊕○○○ [i] Very low
SD alone	0.05 (-0.19 to 0.28)	⊕⊕○○ [a, c, d, e] Very low	-	-	0.05 (-0.19 to 0.28)	⊕○○○ [e, i] Very low

[a] serious risk of bias; [b] very serious risk of bias; [c] serious inconsistency; [d] serious indirectness [e] serious imprecision; [f] publication bias; [g] contributing evidence of moderate quality; [h] contributing evidence of low quality; [i] contributing evidence of very low quality; [j] contributing evidence of high quality; [k] incoherence

Table S27. Certainty of evidence for short-term PPD reduction following the use of systemic antibiotics as adjunct to subgingival debridement

Comparisons	GRADE					
	Direct evidence		Indirect evidence		Network meta-analysis	
	MD (95% CI)	Certainty of evidence	MD (95% CI)	Certainty of evidence	MD (95% CI)	Certainty of evidence
**SZ vs.**						
MET	-	-	-1.05 (-1.97 to -0.12)	⊕⊕◯◯ [h] Low	-1.05 (-1.97 to -0.12)	⊕⊕◯◯ [h] Low
MOX	-	-	-1.05 (-1.98 to -0.12)	⊕⊕◯◯ [h] Low	-1.05 (-1.98 to -0.12)	⊕⊕◯◯ [h] Low
AMOX+MET	-	-	-1.11 (-1.89 to -0.32)	⊕◯◯◯ [i] Very low	-1.11 (-1.89 to -0.32)	⊕◯◯◯ [i] Very low
CLM	-	-	-1.09 (-2.04 to -0.14)	⊕◯◯◯ [i] Very low	-1.09 (-2.04 to -0.14)	⊕◯◯◯ [i] Very low
CEF	-	-	-1.36 (-2.40 to -0.32)	⊕◯◯◯ [i] Very low	-1.36 (-2.40 to -0.32)	⊕◯◯◯ [i] Very low
SEC	-	-	-1.46 (-2.58 to -0.33)	⊕⊕◯◯ [h] Low	-1.46 (-2.58 to -0.33)	⊕⊕◯◯ [h] Low
MINO HCl	-	-	-1.47 (-2.45 to -0.49)	⊕⊕◯◯ [h] Low	-1.47 (-2.45 to -0.49)	⊕⊕◯◯ [h] Low
AZ	-	-	-1.49 (-2.28 to -0.69)	⊕◯◯◯ [i] Very low	-1.49 (-2.28 to -0.69)	⊕◯◯◯ [i] Very low
DOX	-	-	-1.66 (-2.71 to -0.61)	⊕⊕◯◯ [h] Low	-1.66 (-2.71 to -0.61)	⊕⊕◯◯ [h] Low
SD alone	-1.66 (-2.41 to -0.91)	⊕⊕◯◯ [b] Low	-	-	-1.66 (-2.41 to -0.91)	⊕⊕◯◯ [h] Low
**MET vs.**						
SZ	-	-	1.05 (0.12 to 1.97)	⊕⊕◯◯ [h] Low	1.05 (0.12 to 1.97)	⊕⊕◯◯ [h] Low
MOX	-		-0.01 (-0.76 to 0.75)	⊕⊕◯◯ [h] Low	-0.01 (-0.76 to 0.75)	⊕◯◯◯ [e, h] Very low
AMOX+MET	0.13 (-0.48 to 0.74)	⊕◯◯◯ [a, c, d, e] Very low	-0.75 (-1.90 to 0.40)	⊕◯◯◯ [i] Very low	-0.06 (-0.60 to 0.48)	⊕◯◯◯ [e, i] Very low
CLM	-	-	-0.05 (-0.84 to 0.75)	⊕◯◯◯ [i] Very low	-0.05 (-0.84 to 0.75)	⊕◯◯◯ [e, i] Very low
CEF	-	-	-0.31 (-1.19 to 0.57)	⊕◯◯◯ [i] Very low	-0.31 (-1.19 to 0.57)	⊕◯◯◯ [e, i] Very low

Comparisons	GRADE					
	Direct evidence		Indirect evidence		Network meta-analysis	
	MD (95% CI)	Certainty of evidence	MD (95% CI)	Certainty of evidence	MD (95% CI)	Certainty of evidence
SEC	-	-	-0.41 (-1.41 to 0.59)	⊕⊕◯◯ [h]<br>Low	-0.41 (-1.41 to 0.59)	⊕◯◯◯ [e, h]<br>Very low
MINO HCl	-	-	-0.43 (-1.25 to 0.40)	⊕⊕◯◯ [h]<br>Low	-0.43 (-1.25 to 0.40)	⊕◯◯◯ [e, h]<br>Very low
AZ	-	-	-0.44 (-1.03 to 0.15)	⊕◯◯◯ [i]<br>Very low	-0.44 (-1.03 to 0.15)	⊕◯◯◯ [e, i]<br>Very low
DOX	-	-	-1.34 (-3.31 to 0.63)	⊕⊕◯◯ [h]<br>Low	-0.61 (-1.39 to 0.16)	⊕◯◯◯ [e, h]<br>Very low
SD alone	-0.80 (-1.40 to -0.19)	⊕⊕◯◯ [a, e]<br>Low	0.12 (-1.07 to 1.31)	⊕⊕◯◯ [h]<br>Low	-0.61 (-1.15 to -0.07)	⊕◯◯◯ [e, h]<br>Very low
**MOX vs.**						
SZ	-	-	1.05 (0.12 to 1.98)	⊕⊕◯◯ [h]<br>Low	1.05 (0.12 to 1.98)	⊕⊕◯◯ [h]<br>Low
MET	-	-	0.01 (-0.75 to 0.76)	⊕⊕◯◯ [h]<br>Low	0.01 (-0.75 to 0.76)	⊕◯◯◯ [e, i]<br>Very low
AMOX+MET	0.09 (-0.74 to 0.92)	⊕⊕⊕◯ [e]<br>Moderate	-0.18 (-0.96 to 0.60)	⊕◯◯◯ [i]<br>Very low	-0.05 (-0.62 to 0.51)	⊕⊕◯◯ [e, g]<br>Low
CLM	-	-	-0.04 (-0.84 to 0.76)	⊕◯◯◯ [i]<br>Very low	-0.04 (-0.84 to 0.76)	⊕◯◯◯ [e, i]<br>Very low
CEF	-	-	-0.30 (-1.20 to 0.59)	⊕◯◯◯ [i]<br>Very low	-0.30 (-1.20 to 0.59)	⊕◯◯◯ [e, i]<br>Very low
SEC	-	-	-0.40 (-1.41 to 0.60)	⊕⊕◯◯ [h]<br>Low	-0.40 (-1.41 to 0.60)	⊕◯◯◯ [e, h]<br>Very low
MINO HCl	-	-	-0.42 (-1.25 to 0.41)	⊕⊕◯◯ [h]<br>Low	-0.42 (-1.25 to 0.41)	⊕◯◯◯ [e, h]<br>Very low
AZ	-	-	-0.43 (-1.04 to 0.17)	⊕◯◯◯ [i]<br>Very low	-0.43 (-1.04 to 0.17)	⊕◯◯◯ [e, i]<br>Very low
DOX	-	-	-0.61 (-1.51 to 0.30)	⊕⊕◯◯ [h]<br>Low	-0.61 (-1.51 to 0.30)	⊕◯◯◯ [e, i]<br>Very low
SD alone	-0.71 (-1.29 to -0.12)	⊕⊕◯◯ [a, d]<br>Low	0.10 (-1.43 to 1.62)	⊕⊕◯◯ [h]<br>Low	-0.60 (-1.15 to -0.06)	⊕◯◯◯ [i]<br>Very low
**AMOX+MET vs.**						
SZ	-	-	1.11 (0.32 to 1.89)	⊕◯◯◯ [i]<br>Very low	1.11 (0.32 to 1.89)	⊕◯◯◯ [i]<br>Very low

Comparisons	GRADE					
	Direct evidence		Indirect evidence		Network meta-analysis	
	MD (95% CI)	Certainty of evidence	MD (95% CI)	Certainty of evidence	MD (95% CI)	Certainty of evidence
MET	-0.13 (-0.74 to 0.48)	⊕○○○ [a, c, d, e] Very low	0.75 (-0.40 to 1.90)	⊕○○○ [i] Very low	0.06 (-0.48 to 0.60)	⊕○○○ [e, i] Very low
MOX	-0.09 (-0.92 to .74)	⊕⊕⊕○ [e] Moderate	0.18 (-0.60 to 0.96)	⊕○○○ [i] Very low	0.05 (-0.51 to 0.62)	⊕⊕○○ [e, g] Low
CLM	-	-	0.02 (-0.62 to 0.65)	⊕○○○ [i] Very low	0.02 (-0.62 to 0.65)	⊕○○○ [e, i] Very low
CEF	0.01 (-0.81 to 0.83)	⊕○○○ [b, e] Very low	-1.12 (-2.63 to 0.38)	⊕○○○ [i] Very low	-0.25 (-0.97 to 0.47)	⊕○○○ [e, i] Very low
SEC	-	-	-0.35 (-1.22 to 0.52)	⊕○○○ [i] Very low	-0.35 (-1.22 to 0.52)	⊕○○○ [e, i] Very low
MINO HCl	-	-	-0.36 (-1.03 to 0.31)	⊕○○○ [i] Very low	-0.36 (-1.03 to 0.31)	⊕○○○ [e, i] Very low
AZ	-0.19 (-0.80 to 0.42)	⊕○○○ [a, c, d, e] Very low	-0.46 (-0.88 to 0.06)	⊕○○○ [ei] Very low	-0.38 (-0.71 to -0.05)	⊕○○○ [i] Very low
DOX	-0.43 (-1.29 to 0.43)	⊕⊕⊕○ [a] Moderate	-0.86 (-2.24 to 0.51)	⊕⊕○○ [h] Low	-0.55 (-1.28 to 0.18)	⊕○○○ [e, h] Very low
SD alone	-0.56 (-0.81 to -0.32)	⊕○○○ [a, c, d, e, f] Very low	-0.27 (-1.33 to 0.79)	⊕○○○ [e, i] Very low	-0.55 (-0.79 to -0.31)	⊕○○○ [i] Very low
**CLM vs.**						
SZ	-	-	1.09 (0.14 to 2.04)	⊕○○○ [i] Very low	1.09 (0.14 to 2.04)	⊕○○○ [i] Very low
MET	-	-	0.05 (-0.75 to 0.84)	⊕○○○ [i] Very low	0.05 (-0.75 to 0.84)	⊕○○○ [e, i] Very low
MOX	-	-	0.04 (-0.76 to 0.84)	⊕○○○ [i] Very low	0.04 (-0.76 to 0.84)	⊕○○○ [e, i] Very low
AMOX+MET	-	-	-0.02 (-0.65 to 0.62)	⊕○○○ [i] Very low	-0.02 (-0.65 to 0.62)	⊕○○○ [e, i] Very low
CEF	-	-	-0.26 (-1.19 to 0.66)	⊕○○○ [i] Very low	-0.26 (-1.19 to 0.66)	⊕○○○ [e, i] Very low
SEC	-	-	-0.36 (-1.39 to 0.66)	⊕○○○ [i] Very low	-0.36 (-1.39 to 0.66)	⊕○○○ [e, i] Very low
MINO HCl	-	-	-0.38 (-1.24 to 0.48)	⊕○○○ [i] Very low	-0.38 (-1.24 to 0.48)	⊕○○○ [e, i] Very low

Comparisons	GRADE					
	Direct evidence		Indirect evidence		Network meta-analysis	
	MD (95% CI)	Certainty of evidence	MD (95% CI)	Certainty of evidence	MD (95% CI)	Certainty of evidence
AZ	-	-	-0.39 (-1.04 to 0.25)	⊕◯◯◯ [i]<br>Very low	-0.39 (-1.04 to 0.25)	⊕◯◯◯ [e, i]<br>Very low
DOX	-	-	-0.57 (-1.50 to 0.37)	⊕◯◯◯ [i]<br>Very low	-0.57 (-1.50 to 0.37)	⊕◯◯◯ [e, i]<br>Very low
SD alone	-0.56 (-1.15 to 0.02)	⊕◯◯◯ [a, c, d, e]<br>Very low	-	-	-0.56 (-1.15 to 0.02)	⊕◯◯◯ [e, i]<br>Very low
**CEF vs.**						
SZ	-	-	1.36 (0.32 to 2.40)	⊕◯◯◯ [i]<br>Very low	1.36 (0.32 to 2.40)	⊕◯◯◯ [i]<br>Very low
MET	-	-	0.31 (-0.57 to 1.19)	⊕◯◯◯ [i]<br>Very low	0.31 (-0.57 to 1.19)	⊕◯◯◯ [e, i]<br>Very low
MOX	-	-	0.30 (-0.59 to 1.20)	⊕◯◯◯ [i]<br>Very low	0.30 (-0.59 to 1.20)	⊕◯◯◯ [e, i]<br>Very low
AMOX+MET	-0.01 (-0.83 to 0.81)	⊕◯◯◯ [b, e]<br>Very low	1.12 (-0.38 to 2.63)	⊕◯◯◯ [i]<br>Very low	0.25 (-0.47 to 0.97)	⊕◯◯◯ [e, i]<br>Very low
CLM	-	-	0.26 (-0.66 to 1.19)	⊕◯◯◯ [i]<br>Very low	0.26 (-0.66 to 1.19)	⊕◯◯◯ [e, i]<br>Very low
SEC	-	-	-0.10 (-1.21 to 1.01)	⊕◯◯◯ [i]<br>Very low	-0.10 (-1.21 to 1.01)	⊕◯◯◯ [e, i]<br>Very low
MINO HCl	-	-	-0.11 (-1.07 to 0.84)	⊕◯◯◯ [i]<br>Very low	-0.11 (-1.07 to 0.84)	⊕◯◯◯ [e, i]<br>Very low
AZ	-	-	-0.13 (-0.89 to 0.63)	⊕◯◯◯ [i]<br>Very low	-0.13 (-0.89 to 0.63)	⊕◯◯◯ [e, i]<br>Very low
DOX	-	-	-0.30 (-1.31 to 0.71)	⊕⊕◯◯ [h]<br>Low	-0.30 (-1.31 to 0.71)	⊕◯◯◯ [e, h]<br>Very low
SD alone	-0.04 (-0.86 to 0.78)	⊕◯◯◯ [b, e]<br>Very low	-1.17 (-2.68 to 0.33)	⊕◯◯◯ [i]<br>Very low	-0.30 (-1.02 to 0.42)	⊕◯◯◯ [e, i]<br>Very low
**SEC vs.**						
SZ	-	-	1.46 (0.33 to 2.58)	⊕⊕◯◯ [h]<br>Low	1.46 (0.33 to 2.58)	⊕⊕◯◯ [h]<br>Low
MET	-	-	0.41 (-0.59 to 1.41)	⊕⊕◯◯ [h]<br>Low	0.41 (-0.59 to 1.41)	⊕◯◯◯ [e, h]<br>Very low
MOX	-	-	0.40 (-0.60 to 1.41)	⊕⊕◯◯ [h]<br>Low	0.40 (-0.60 to 1.41)	⊕◯◯◯ [e, h]<br>Very low

Comparisons	GRADE					
	Direct evidence		Indirect evidence		Network meta-analysis	
	MD (95% CI)	Certainty of evidence	MD (95% CI)	Certainty of evidence	MD (95% CI)	Certainty of evidence
AMOX+MET	-	-	0.35 (-0.52 to 1.22)	⊕○○○ [i] Very low	0.35 (-0.52 to 1.22)	⊕○○○ [e, i] Very low
CLM	-	-	0.36 (-0.66 to 1.39)	⊕○○○ [i] Very low	0.36 (-0.66 to 1.39)	⊕○○○ [e, i] Very low
CEF	-	-	0.10 (-1.01 to 1.21)	⊕○○○ [i] Very low	0.10 (-1.01 to 1.21)	⊕○○○ [e, i] Very low
MINO HCl	-	-	-0.02 (-1.06 to 1.03)	⊕⊕○○ [h] Low	-0.02 (-1.06 to 1.03)	⊕○○○ [e, h] Very low
AZ	-	-	-0.03 (-0.91 to 0.85)	⊕○○○ [i] Very low	-0.03 (-0.91 to 0.85)	⊕○○○ [e, i] Very low
DOX	-	-	-0.20 (-1.32 to 0.91)	⊕⊕○○ [h] Low	-0.20 (-1.32 to 0.91)	⊕○○○ [e, h] Very low
SD alone	-0.20 (-1.04 to 0.64)	⊕⊕○○ [a, e] Low	-	-	-0.20 (-1.04 to 0.64)	⊕○○○ [e, h] Very low
**MINO HCl vs.**						
SZ	-	-	1.47 (0.49 to 2.45)	⊕⊕○○ [h] Low	1.47 (0.49 to 2.45)	⊕⊕○○ [h] Low
MET	-	-	0.43 (-0.40 to 1.25)	⊕⊕○○ [h] Low	0.43 (-0.40 to 1.25)	⊕○○○ [e, h] Very low
MOX	-	-	0.42 (-0.41 to 1.25)	⊕⊕○○ [h] Low	0.42 (-0.41 to 1.25)	⊕○○○ [e, h] Very low
AMOX+MET	-	-	0.36 (-0.31 to 1.03)	⊕○○○ [i] Very low	0.36 (-0.31 to 1.03)	⊕○○○ [e, i] Very low
CLM	-	-	0.38 (-0.48 to 1.24)	⊕○○○ [i] Very low	0.38 (-0.48 to 1.24)	⊕○○○ [e, i] Very low
CEF	-	-	0.11 (-0.84 to 1.07)	⊕○○○ [i] Very low	0.11 (-0.84 to 1.07)	⊕○○○ [e, i] Very low
SEC	-	-	0.02 (-1.03 to 1.06)	⊕⊕○○ [h] Low	0.02 (-1.03 to 1.06)	⊕○○○ [e, h] Very low
AZ	-	-	-0.02 (-0.70 to 0.66)	⊕○○○ [i] Very low	-0.02 (-0.70 to 0.66)	⊕○○○ [e, i] Very low
DOX	-	-	-0.19 (-1.15 to 0.77)	⊕⊕○○ [h] Low	-0.19 (-1.15 to 0.77)	⊕○○○ [e, h] Very low

Comparisons	GRADE					
	Direct evidence		Indirect evidence		Network meta-analysis	
	MD (95% CI)	Certainty of evidence	MD (95% CI)	Certainty of evidence	MD (95% CI)	Certainty of evidence
SD alone	-0.18 (-0.81 to 0.44)	⊕⊕○○ [a, e] Low	-	-	-0.18 (-0.81 to 0.44)	⊕○○○ [e, h] Very low
**AZ vs.**						
SZ	-	-	1.49 (0.69 to 2.28)	⊕○○○ [i] Very low	1.49 (0.69 to 2.28)	⊕○○○ [i] Very low
MET	-	-	0.44 (-0.15 to 1.03)	⊕○○○ [i] Very low	0.44 (-0.15 to 1.03)	⊕○○○ [e, i] Very low
MOX	-	-	0.43 (-0.17 to 1.04)	⊕○○○ [i] Very low	0.43 (-0.17 to 1.04)	⊕○○○ [e, i] Very low
AMOX+MET	0.19 (-0.42 to 0.80)	⊕○○○ [a, c, d, e] Very low	0.38 (0.05 to 0.71)	⊕○○○ [i] Very low	0.38 (0.05 to 0.71)	⊕○○○ [i] Very low
CLM	-	-	0.39 (-0.25 to 1.04)	⊕○○○ [i] Very low	0.39 (-0.25 to 1.04)	⊕○○○ [e, i] Very low
CEF	-	-	0.13 (-0.63 to 0.89)	⊕○○○ [i] Very low	0.13 (-0.63 to 0.89)	⊕○○○ [e, i] Very low
SEC	-	-	0.03 (-0.85 to 0.91)	⊕○○○ [i] Very low	0.03 (-0.85 to 0.91)	⊕○○○ [e, i] Very low
MINO HCl	-	-	0.02 (-0.66 to 0.70)	⊕○○○ [i] Very low	0.02 (-0.66 to 0.70)	⊕○○○ [e, i] Very low
DOX	-	-	-0.17 (-0.94 to 0.60)	⊕○○○ [i] Very low	-0.17 (-0.94 to 0.60)	⊕○○○ [e, i] Very low
SD alone	-0.14 (-0.41 to 0.13)	⊕○○○ [a, c, d, e] Very low	-0.66 (-1.80 to 0.48)	⊕○○○ [i] Very low	-0.17 (-0.43 to 0.09)	⊕○○○ [e, i] Very low
**DOX vs.**						
SZ	-	-	1.66 (0.61 to 2.71)	⊕⊕○○ [h] Low	1.66 (0.61 to 2.71)	⊕⊕○○ [h] Low
MET	0.48 (-0.37 to 1.33)	⊕⊕○○ [a, e] Low	-1.34 (-3.31 to 0.63)	⊕⊕○○ [h] Low	0.61 (-0.16 to 1.39)	⊕○○○ [e, h] Very low
MOX	-	-	0.61 (-0.30 to 1.51)	⊕⊕○○ [h] Low	0.61 (-0.30 to 1.51)	⊕○○○ [e, h] Very low
AMOX+MET	0.43 (-0.43 to 1.29)	⊕⊕⊕○ [a] Moderate	0.86 (-0.51 to 2.24)	⊕○○○ [i] Very low	0.55 (-0.18 to 1.28)	⊕○○○ [e, i] Very low
CLM	-	-	0.57 (-0.37 to 1.50)	⊕○○○ [i] Very low	0.57 (-0.37 to 1.50)	⊕○○○ [e, i] Very low

Comparisons	GRADE					
	Direct evidence		Indirect evidence		Network meta-analysis	
	MD (95% CI)	Certainty of evidence	MD (95% CI)	Certainty of evidence	MD (95% CI)	Certainty of evidence
CEF	-	-	0.30 (-0.71 to 1.31)	⨁◯◯◯ [i]<br>Very low	0.30 (-0.71 to 1.31)	⨁◯◯◯ [e, i]<br>Very low
SEC	-	-	0.20 (-0.91 to 1.32)	⨁⨁◯◯ [h]<br>Low	0.20 (-0.91 to 1.32)	⨁◯◯◯ [e, h]<br>Very low
MINO HCl	-	-	0.19 (-0.77 to 1.15)	⨁⨁◯◯ [h]<br>Low	0.19 (-0.77 to 1.15)	⨁◯◯◯ [e, h]<br>Very low
AZ	-	-	0.17 (-0.60 to 0.94)	⨁◯◯◯ [i]<br>Very low	0.17 (-0.60 to 0.94)	⨁◯◯◯ [e, i]<br>Very low
SD alone	0.26 (-0.59 to 1.11)	⨁⨁◯◯ [a, e]<br>Low	-0.69 (-2.10 to 0.71)	⨁⨁◯◯ [h]<br>Low	0.00 (-0.73 to 0.73)	⨁◯◯◯ [e, h]<br>Very low
**SD alone vs.**						
SZ	1.66 (0.91 to 2.41)	⨁⨁◯◯ [b]<br>Low	-	-	1.66 (0.91 to 2.41)	⨁⨁◯◯ [h]<br>Low
MET	0.80 (0.19 to 1.40)	⨁⨁◯◯ [a, e]<br>Low	-0.12 (-1.31 to 1.07)	⨁⨁◯◯ [h]<br>Low	0.61 (0.07 to 1.15)	⨁⨁◯◯ [h]<br>Low
MOX	0.71 (-0.12 to 1.29)	⨁⨁◯◯ [a, d]<br>Low	-0.10 (-1.62 to 1.43)	⨁⨁◯◯ [h]<br>Low	0.60 (0.06 to 1.15)	⨁⨁◯◯ [h]<br>Low
AMOX+MET	0.56 (0.32 to 0.81)	⨁◯◯◯ [a, c, d, e, f]<br>Very low	0.27 (-0.79 to 1.33)	⨁◯◯◯ [i]<br>Very low	0.55 (0.31 to 0.79)	⨁◯◯◯ [i]<br>Very low
CLM	0.56 (0.02 to 1.15)	⨁◯◯◯ [a, c, d, e]<br>Very low	-	-	0.56 (-0.02 to 1.15)	⨁◯◯◯ [e, i]<br>Very low
CEF	0.04 (-0.78 to 0.86)	⨁◯◯◯ [b, e]<br>Very low	1.17 (-0.33 to 2.68)	⨁◯◯◯ [i]<br>Very low	0.30 (-0.42 to 1.02)	⨁◯◯◯ [e, i]<br>Very low
SEC	0.20 (-0.64 to 1.04)	⨁⨁◯◯ [a, e]<br>Low	-	-	0.20 (-0.64 to 1.04)	⨁◯◯◯ [e, h]<br>Very low
MINO HCl	0.18 (-0.44 to 0.81)	⨁⨁◯◯ [a, e]<br>Low	-	-	0.18 (-0.44 to 0.18)	⨁◯◯◯ [e, h]<br>Very low
AZ	0.14 (-0.13 to 0.41)	⨁◯◯◯ [a, c, d, e]<br>Very low	-0.66 (-1.80 to 0.48)	⨁◯◯◯ [i]<br>Very low	0.17 (-0.09 to 0.43)	⨁◯◯◯ [e, i]<br>Very low
DOX	0.26 (-1.11 to 0.59)	⨁⨁◯◯ [a, e]<br>Low	0.69 (-0.71 to 2.10)	⨁⨁◯◯ [h]<br>Low	0.00 (-0.73 to 0.73)	⨁◯◯◯ [e, h]<br>Very low

[a] serious risk of bias; [b] very serious risk of bias; [c] serious inconsistency; [d] serious indirectness [e] serious imprecision; [f] publication bias; [g] contributing evidence of moderate quality; [h] contributing evidence of low quality; [i] contributing evidence of very low quality; [j] contributing evidence of high quality; [k] incoherence

Table S28. Certainty of evidence for intermediate-term PPD reduction following the use of systemic antibiotics as adjunct to subgingival debridement

Comparisons	GRADE					
	Direct evidence		Indirect evidence		Network meta-analysis	
	MD (95% CI)	Certainty of evidence	MD (95% CI)	Certainty of evidence	MD (95% CI)	Certainty of evidence
**SZ vs.**						
MET	-	-	-1.43 (-2.26 to -0.60)	⊕⊕◯◯ [h] Low	-1.43 (-2.26 to -0.60)	⊕⊕◯◯ [h] Low
Clindamycin	-	-	-1.52 (-2.44 to -0.60)	⊕⊕◯◯ [h] Low	-1.52 (-2.44 to -0.60)	⊕⊕◯◯ [h] Low
AMOX+MET	-	-	-1.77 (-2.55 to -1.00)	⊕◯◯◯ [i] Very low	-1.77 (-2.55 to -1.00)	⊕◯◯◯ [i] Very low
MOX	-	-	-1.84 (-2.66 to -1.02)	⊕⊕◯◯ [h] Low	-1.84 (-2.66 to -1.02)	⊕⊕◯◯ [h] Low
DOX	-	-	-2.21 (-3.05 to -1.37)	⊕◯◯◯ [i] Very low	-2.21 (-3.05 to -1.37)	⊕◯◯◯ [i] Very low
MINO HCl	-	-	-2.22 (-3.20 to -1.24)	⊕⊕◯◯ [h] Low	-2.22 (-3.20 to -1.24)	⊕⊕◯◯ [h] Low
AZ	-	-	-2.27 (-3.05 to -1.49)	⊕◯◯◯ [i] Very low	-2.27 (-3.05 to -1.49)	⊕◯◯◯ [i] Very low
CLM	-	-	-2.36 (-3.17 to -1.55)	⊕◯◯◯ [i] Very low	-2.36 (-3.17 to -1.55)	⊕◯◯◯ [i] Very low
SD alone	-2.42 (-3.18 to -1.66)	⊕⊕◯◯ [b] Low	-	-	-2.42 (-3.18 to -1.66)	⊕⊕◯◯ [h] Low
**MET vs.**						
SZ	-	-	1.43 (0.60 to 2.26)	⊕⊕◯◯ [h] Low	1.43 (0.60 to 2.26)	⊕⊕◯◯ [h] Low
Clindamycin	0.00 (-0.55 to 0.55)	⊕⊕◯◯ [a, e] Low	-0.62 (-1.98 to 0.75)	⊕⊕◯◯ [h] Low	-0.09 (-0.60 to 0.43)	⊕◯◯◯ [e, h] Very low
AMOX+MET	-0.34 (-0.89 to 0.21)	⊕⊕◯◯ [a, e] Low	-0.35 (-0.85 to 0.16)	⊕◯◯◯ [i] Very low	-0.34 (-0.71 to 0.03)	⊕◯◯◯ [e, i] Very low
MOX	-	-	-0.41 (-0.89 to 0.06)	⊕⊕◯◯ [h] Low	-0.41 (-0.89 to 0.06)	⊕◯◯◯ [e, h] Very low
DOX	-0.78 (-1.15 to -0.41)	⊕◯◯◯ [a, c, d] Very low	-	-	-0.78 (-1.15 to -0.41)	⊕◯◯◯ [i] Very low
MINO HCl	-	-	-0.79 (-1.50 to -0.08)	⊕⊕◯◯ [h] Low	-0.79 (-1.50 to -0.08)	⊕⊕◯◯ [h] Low
AZ	-	-	-0.84 (-1.24 to -0.44)	⊕◯◯◯ [i] Very low	-0.84 (-1.24 to -0.44)	⊕◯◯◯ [i] Very low

Comparisons	GRADE					
	Direct evidence		Indirect evidence		Network meta-analysis	
	MD (95% CI)	Certainty of evidence	MD (95% CI)	Certainty of evidence	MD (95% CI)	Certainty of evidence
CLM	-	-	-0.93 (-1.39 to -0.47)	⊕◯◯◯ [i] Very low	-0.93 (-1.39 to -0.47)	⊕◯◯◯ [i] Very low
SD alone	-1.04 (-1.41 to -0.66)	⊕⊕◯◯ [a, d] Low	-0.71 (-1.66 to 0.24)	⊕⊕◯◯ [h] Low	-0.99 (-1.34 to -0.64)	⊕⊕◯◯ [h] Low
**Clindamycin vs.**						
SZ	-	-	1.52 (0.60 to 2.44)	⊕⊕◯◯ [h] Low	1.52 (0.60 – 2.44)	⊕⊕◯◯ [h] Low
MET	0.00 (-0.55 to 0.55)	⊕⊕◯◯ [a, e] Low	0.62 (-0.75 to 1.98)	⊕⊕◯◯ [h] Low	0.09 (-0.43 to 0.60)	⊕◯◯◯ [e, h] Very low
AMOX+MET	-	-	-0.26 (-0.79 to 0.28)	⊕◯◯◯ [i] Very low	-0.26 (-0.79 to 0.28)	⊕◯◯◯ [e, i] Very low
MOX	-	-	-0.32 (-0.93 to 0.29)	⊕⊕⊕◯ [g] Moderate	-0.32 (-0.93 to 0.29)	⊕⊕◯◯ [e, g] Low
DOX	-0.60 (-1.16 to -0.04)	⊕⊕⊕◯ [a] Moderate	-1.21 (-2.55 to 0.13)	⊕◯◯◯ [i] Very low	-0.69 (-1.21 to -0.17)	⊕◯◯◯ [i] Very low
MINO HCl	-	-	-0.70 (-1.51 to 0.10)	⊕⊕⊕◯ [g] Moderate	-0.70 (-1.51 to 0.10)	⊕⊕◯◯ [e, g] Low
AZ	-	-	-0.75 (-1.31 to -0.20)	⊕◯◯◯ [i] Very low	-0.75 (-1.31 to -0.20)	⊕◯◯◯ [i] Very low
CLM	-	-	-0.84 (-1.44 to -0.24)	⊕◯◯◯ [i] Very low	-0.84 (-1.44 to -0.24)	⊕◯◯◯ [i] Very low
SD alone	-0.90 (-1.49 to -0.31)	⊕⊕⊕◯ [a] Moderate	-0.91 (-2.04 to 0.21)	⊕⊕⊕◯ [g] Moderate	-0.90 (-1.42 to -0.38)	⊕⊕⊕◯ [g] Moderate
**AMOX+MET vs.**						
SZ	-	-	1.77 (1.00 to 2.55)	⊕◯◯◯ [i] Very low	1.77 (1.00 to 2.55)	⊕◯◯◯ [i] Very low
MET	0.34 (-0.21 to 0.89)	⊕⊕◯◯ [a, e] Low	-0.35 (-0.85 to 0.16)	⊕◯◯◯ [i] Very low	0.34 (-0.03 to 0.71)	⊕◯◯◯ [e, i] Very low
Clindamycin	-	-	0.26 (-0.28 to 0.79)	⊕◯◯◯ [i] Very low	0.26 (-0.28 to 0.79)	⊕◯◯◯ [e, i] Very low
MOX	-0.07 (-0.57 to 0.43)	⊕⊕◯◯ [a, e] Low	-0.07 (-0.54 to 0.41)	⊕◯◯◯ [i] Very low	-0.26 (-0.79 to 0.28)	⊕◯◯◯ [e, h] Very low
DOX	-0.62 (-1.18 to -0.06)	⊕⊕⊕◯ [a] Moderate	-0.32 (-0.93 to 0.29)	⊕◯◯◯ [i] Very low	-0.32 (-0.93 to 0.29)	⊕⊕◯◯ [e, g] Low

Comparisons	GRADE					
	Direct evidence		Indirect evidence		Network meta-analysis	
	MD (95% CI)	Certainty of evidence	MD (95% CI)	Certainty of evidence	MD (95% CI)	Certainty of evidence
MINO HCl	-	-	-0.69 (-1.21 to -0.17)	⨁◯◯◯ [i] Very low	-0.69 (-1.21 to -0.17)	⨁◯◯◯ [i] Very low
AZ	-	-	-0.70 (-1.51 to 0.10)	⨁◯◯◯ [i] Very low	-0.70 (-1.51 to 0.10)	⨁◯◯◯ [e, i] Very low
CLM	-	-	-0.75 (-1.31 to -0.20)	⨁◯◯◯ [i] Very low	-0.75 (-1.31 to -0.20)	⨁◯◯◯ [i] Very low
SD alone	-0.66 (-0.84 to -0.49)	⨁◯◯◯ [a, c, d] Very low	-0.22 (-1.13 to 0.70)	⨁◯◯◯ [i] Very low	-0.84 (-1.44 to -0.24)	⨁◯◯◯ [i] Very low
**MOX vs.**						
SZ	-	-	1.84 (1.02 to 2.66)	⨁⨁◯◯ [h] Low	1.84 (1.02 to 2.66)	⨁⨁◯◯ [h] Low
MET	-	-	0.41 (-0.06 to 0.89)	⨁⨁◯◯ [h] Low	0.41 (-0.06 to 0.89)	⨁◯◯◯ [e, h] Very low
Clindamycin	-	-	0.32 (-0.29 to 0.93)	⨁⨁⨁◯ [g] Moderate	0.32 (-0.29 to 0.93)	⨁⨁◯◯ [e, g] Low
AMOX+MET	0.07 (-0.43 to 0.57)	⨁⨁◯◯ [a, e] Low	0.07 (-0.41 to 0.54)	⨁◯◯◯ [i] Very low	0.07 (-0.28 to 0.79)	⨁◯◯◯ [e, h] Very low
DOX	-	-	-0.37 (-0.85 to 0.11)	⨁◯◯◯ [i] Very low	-0.37 (-0.85 to 0.11)	⨁◯◯◯ [e, i] Very low
MINO HCl	-	-	-0.38 (-1.08 to 0.32)	⨁⨁⨁◯ [g] Moderate	-0.38 (-1.08 to 0.32)	⨁⨁◯◯ [e, g] Low
AZ	-	-	-0.43 (-0.80 to -0.06)	⨁◯◯◯ [i] Very low	-0.43 (-0.80 to -0.06)	⨁◯◯◯ [i] Very low
CLM	-	-	-0.52 (-0.96 to -0.08)	⨁◯◯◯ [i] Very low	-0.52 (-0.96 to -0.08)	⨁◯◯◯ [i] Very low
SD alone	-0.64 (-0.99 to -0.30)	⨁⨁⨁◯ [c] Moderate	-0.13 (-1.06 to 0.80)	⨁⨁⨁◯ [g] Moderate	-0.58 (-0.90 to -0.25)	⨁⨁◯◯ [e, g] Low
**DOX vs.**						
SZ	-	-	2.21 (1.37 to 3.05)	⨁◯◯◯ [i] Very low	2.21 (1.37 to 3.05)	⨁◯◯◯ [i] Very low
MET	0.78 (0.41 to 1.15)	⨁◯◯◯ [a, c, d] Very low	-	-	0.78 (0.41 to 1.15)	⨁◯◯◯ [i] Very low

Comparisons	GRADE					
	Direct evidence		Indirect evidence		Network meta-analysis	
	MD (95% CI)	Certainty of evidence	MD (95% CI)	Certainty of evidence	MD (95% CI)	Certainty of evidence
Clindamycin	0.60 (0.04 to 1.16)	⊕◯◯◯ [a, d, e]<br>Very low	1.21 (-0.13 to 2.55)	⊕◯◯◯ [i]<br>Very low	0.69 (0.17 to 1.21)	⊕◯◯◯ [i]<br>Very low
AMOX+MET	0.62 (0.06 to 1.18)	⊕◯◯◯ [a, c, d]<br>Very low	0.28 (-0.23 to 0.78)	⊕◯◯◯ [i]<br>Very low	0.44 (0.06 to 0.81)	⊕◯◯◯ [i]<br>Very low
MOX	-	-	0.37 (-0.11 to 0.85)	⊕◯◯◯ [i]<br>Very low	0.37 (-0.11 to 0.85)	⊕◯◯◯ [e, i]<br>Very low
MINO HCl	-	-	-0.01 (-0.73 to 0.70)	⊕◯◯◯ [i]<br>Very low	-0.01 (-0.73 to 0.70)	⊕◯◯◯ [e, i]<br>Very low
AZ	-	-	-0.06 (-0.47 to 0.34)	⊕◯◯◯ [i]<br>Very low	-0.06 (-0.47 to 0.34)	⊕◯◯◯ [e, i]<br>Very low
CLM	-	-	-0.15 (-0.62 to 0.31)	⊕◯◯◯ [i]<br>Very low	-0.15 (-0.62 to 0.31)	⊕◯◯◯ [e, i]<br>Very low
SD alone	-0.25 (-0.64 to 0.14)	⊕◯◯◯ [a, d, e]<br>Very low	0.03 (-0.97 to 1.02)	⊕◯◯◯ [i]<br>Very low	-0.21 (-0.57 to 0.15)	⊕◯◯◯ [e, i]<br>Very low
**MINO HCl vs.**						
SZ	-	-	2.22 (1.24 to 3.20)	⊕⊕◯◯ [h]<br>Low	2.22 (1.24 to 3.20)	⊕⊕◯◯ [h]<br>Low
MET	-	-	0.79 (0.08 to 1.50)	⊕⊕◯◯ [h]<br>Low	0.79 (0.08 to 1.50)	⊕⊕◯◯ [h]<br>Low
Clindamycin	-	-	0.70 (-0.10 to 1.51)	⊕⊕⊕◯ [g]<br>Moderate	0.70 (-0.10 to 1.51)	⊕⊕◯◯ [e, g]<br>Low
AMOX+MET	-	-	0.45 (-0.19 to 1.09)	⊕◯◯◯ [i]<br>Very low	0.45 (-0.19 to 1.09)	⊕◯◯◯ [e, i]<br>Very low
MOX	-	-	0.38 (-0.32 to 1.08)	⊕⊕⊕◯ [g]<br>Moderate	0.38 (-0.32 to 1.08)	⊕⊕◯◯ [e, g]<br>Low
DOX	-	-	0.01 (-0.70 to 0.73)	⊕◯◯◯ [i]<br>Very low	0.01 (-0.70 to 0.73)	⊕◯◯◯ [e, i]<br>Very low
AZ	-	-	-0.05 (-0.69 to 0.59)	⊕◯◯◯ [i]<br>Very low	-0.05 (-0.69 to 0.59)	⊕◯◯◯ [e, i]<br>Very low
CLM	-	-	-0.14 (-0.82 to 0.54)	⊕◯◯◯ [i]<br>Very low	-0.14 (-0.82 to 0.54)	⊕◯◯◯ [e, i]<br>Very low
SD alone	-0.20 (-0.82 to 0.42)	⊕⊕⊕◯ [e]<br>Moderate	-	-	-0.20 (-0.82 to 0.42)	⊕⊕◯◯ [e, g]<br>Low

Comparisons	GRADE					
	Direct evidence		Indirect evidence		Network meta-analysis	
	MD (95% CI)	Certainty of evidence	MD (95% CI)	Certainty of evidence	MD (95% CI)	Certainty of evidence
**AZ vs.**						
SZ	-	-	2.27 (1.49 to 3.05)	⊕◯◯◯ [i] Very low	2.27 (1.49 to 3.05)	⊕◯◯◯ [i] Very low
MET	-	-	0.84 (0.44 to 1.24)	⊕◯◯◯ [i] Very low	0.84 (0.44 to 1.24)	⊕◯◯◯ [i] Very low
Clindamycin	-	-	0.75 (0.20 to 1.31)	⊕◯◯◯ [i] Very low	0.75 (0.20 to 1.31)	⊕◯◯◯ [i] Very low
AMOX+MET	-	-	0.50 (0.25 to 0.75)	⊕◯◯◯ [i] Very low	0.50 (0.25 to 0.75)	⊕◯◯◯ [i] Very low
MOX	-	-	0.43 (0.06 to 0.80)	⊕◯◯◯ [i] Very low	0.43 (0.06 to 0.80)	⊕◯◯◯ [i] Very low
DOX	-	-	0.06 (-0.34 to 0.47)	⊕◯◯◯ [i] Very low	0.06 (-0.34 to 0.47)	⊕◯◯◯ [e, i] Very low
MINO HCl	-	-	0.05 (-0.59 to 0.69)	⊕◯◯◯ [i] Very low	0.05 (-0.59 to 0.69)	⊕◯◯◯ [e, i] Very low
CLM	-	-	-0.09 (-0.44 to 0.26)	⊕◯◯◯ [i] Very low	-0.09 (-0.44 to 0.26)	⊕◯◯◯ [e, i] Very low
SD alone	-0.15 (-0.33 to 0.03)	⊕◯◯◯ [a, c, d, e] Very low	-	-	-0.15 (-0.33 to 0.03)	⊕◯◯◯ [e, i] Very low
**CLM vs.**						
SZ	-	-	2.36 (1.55 to 3.17)	⊕◯◯◯ [i] Very low	2.36 (1.55 to 3.17)	⊕◯◯◯ [i] Very low
MET	-	-	0.93 (0.47 to 1.39)	⊕◯◯◯ [i] Very low	0.93 (0.47 to 1.39)	⊕◯◯◯ [i] Very low
Clindamycin	-	-	0.84 (0.24 to 1.44)	⊕◯◯◯ [i] Very low	0.84 (0.24 to 1.44)	⊕◯◯◯ [i] Very low
AMOX+MET	-	-	0.59 (0.25 to 0.93)	⊕◯◯◯ [i] Very low	0.59 (0.25 to 0.93)	⊕◯◯◯ [i] Very low
MOX	-	-	0.52 (0.08 to 0.96)	⊕◯◯◯ [i] Very low	0.52 (0.08 to 0.96)	⊕◯◯◯ [i] Very low
DOX	-	-	0.15 (-0.31 to 0.62)	⊕◯◯◯ [i] Very low	0.15 (-0.31 to 0.62)	⊕◯◯◯ [e, i] Very low
MINO HCl	-	-	0.14 (-0.54 to 0.82)	⊕◯◯◯ [i] Very low	0.14 (-0.54 to 0.82)	⊕◯◯◯ [e, i] Very low

Comparisons	GRADE					
	Direct evidence		Indirect evidence		Network meta-analysis	
	MD (95% CI)	Certainty of evidence	MD (95% CI)	Certainty of evidence	MD (95% CI)	Certainty of evidence
AZ	-	-	0.09 (-0.26 to 0.44)	⊕○○○ [i] Very low	0.09 (-0.26 to 0.44)	⊕○○○ [e, i] Very low
SD alone	-0.06 (-0.36 to 0.24)	⊕○○○ [c, d, e] Very low	-	-	-0.06 (-0.36 to 0.24)	⊕○○○ [e, i] Very low
**SD alone vs.**						
SZ	2.42 (1.66 to 3.18)	⊕⊕○○ [b] Low	-	-	2.42 (1.66 to 3.18)	⊕⊕○○ [h] Low
MET	1.04 (0.66 to 1.41)	⊕⊕○○ [a, d] Low	0.71 (-0.24 to 1.66)	⊕⊕○○ [h] Low	0.99 (0.64 to 1.34)	⊕⊕○○ [h] Low
Clindamycin	0.90 (0.31 to 1.49)	⊕⊕⊕○ [a] Moderate	1.21 (-0.13 to 2.55)	⊕⊕⊕○ [g] Moderate	0.90 (0.38 to 1.42)	⊕⊕⊕○ [g] Moderate
AMOX+MET	0.66 (0.49 to 0.84)	⊕○○○ [a, c, d] Very low	0.22 (-0.70 to 1.13)	⊕○○○ [i] Very low	0.65 (0.48 to 0.82)	⊕○○○ [i] Very low
MOX	0.64 (0.30 to 0.99)	⊕⊕⊕○ [a] Moderate	0.13 (-0.80 to 1.06)	⊕⊕⊕○ [g] Moderate	0.58 (0.25 to 0.90)	⊕⊕⊕○ [g] Moderate
DOX	0.25 (-0.14 to 0.64)	⊕○○○ [a, d, e] Very low	-0.03 (-1.02 to 0.97)	⊕○○○ [i] Very low	0.21 (-0.15 to 0.57)	⊕○○○ [e, i] Very low
MINO HCl	0.20 (-0.42 to 0.82)	⊕⊕⊕○ [e] Moderate	-	-	0.20 (-0.42 to 0.82)	⊕⊕○○ [e, g] Low
AZ	0.15 (-0.03 to 0.33)	⊕○○○ [a, c, d, e] Very low	-	-	0.15 (-0.03 to 0.33)	⊕○○○ [e, i] Very low
CLM	0.06 (-0.24 to 0.36)	⊕○○○ [c, d, e] Very low	-	-	0.06 (-0.24 to 0.36)	⊕○○○ [e, i] Very low

[a] serious risk of bias; [b] very serious risk of bias; [c] serious inconsistency; [d] serious indirectness [e] serious imprecision; [f] publication bias; [g] contributing evidence of moderate quality; [h] contributing evidence of low quality; [i] contributing evidence of very low quality; [j] contributing evidence of high quality; [k] incoherence

Table S29. Certainty of evidence for long-term PPD reduction following the use of systemic antibiotics as adjunct to subgingival debridement

Comparisons	GRADE					
	Direct evidence		Indirect evidence		Network meta-analysis	
	MD (95% CI)	Certainty of evidence	MD (95% CI)	Certainty of evidence	MD (95% CI)	Certainty of evidence
**MET vs.**						
Clindamycin	- -0.30 (-0.62 to 0.02)	⊕⊕○○ [a, e] Low	-	-	-0.30 (-0.62 to 0.02)	⊕○○○ [e, i] Very low

Comparisons	GRADE					
	Direct evidence		Indirect evidence		Network meta-analysis	
	MD (95% CI)	Certainty of evidence	MD (95% CI)	Certainty of evidence	MD (95% CI)	Certainty of evidence
DOX	-1.30 (-1.60 to -1.00)	⊕⊕⊕◯ [a] Moderate	-	-	-1.30 (-1.60 to -1.00)	⊕⊕⊕◯ [g] Moderate
AMOX+MET	-	-	-1.41 (-1.72 to -1.09)	⊕⊕◯◯ [h] Low	-1.41 (-1.72 to -1.09)	⊕⊕◯◯ [h] Low
AZ	-	-	-1.84 (-2.22 to -1.46)	⊕◯◯◯ [i] Very low	-1.84 (-2.22 to -1.46)	⊕◯◯◯ [i] Very low
SD alone	-1.90 (-2.20 to -1.60)	⊕⊕⊕◯ [a] Moderate	-	-	-1.90 (-2.20 to -1.60)	⊕⊕⊕◯ [g] Moderate
**Clindamycin vs.**						
MET	0.30 (-0.02 to 0.62)	⊕⊕◯◯ [a, e] Low	-	-	0.30 (-0.02 to 0.62)	⊕◯◯◯ [e, i] Very low
DOX	-1.00 (-1.36 to -0.64)	⊕⊕⊕◯ [a] Moderate	-	-	-1.00 (-1.36 to -0.64)	⊕⊕⊕◯ [g] Moderate
AMOX+MET	-	-	-1.11 (-1.48 to -0.74)	⊕⊕◯◯ [h] Low	-1.11 (-1.48 to -0.74)	⊕⊕◯◯ [h] Low
AZ	-	-	-1.54 (-1.97 to -1.11)	⊕◯◯◯ [i] Very low	-1.54 (-1.97 to -1.11)	⊕◯◯◯ [i] Very low
SD alone	-1.60 (-1.96 to -1.24)	⊕⊕⊕◯ [a] Moderate	-	-	-1.60 (-1.96 to -1.24)	⊕⊕⊕◯ [g] Moderate
**DOX vs.**						
MET	1.30 (1.00 to 1.60)	⊕⊕⊕◯ [a] Moderate	-	-	1.30 (1.00 to 1.60)	⊕⊕⊕◯ [g] Moderate
Clindamycin	1.00 (0.64 to 1.36)	⊕⊕⊕◯ [a] Moderate	-	-	1.00 (0.64 to 1.36)	⊕⊕⊕◯ [g] Moderate
AMOX+MET	-	-	-0.11 (-0.46 to 0.24)	⊕⊕◯◯ [h] Low	-0.11 (-0.46 to 0.24)	⊕◯◯◯ [e, h] Very low
AZ	-	-	-0.54 (-0.95 to -0.13)	⊕◯◯◯ [i] Very low	-0.54 (-0.95 to -0.13)	⊕◯◯◯ [i] Very low
SD alone	-0.60 (-0.94 to -0.26)	⊕⊕⊕◯ [a] Moderate	-	-	-0.60 (-0.94 to -0.26)	⊕⊕⊕◯ [g] Moderate
**AMOX+MET vs.**						
MET	-	-	1.41 (1.09 to 1.72)	⊕⊕◯◯ [h] Low	1.41 (1.09 to 1.72)	⊕⊕◯◯ [h] Low
Clindamycin	-	-	1.11 (0.74 to 1.48)	⊕⊕◯◯ [h] Low	1.11 (0.74 to 1.48)	⊕⊕◯◯ [h] Low

Comparisons	GRADE					
	Direct evidence		Indirect evidence		Network meta-analysis	
	MD (95% CI)	Certainty of evidence	MD (95% CI)	Certainty of evidence	MD (95% CI)	Certainty of evidence
DOX	-	-	0.11 (-0.24 to 0.46)	⊕⊕◯◯ [h] Low	0.11 (-0.24 to 0.46)	⊕◯◯◯ [e, h] Very low
AZ	-	-	-0.43 (-0.68 to -0.18)	⊕◯◯◯ [i] Very low	-0.43 (-0.68 to -0.18)	⊕◯◯◯ [i] Very low
SD alone	-0.49 (-0.58 to -0.40)	⊕⊕◯◯ [a, d] Low	-	-	-0.49 (-0.58 to -0.40)	⊕⊕◯◯ [h] Low
**AZ vs.**						
MET	-	-	1.84 (1.46 to 2.22)	⊕◯◯◯ [i] Very low	1.84 (1.46 to 2.22)	⊕◯◯◯ [i] Very low
Clindamycin	-	-	1.54 (1.11 to 1.97)	⊕◯◯◯ [i] Very low	1.54 (1.11 to 1.97)	⊕◯◯◯ [i] Very low
DOX	-	-	0.54 (0.13 to 0.95)	⊕◯◯◯ [i] Very low	0.54 (0.13 to 0.95)	⊕◯◯◯ [i] Very low
AMOX+MET	-	-	0.43 (0.18 to 0.68)	⊕◯◯◯ [i] Very low	0.43 (0.18 to 0.68)	⊕◯◯◯ [i] Very low
SD alone	-0.06 (-0.30 to 0.17)	⊕◯◯◯ [a, c, d, e] Very low	-	-	-0.06 (-0.30 to 0.17)	⊕◯◯◯ [e, i] Very low
**SD alone vs.**						
MET	1.90 (1.60 to 2.20)	⊕⊕⊕◯ [a] Moderate	-	-	1.90 (1.60 to 2.20)	⊕⊕⊕◯ [g] Moderate
Clindamycin	1.60 (1.24 to 1.96)	⊕⊕⊕◯ [a] Moderate	-	-	1.60 (1.24 to 1.96)	⊕⊕⊕◯ [g] Moderate
DOX	0.60 (0.26 to 0.94)	⊕⊕⊕◯ [a] Moderate	-	-	0.60 (0.26 to 0.94)	⊕⊕⊕◯ [g] Moderate
AMOX+MET	0.49 (0.40 to 0.58)	⊕⊕◯◯ [a, d] Low	-	-	0.49 (0.40 to 0.58)	⊕⊕◯◯ [h] Low
AZ	0.06 (-0.17 to .30)	⊕◯◯◯ [a, c, d, e] Very low	-	-	0.6 (-0.17 to 0.30)	⊕◯◯◯ [e, i] Very low

[a] serious risk of bias; [b] very serious risk of bias; [c] serious inconsistency; [d] serious indirectness [e] serious imprecision; [f] publication bias; [g] contributing evidence of moderate quality; [h] contributing evidence of low quality; [i] contributing evidence of very low quality; [j] contributing evidence of high quality; [k] incoherence

Table S30. Certainty of evidence for short-term BOP reduction following the use of systemic antibiotics as adjunct to subgingival debridement

Comparisons	GRADE					
	Direct evidence		Indirect evidence		Network meta-analysis	
	MD (95% CI)	Certainty of evidence	MD (95% CI)	Certainty of evidence	MD (95% CI)	Certainty of evidence
**MET vs.**						
AMOX+MET	-16.20 (-27.05 to -5.35)	⊕⊕⊕◯ [e] Moderate	-5.05 (-25.45 to 15.35)	⊕◯◯◯ [i] Very low	-13.74 (-23.32 to -4.16)	⊕◯◯◯ [i] Very low
AZ	-	-	-18.39 (-28.74 to -8.04)	⊕◯◯◯ [i] Very low	-18.39 (-28.74 to -8.04)	⊕◯◯◯ [i] Very low
MOX	-	-	-19.38 (-30.80 to -7.96)	⊕⊕⊕◯ [g] Moderate	-19.38 (-30.80 to -7.96)	⊕⊕⊕◯ [g] Moderate
CLM	-	-	-20.06 (-34.15 to -5.98)	⊕⊕⊕◯ [g] Moderate	-20.06 (-34.15 to -5.98)	⊕⊕⊕◯ [g] Moderate
SD alone	-19.50 (-30.46 to -8.54)	⊕⊕⊕◯ [e] Moderate	-30.42 (-50.22 to -10.53)	⊕⊕⊕◯ [g] Moderate	-22.06 (-31.66 to -12.47)	⊕⊕⊕◯ [g] Moderate
**AMOX+MET vs.**						
MET	16.20 (5.35 to 27.05)	⊕⊕⊕◯ [e] Moderate	5.05 (-15.35 to 25.45)	⊕◯◯◯ [i] Very low	13.74 (4.16 to 23.32)	⊕◯◯◯ [i] Very low
AZ	-5.24 (-14.33 to 3.86)	⊕⊕◯◯ [a, e] Low	-4.37 (-10.57 to 1.83)	⊕◯◯◯ [i] Very low	-4.65 (-9.77 to 0.48)	⊕◯◯◯ [e, i] Very low
MOX	-0.60 (-10.34 to 9.14)	⊕⊕⊕◯ [e] Moderate	-10.71 (-20.49 to -0.94)	⊕◯◯◯ [i] Very low	-5.64 (-12.54 to 1.26)	⊕◯◯◯ [e, i] Very low
CLM	-	-	-6.32 (-17.24 to 4.59)	⊕◯◯◯ [i] Very low	-6.32 (-17.24 to 4.59)	⊕◯◯◯ [e, i] Very low
SD alone	-8.17 (-11.86 to -4.48)	⊕◯◯◯ [a, d, e] Very low	-10.89 (-25.85 to 4.07)	⊕◯◯◯ [i] Very low	-8.32 (-11.90 to -4.74)	⊕◯◯◯ [i] Very low
**AZ vs.**						
MET	-	-	18.39 (8.04 to 28.74)	⊕◯◯◯ [i] Very low	18.39 (8.04 to 28.74)	⊕◯◯◯ [i] Very low
AMOX+MET	5.24 (-3.86 to 14.33)	⊕⊕◯◯ [a, e] Low	4.37 (-1.83 to 10.57)	⊕◯◯◯ [i] Very low	4.65 (-0.48 to 9.77)	⊕◯◯◯ [e, i] Very low
MOX	-	-	-0.99 (-8.62 to 6.63)	⊕◯◯◯ [i] Very low	-0.99 (-8.62 to 6.63)	⊕◯◯◯ [e, i] Very low
CLM	-	-	-1.68 (-12.78 to 9.43)	⊕◯◯◯ [i] Very low	-1.68 (-12.78 to 9.43)	⊕◯◯◯ [e, i] Very low
SD alone	-3.36 (-7.61 to 0.89)	⊕◯◯◯ [a, c, d, e] Very low	-8.57 (-25.33 to 8.20)	⊕◯◯◯ [i] Very low	-3.68 (-7.80 to 0.44)	⊕◯◯◯ [e, i] Very low

Comparisons	GRADE					
	Direct evidence		Indirect evidence		Network meta-analysis	
	MD (95% CI)	Certainty of evidence	MD (95% CI)	Certainty of evidence	MD (95% CI)	Certainty of evidence
**MOX vs.**						
MET	-	-	19.38 (7.96 to 30.80)	⊕⊕⊕◯ [g] Moderate	19.38 (7.96 to 30.80)	⊕⊕⊕◯ [g] Moderate
AMOX+MET	0.60 (-9.14 to 10.34)	⊕⊕⊕◯ [e] Moderate	10.71 (0.94 to 20.49)	⊕◯◯◯ [i] Very low	5.64 (-1.26 to 12.54)	⊕◯◯◯ [e, i] Very low
AZ	-	-	0.99 (-6.63 to 8.62)	⊕◯◯◯ [i] Very low	0.99 (-6.63 to 8.62)	⊕◯◯◯ [e, i] Very low
CLM	-	-	-0.68 (-12.87 to 11.50)	⊕⊕⊕◯ [g] Moderate	-0.68 (-12.87 to 11.50)	⊕◯◯◯ [e, g] Very low
SD alone	-0.70 (-7.63 to 6.22)	⊕⊕⊕◯ [e] Moderate	-17.06 (-35.72 to 1.60)	⊕⊕⊕◯ [g] Moderate	-2.68 (-9.18 to 3.81)	⊕◯◯◯ [e, g] Very low
**CLM vs.**						
MET	-	-	20.06 (5.98 to 34.15)	⊕⊕⊕◯ [g] Moderate	20.06 (5.98 to 34.15)	⊕⊕⊕◯ [g] Moderate
AMOX+MET	-	-	6.32 (-4.59 to 17.24)	⊕◯◯◯ [i] Very low	6.32 (-4.59 to 17.24)	⊕◯◯◯ [e, i] Very low
AZ	-	-	1.68 (-9.43 to 12.78)	⊕◯◯◯ [i] Very low	1.68 (-9.43 to 12.78)	⊕◯◯◯ [e, i] Very low
MOX	-	-	2.68 (-11.50 to 12.87)	⊕⊕⊕◯ [g] Moderate	2.68 (-11.50 to 12.87)	⊕⊕◯◯ [e, g] Low
SD alone	-2.00 (-12.31 to 8.31)	⊕⊕⊕◯ [e] Moderate	-	-	-2.00 (-12.31 to 8.31)	⊕⊕◯◯ [e, g] Low
**SD alone vs.**						
MET	19.50 (8.54 to 30.46)	⊕⊕⊕◯ [e] Moderate	30.42 (10.63 to 50.22)	⊕⊕⊕◯ [g] Moderate	22.06 (12.47 to 31.66)	⊕⊕⊕◯ [g] Moderate
AMOX+MET	8.17 (4.48 to 11.86)	⊕◯◯◯ [a, d, e] Very low	10.89 (-4.07 to 25.85)	⊕◯◯◯ [i] Very low	8.32 (4.74 to 11.90)	⊕◯◯◯ [i] Very low
AZ	3.36 (-0.89 to 7.61)	⊕◯◯◯ [a, c, d, e] Very low	8.57 (-8.20 to 25.33)	⊕◯◯◯ [i] Very low	3.68 (-0.44 to 7.80)	⊕◯◯◯ [e, i] Very low
MOX	0.70 (-6.22 to 7.63)	⊕⊕⊕◯ [e] Moderate	17.06 (-1.60 to 35.72)	⊕⊕⊕◯ [g] Moderate	2.68 (-3.81 to 9.18)	⊕⊕◯◯ [e, g] Low
CLM	2.00 (-8.31 to 12.31)	⊕⊕⊕◯ [e] Moderate	-	-	2.00 (-8.31 to 12.31)	⊕⊕◯◯ [e, g] Low

[a] serious risk of bias; [b] very serious risk of bias; [c] serious inconsistency; [d] serious indirectness [e] serious imprecision; [f] publication bias; [g] contributing evidence of moderate quality; [h] contributing evidence of low quality; [i] contributing evidence of very low quality; [j] contributing evidence of high quality; [k] incoherence

Table S31. Certainty of evidence for intermediate-term BOP reduction following the use of systemic antibiotics as adjunct to subgingival debridement

Comparisons	GRADE					
	Direct evidence		Indirect evidence		Network meta-analysis	
	MD (95% CI)	Certainty of evidence	MD (95% CI)	Certainty of evidence	MD (95% CI)	Certainty of evidence
**AMOX+MET vs.**						
AZ	-	-	-4.92 (-11.38 to 1.54)	⊕○○○ [i] Very low	-4.92 (-11.38 to 1.54)	⊕○○○ [e, i] Very low
CLM	-	-	-6.19 (-14.60 to 2.22)	⊕⊕○○ [h] Low	-6.19 (-14.60 to 2.22)	⊕○○○ [e, h] Very low
MOX	-0.40 (-9.90 to 9.10)	⊕⊕⊕○ [e] Moderate	-13.56 (-23.82 to -3.31)	⊕⊕○○ [h] Low	-6.48 (-13.45 to 0.49)	⊕○○○ [e, h] Very low
SD alone	-8.48 (-12.86 to -4.10)	⊕⊕○○ [d, e] Low	5.09 (-20.20 to 30.38)	⊕⊕○○ [h] Low	-8.08 (-12.40 to -3.77)	⊕⊕○○ [h] Low
**AZ vs.**						
AMOX+MET	-	-	4.92 (-1.54 to 11.38)	⊕○○○ [i] Very low	4.92 (-1.54 to 11.38)	⊕○○○ [e, i] Very low
CLM	-	-	-1.27 (-9.94 to 7.40)	⊕○○○ [i] Very low	-1.27 (-9.94 to 7.40)	⊕○○○ [e, i] Very low
MOX	-	-	-1.56 (-9.55 to 6.43)	⊕○○○ [i] Very low	-1.56 (-9.55 to 6.43)	⊕○○○ [e, i] Very low
SD alone	-3.16 (-7.97 to 1.65)	⊕○○○ [a, c, d, e] Very low	-	-	-3.16 (-7.97 to 1.65)	⊕○○○ [e, i] Very low
**CLM vs.**						
AMOX+MET	-	-	6.19 (-2.22 to 14.60)	⊕⊕○○ [h] Low	6.19 (-2.22 to 14.60)	⊕○○○ [e, h] Very low
AZ	-	-	1.27 (-7.40 to 9.94)	⊕○○○ [i] Very low	1.27 (-7.40 to 9.94)	⊕○○○ [e, i] Very low
MOX	-	-	-0.29 (-9.92 to 9.34)	⊕⊕○○ [h] Low	-0.29 (-9.92 to 9.34)	⊕○○○ [e, h] Very low
SD alone	-1.89 (-9.11 to 5.32)	⊕⊕⊕○ [e] Moderate	-	-	-1.89 (-9.11 to 5.32)	⊕⊕○○ [e, g] Low
**MOX vs.**						
AMOX+MET	0.40 (-9.10 to 9.90)	⊕⊕⊕○ [e] Moderate	13.56 (3.31 to 23.82)	⊕⊕○○ [h] Low	6.48 (-0.49 to 13.45)	⊕○○○ [e, h] Very low
AZ	-	-	1.56 (-6.43 to 9.55)	⊕○○○ [i] Very low	1.56 (-6.43 to 9.55)	⊕○○○ [e, i] Very low
CLM	-	-	0.29 (-9.34 to 9.92)	⊕⊕○○ [h] Low	0.29 (-9.34 to 9.92)	⊕○○○ [e, h] Very low

Comparisons	GRADE					
	Direct evidence		Indirect evidence		Network meta-analysis	
	MD (95% CI)	Certainty of evidence	MD (95% CI)	Certainty of evidence	MD (95% CI)	Certainty of evidence
SD alone	0.53 (-6.23 to 7.29)	⊕⊕◯◯ [c, e] Low	-18.96 (-35.25 to 0.32)	⊕⊕◯◯ [h] Low	-1.60 (-7.98 to 4.78)	⊕◯◯◯ [e, h] Very low
**SD alone vs.**						
AMOX+MET	8.48 (4.10 to 12.86)	⊕⊕◯◯ [d, e] Low	-5.09 (-30.38 to 20.20)	⊕⊕◯◯ [h] Low	8.08 (3.77 to 12.40)	⊕⊕◯◯ [h] Low
AZ	3.16 (-1.65 to 7.97)	⊕◯◯◯ [a, c, d, e] Very low	-	-	3.16 (-1.65 to 7.95)	⊕◯◯◯ [e, i] Very low
CLM	1.89 (-5.32 to 9.11)	⊕⊕⊕◯ [e] Moderate	-	-	1.89 (-5.32 to 9.11)	⊕⊕◯◯ [e, g] Low
MOX	0.53 (-7.29 to 6.23)	⊕⊕◯◯ [c, e] Low	18.956 (-0.32 to 38.25)	⊕⊕◯◯ [h] Low	1.60 (-4.78 to 7.96)	⊕◯◯◯ [e, h] Very low

[a] serious risk of bias; [b] very serious risk of bias; [c] serious inconsistency; [d] serious indirectness [e] serious imprecision; [f] publication bias; [g] contributing evidence of moderate quality; [h] contributing evidence of low quality; [i] contributing evidence of very low quality; [j] contributing evidence of high quality; [k] incoherence

Table S32. Certainty of evidence for long-term BOP reduction following the use of systemic antibiotics as adjunct to subgingival debridement

Comparisons	GRADE					
	Direct evidence		Indirect evidence		Network meta-analysis	
	MD (95% CI)	Certainty of evidence	MD (95% CI)	Certainty of evidence	MD (95% CI)	Certainty of evidence
**AMOX+MET vs.**						
SD alone	-14.27 (-16.99 to -11.55)	⊕⊕⊕⊕ High	-	-	-14.27 (-16.99 to -11.55)	⊕⊕⊕⊕ [j] High
AZ	-	-	-17.78 (-22.67 to -12.89)	⊕◯◯◯ [i] Very low	-17.78 (-22.67 to -12.89)	⊕◯◯◯ [i] Very low
**SD alone vs.**						
AMOX+MET	14.27 (11.55 to 16.99)	⊕⊕⊕⊕ High	-	-	14.27 (11.55 to 16.99)	⊕⊕⊕⊕ [j] High
AZ	-3.51 (-7.58 to 0.56)	⊕◯◯◯ [a, c, d, e] Very low	-	-	-3.51 (-7.58 to 0.56)	⊕◯◯◯ [e, i] Very low
**AZ vs.**						
AMOX+MET	-	-	17.78 (12.89 to 22.67)	⊕◯◯◯ [i] Very low	17.78 (12.89 to 22.67)	⊕◯◯◯ [i] Very low
SD alone	3.51 (-0.56 to 7.58)	⊕◯◯◯ [a, c, d, e] Very low	-	-	3.51 (-0.56 to 7.58)	⊕◯◯◯ [e, i] Very low

[a] serious risk of bias; [b] very serious risk of bias; [c] serious inconsistency; [d] serious indirectness [e] serious imprecision; [f] publication bias; [g] contributing evidence of moderate quality; [h] contributing evidence of low quality; [i] contributing evidence of very low quality; [j] contributing evidence of high quality; [k] incoherence
